# Foxp and Skor family proteins control differentiation of Purkinje cells from Ptf1a- and Neurog1-expressing progenitors in zebrafish

**DOI:** 10.1242/dev.202546

**Published:** 2024-04-02

**Authors:** Tsubasa Itoh, Mari Uehara, Shinnosuke Yura, Jui Chun Wang, Yukimi Fujii, Akiko Nakanishi, Takashi Shimizu, Masahiko Hibi

**Affiliations:** Department of Biological Science, Graduate School of Science, Nagoya University, Furo, Chikusa, Nagoya, Aichi 464-8602, Japan

**Keywords:** Ptf1a, Neurogenin 1, Skor, Foxp, Purkinje cells, Zebrafish

## Abstract

Cerebellar neurons, such as GABAergic Purkinje cells (PCs), interneurons (INs) and glutamatergic granule cells (GCs) are differentiated from neural progenitors expressing proneural genes, including *ptf1a*, *neurog1* and *atoh1a/b/c*. Studies in mammals previously suggested that these genes determine cerebellar neuron cell fate. However, our studies on *ptf1a;neurog1* zebrafish mutants and lineage tracing of *ptf1a*-expressing progenitors have revealed that the *ptf1a/neurog1*-expressing progenitors can generate diverse cerebellar neurons, including PCs, INs and a subset of GCs in zebrafish. The precise mechanisms of how each cerebellar neuron type is specified remains elusive. We found that genes encoding the transcriptional regulators Foxp1b, Foxp4, Skor1b and Skor2, which are reportedly expressed in PCs, were absent in *ptf1a;neurog1* mutants. *foxp1b;foxp4* mutants showed a strong reduction in PCs, whereas *skor1b;skor2* mutants completely lacked PCs, and displayed an increase in immature GCs. Misexpression of *skor2* in GC progenitors expressing *atoh1c* suppressed GC fate. These data indicate that Foxp1b/4 and Skor1b/2 function as key transcriptional regulators in the initial step of PC differentiation from *ptf1a/neurog1-*expressing neural progenitors, and that Skor1b and Skor2 control PC differentiation by suppressing their differentiation into GCs.

## Teaser

**Summary:** Analysis of zebrafish mutants and lineage tracing of *ptf1a*-expressing progenitors reveal that Foxp and Skor family transcriptional regulators control the differentiation of Purkinje cells from neural progenitors expressing the proneural genes *ptf1a* and *neurog1*.

## INTRODUCTION

The structure of the cerebellum is conserved in most vertebrates. The cerebellum contains glutamatergic granule cells (GCs) and projection neurons, which are neurons in the deep cerebellar nuclei (DCN) in mammals or eurydendroid cells (ECs) in teleosts, and GABAergic Purkinje cells (PCs) and interneurons (INs), which include Golgi and stellate cells in both mammals and teleosts, such as zebrafish (Hashimoto and Hibi, 2012; Hibi et al., 2017; Hibi and Shimizu, 2012).

**Fig. 1.**
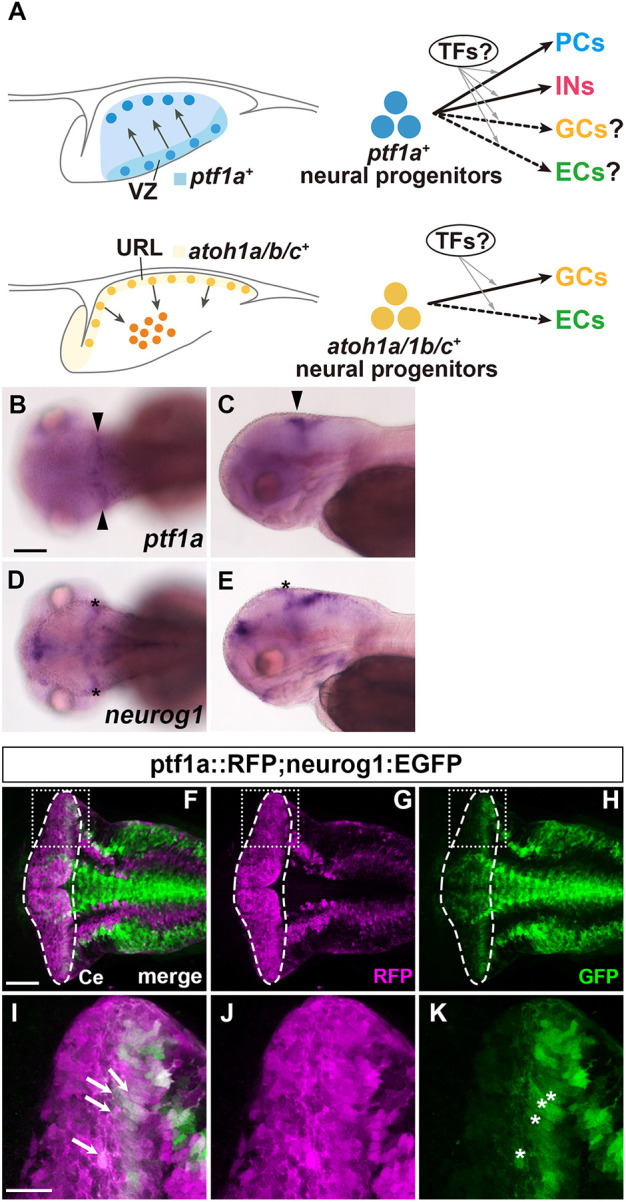
**Expression of *ptf1a* and *neurog1* in the cerebellum.** (A) Schematic of cerebellar neurogenesis. Development of cerebellar neurons from neural progenitors in the VZ and URL. TFs, transcription factors. (B-E) Expression of *ptf1a* (B,C) and *neurog1* (D,E) mRNA at 3 dpf, detected by *in situ* hybridization. Dorsal (B,D) and lateral views (C,E) with anterior to the left. Expression of *ptf1a* in the cerebellar ventricular zone is marked by arrowheads. Expression of *neurog1*, marked by asterisks, was observed in the tectum but not the cerebellum. (F-H) Detection of *ptf1a*- and/or *neurog1*-expressing cells using transgenic lines. 5-dpf *Tg(ptf1a:GAL4-VP16); Tg(UAS:RFP); Tg(neurog1:GFP)* larvae (*n*=3) were stained with anti-RFP (magenta) and anti-GFP (green) antibodies. *Tg(ptf1a:GAL4-VP16); Tg(UAS:RFP*) is referred to as ptf1a::RFP. Dorsal views of the rostral hindbrain region, including the cerebellum. The cerebellar region (Ce) is surrounded by a dashed line. (I-K) Higher magnification views of the boxed areas in E-G. ptf1a::RFP and neurog1:GFP double-positive cells are marked by white arrows (I) and the expression of neurog1:GFP*^+^* cells in the cerebellar ventricular zone is indicated by white asterisks (K). Scale bars: 100 μm (in B, for B-E); 50 μm (in F, for F-H); 20 μm (in I, for I-K).

Previous studies in mice revealed that these cerebellar neurons are derived from neural progenitors that express the proneural genes *Atoh1* or *Ptf1a* (Ben-Arie et al., 1997; Machold and Fishell, 2005; Wang et al., 2005; Wingate, 2005) (Fig. 1A). The *Atoh1*-expressing (*Atoh1^+^*) neural progenitors are located in the upper rhombic lip (URL, also called the cerebellar rhombic lip) and give rise to projection neurons in DCN and GCs in the cerebellum (Ben-Arie et al., 1997; Machold and Fishell, 2005; Wang et al., 2005; Wingate, 2005). By contrast, the *Ptf1a*-expressing (*Ptf1a^+^*) neural progenitors are located in the ventricular zone (VZ) and give rise to PCs and INs (Hoshino, 2012; Hoshino et al., 2005). In addition to *Ptf1a*, the proneural genes neurogenin 1 (*Neurog1*) and *Ascl1* are expressed in the VZ of the cerebellum and these proneural gene-expressing neural progenitors have been shown to give rise to PCs and INs (Lundell et al., 2009; Sudarov et al., 2011). Expression of *atoh1* (*atoh1a/b/c*) and *ptf1a* genes in the URL and VZ of the cerebellum has also been reported for zebrafish (Adolf et al., 2004; Chaplin et al., 2010; Kani et al., 2010; Köster and Fraser, 2001; Volkmann et al., 2008), suggesting similar or identical mechanisms by which proneural genes control the differentiation of cerebellar neurons. However, lineage tracing in zebrafish indicated that at least a portion of ECs may be derived from *ptf1a^+^* progenitors, suggesting that a slightly different mechanism between mammals and zebrafish may be involved in the differentiation of projection neurons (Kani et al., 2010).

Studies of mouse and zebrafish *atoh1* genes revealed that they are required for the differentiation of GCs (Ben-Arie et al., 1997; Kidwell et al., 2018). Similarly, a mouse *Ptf1a* mutant has been shown to completely lack PCs and INs (Hoshino et al., 2005) and the zebrafish *ptf1a* mutant shows a reduction – but not loss – of PCs (Itoh et al., 2020), indicating a requirement for *ptf1a* in PC development. VZ progenitor cells have been shown to generate GCs in *Ptf1a* mutant mice (Pascual et al., 2007). Ectopic expression of *Atoh1* or *Ptf1a* in mouse VZ or URL results in the generation of glutamatergic and GABAergic neurons, respectively (Yamada et al., 2014), suggesting that expression of *Atoh1* and *Ptf1a* is sufficient to determine the fate of these cell populations. However, it is still not clear whether these proneural genes irreversibly determine the fate of cells in the cerebellum. In the hindbrain region caudal to the cerebellum, *Ptf1a^+^* progenitors give rise to inhibitory neurons in the cochlear nuclei in mice (Fujiyama et al., 2009), excitatory neurons in the inferior olivary nuclei (IO neurons) in both mice and zebrafish (Itoh et al., 2020; Yamada et al., 2007), and crest cells in zebrafish (Itoh et al., 2020), indicating that *ptf1a^+^* progenitors have the potential to generate neurons other than GABAergic PCs or INs. It was previously shown that the homeodomain transcription factor Gsx2 is involved in fate determination of IO neurons (Itoh et al., 2020). The factors involved in the differentiation of PCs from *ptf1a^+^* progenitors in the cerebellum remain elusive.

Several transcription factors have been shown to be involved in the differentiation of PCs. The Forkhead transcription factors FOXP2 and FOXP4 are expressed in PCs in the mouse cerebellum (Ferland et al., 2003; Tam et al., 2011; Tanabe et al., 2012). In *Foxp2* mutant mice, although the specification of PCs takes place, positioning and dendrite formation of PCs are affected (Shu et al., 2005). siRNA-mediated knockdown of *Foxp4* at a late developmental period results in the impairment of PC dendrite formation (Tam et al., 2011). These findings suggest that Foxp family transcription factors regulate late processes of PC differentiation, but are not involved in early differentiation processes. Ski/Sno family transcriptional co-repressor 2 (Skor2, also known as Corl2) is expressed in PCs and plays an important role in the differentiation of PCs (Nakatani et al., 2014; Wang et al., 2011). *Skor2* mutant mice exhibit developmental defects in PC development with impaired dendrite arborization, decreased expression of PC marker genes, and increased expression of glutamatergic neuronal genes instead. However, *Skor2* has been shown to be dispensable for the specification and maintenance of PC fate (Nakatani et al., 2014; Wang et al., 2011). In addition to *Skor2*, *Skor1* is expressed in PCs but its role in PC differentiation remains elusive (Nakatani et al., 2014). Although these transcriptional regulators are involved in some aspects of PC differentiation, it is unclear whether these genes function downstream of Ptf1a and Neurog1. It is also not clear whether they control initial specification of PCs.

Previous RNA-sequencing analysis of zebrafish cerebellar neurons revealed that *foxp1b/4* and *skor1b/2* are expressed in developing PCs in the zebrafish cerebellum (Takeuchi et al., 2017). In this study, we show that *ptf1a^+^* neural progenitors are capable of generating not only PCs, but also INs, ECs and PCs, and that Foxp1b/4 and Skor1b/2 function downstream of Ptf1a and Neurog1 to control differentiation from Ptf1a/Neurog1-expressing neural progenitors into PCs.

## RESULTS

### Ptf1a and Neurog1 are co-expressed in cerebellar VZ progenitors

*ptf1a* is expressed in the cerebellar VZ and involved in the generation of PCs in mice and zebrafish (Hoshino et al., 2005; Kani et al., 2010). PCs are absent in mouse *Ptf1a* mutants, whereas PCs are reduced, but not absent, in zebrafish *ptf1a* mutants (Itoh et al., 2020). Lineage tracing in mice suggested that *Neurog1* is expressed in the progenitors of PCs in mice (Lundell et al., 2009). Therefore, *neurog1* is a candidate for a factor that can compensate for the loss of *ptf1a*. We compared the expression of *ptf1a* and *neurog1* by *in situ* hybridization and by using transgenic lines expressing fluorescent proteins (Fig. 1). As reported previously, *ptf1a* transcripts were detected in the cerebellar VZ in early-stage larvae [3 days post-fertilization (dpf) larvae; Fig. 1B,C], whereas *neurog1* transcripts were barely detected in the cerebellum region (Fig. 1D,E). However, the promoter and enhancer activity of *neurog1* was detected in the cerebellar VZ of *TgBAC(neurog1:EGFP)* (hereafter, named neurog1:EGFP) larvae (Fig. 1H,K). We compared neurog1:GFP-expressing cells with *ptf1a^+^* cells that were marked using the Gal4-UAS system with *TgBAC(ptf1a:GAL4-VP16)* and *Tg(UAS:RFP)* (referred to as ptf1a::RFP) (Fig. 1G,J). The expression pattern of ptf1a::RFP in VZ progenitor cells was the same as that of GFP in *Tg(ptf1a:GFP)* (Fig. S1). We found that some ptf1a::RFP-expressing cells also expressed neurog1:EGFP (Fig. 1F,I), suggesting that at least a subset of the *ptf1a^+^* neural progenitors also express *neurog1* in the cerebellar VZ.

### Ptf1a and Neurog1 cooperate to generate various cerebellar neurons

**Fig. 2.**
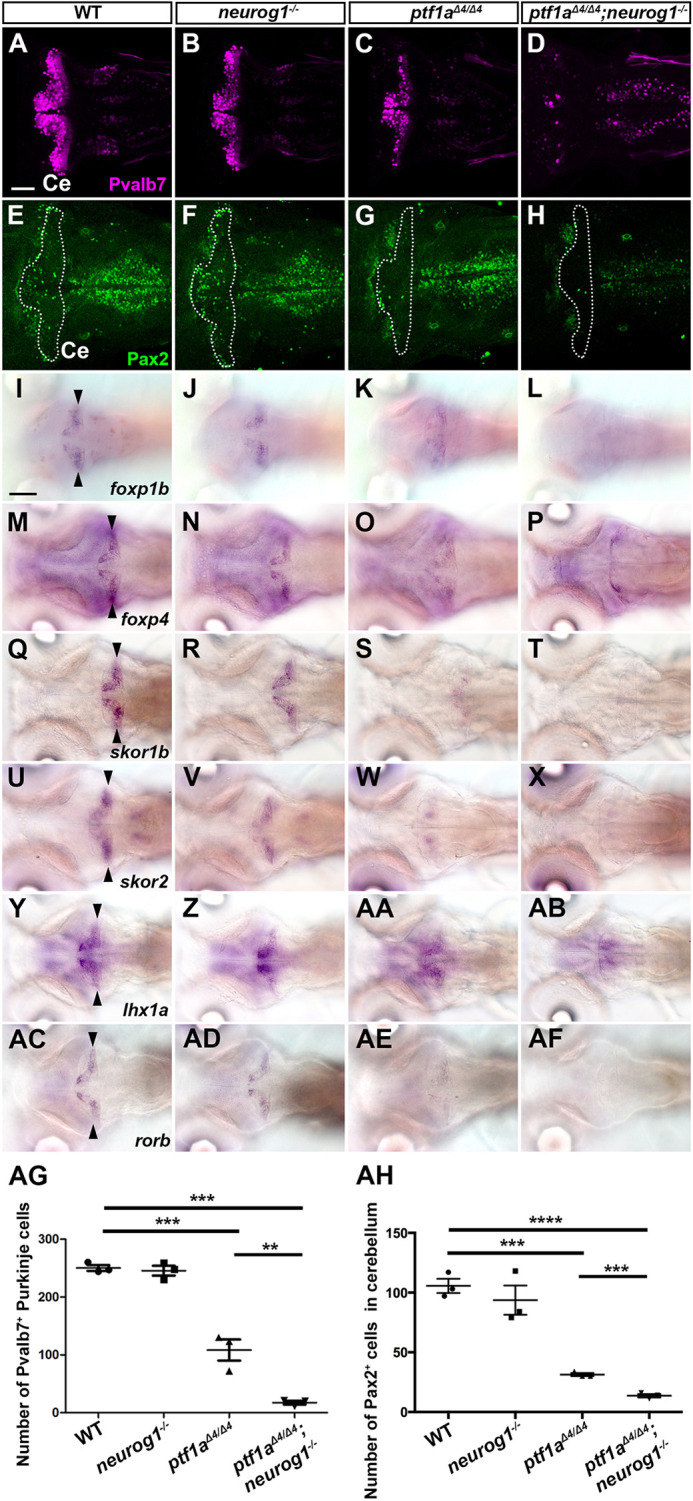
***ptf1a* and *neurog1* are required for the development of GABAergic PCs and INs.** (A-AF) Expression of parvalbumin 7 (Pvalb7, A-D), Pax2 (E-H), *foxp1b* (I-L), *foxp4* (M-P), *skor1b* (Q-T), *skor2* (U-X), *lhx1a* (Y-AB) and *rorb* (AC-AF) in the cerebellum of 5 dpf WT, *neurog1* mutant, *ptf1a* mutant and *ptf1a;neurog1* double-mutant larvae, revealed by immunostaining with anti-Pvalb7 (A-D) and anti-Pax2 (E-H) antibodies, or by *in situ* hybridization (I-AF). Dorsal views with anterior to the left. The cerebellum region (Ce) is surrounded by a dotted line (E-H). Pvalb7, *foxp1b/4*, *skor1b/2*, *lhx1a*, and *rorb* were expressed in PCs (expression of PC genes in the cerebellum is indicated by arrowheads). Pax2 is a marker of GABAergic INs. The number of examined larvae and larvae showing each expression pattern is given in Table 1. Scale bars: 50 μm (in A, for A-H); 100 μm (in I, for I-AF). (AG,AH) Number of Pvalb7^+^ PCs and Pax2^+^ INs in the cerebellum of 5 dpf WT, *neurog1*, *ptf1a* and *ptf1a;neurog1* mutant larvae. **P<0.01, ***P<0.001, ****P<0.0001 (ANOVA with Tukey's multiple comparison test). Data are shown as mean±s.e.m. with individual values indicated. *n*=3 for each genotype.

**Fig. 3.**
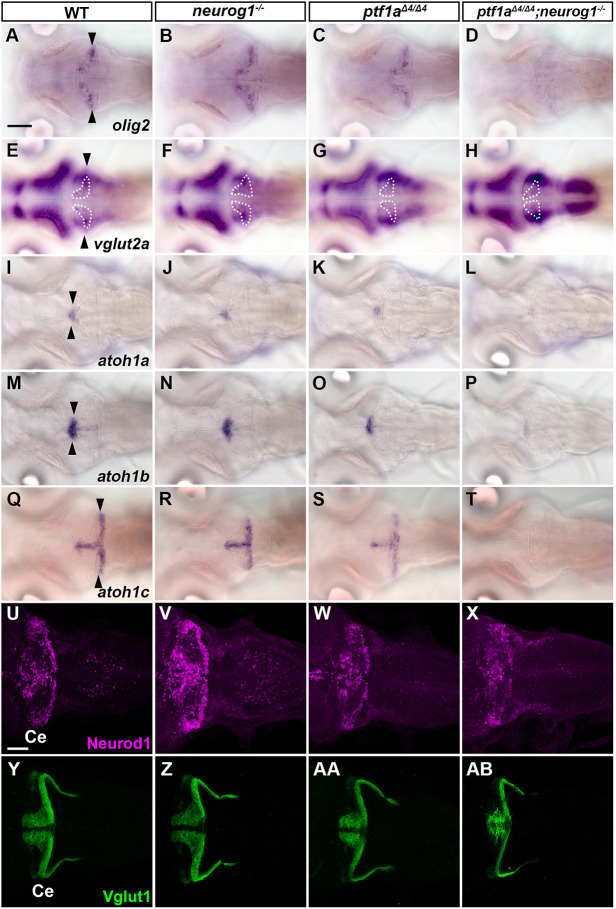
***ptf1a* and *neurog1* are involved in the development of ECs and GCs.** (A-T) Expression of *olig2* (A-D), *vglut2a* (E-H), *atoh1a* (I-L), *atoh1b* (M-P) and *atoh1c* (Q-T) in 5 dpf WT, *neurog1*, *ptf1a* and *ptf1a;neurog1* mutant larvae, revealed by *in situ* hybridization. *olig2* and *vglut2a* were expressed in ECs. *atoh1a/b/c* were expressed in GC progenitors. Arrowheads indicate the expression in the cerebellum. The expression area of *vglut2a* is surrounded by a dotted line (E-H). (U-AB) Expression of the GC markers Neurod1 and Vglut1 in 5 dpf WT, *neurog1*, *ptf1a* and *ptf1a;neurog1* mutant larvae, revealed by immunostaining. The expression pattern of Neurod1 and Vglut1 was affected in *ptf1a* and *ptf1a;neurog1* mutants, but the area of Neurod1-expression domains was variable in *ptf1a* mutants. The number of examined larvae and larvae showing each expression pattern is given in Table 1. Scale bars: 100 μm (in A, for A-T); 50 μm (in U, for U-AB).

To reveal the roles of *ptf1a* and *neurog1* in cerebellar neurogenesis, we generated combined mutants of *ptf1a*^Δ*4*^ and *neurog1^hi1059Tg^* (referred to as *neurog1*^−^) alleles (Figs 2 and 3) (Golling et al., 2002; Itoh et al., 2020) and analyzed their phenotypes by marker expression. Whereas *neurog1* mutant larvae had comparable numbers of PCs and INs, which were marked by Parvalbumin 7 (Pvalb7) and Pax2, compared with wild-type (WT) larvae, *ptf1a* mutants showed a significant reduction in PCs and INs (Fig. 2A-C,E-G,AG,AH, Table 1). The *neurog1* mutation enhanced *ptf1a* mutant phenotypes and *ptf1a;neurog1* double-mutant larvae showed an almost complete lack of PCs and INs (Fig. 2D,H,AG,AH). Consistent with this, *ptf1a* mutants showed a reduced expression of genes that are reportedly expressed in zebrafish PCs (Takeuchi et al., 2017), including *foxp1b/4*, *skor1b/2*, *lhx1a*, and *rorb*. *ptf1a;neurog1* mutants displayed a lack of expression of these PC genes (Fig. 2I-AF). A similar reduction and loss of crest cells in the anterior hindbrain, which receive GC axons and function in the cerebellum-like structure (Hibi and Shimizu, 2012), was observed in *ptf1a* and *ptf1a;neurog1* mutants, respectively (Fig. S2). These data indicate that *ptf1a* plays a major role in the development of PCs and INs in the cerebellum and crest cells of the rostral hindbrain, but that *neurog1* is not essential for this development, although it has some redundant functions that overlap with those of *ptf1a*.

**Table 1.**
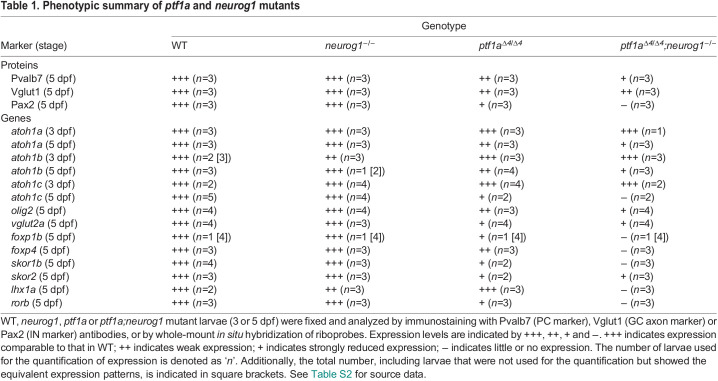
Phenotypic summary of *ptf1a* and *neurog1* mutants

In addition to the PC and IN markers, the expression of *olig2* and *vglut2a* (*slc17a6b*), which are expressed in ECs (Bae et al., 2009; Kani et al., 2010; McFarland et al., 2008), was decreased in the *ptf1a* mutant cerebellum, and further decreased in the *ptf1a;neurog1* mutant cerebellum (Fig. 3A-H). Furthermore, the expression of *atoh1a*, *atoh1b* and *atoh1c*, which are expressed in the GC progenitors (Chaplin et al., 2010; Kani et al., 2010; Kidwell et al., 2018), was not affected at 3 dpf (Fig. S3), but was reduced at 5 dpf in *ptf1a;neurog1* mutant larvae (Fig. 3I-T). *ptf1a;neurog1* mutant larvae had a variable number of cells expressing GC markers Neurod1 and Vglut1 (Slc17a7a) at 5 dpf (Fig. 3X,AB). These data indicate that Ptf1a and Neurog1 are not absolutely essential for the development of glutamatergic ECs and GCs, but are at least partly involved in their development.

### Ptf1a-expressing neural progenitors give rise to a variety of cerebellar neurons

**Fig. 4.**
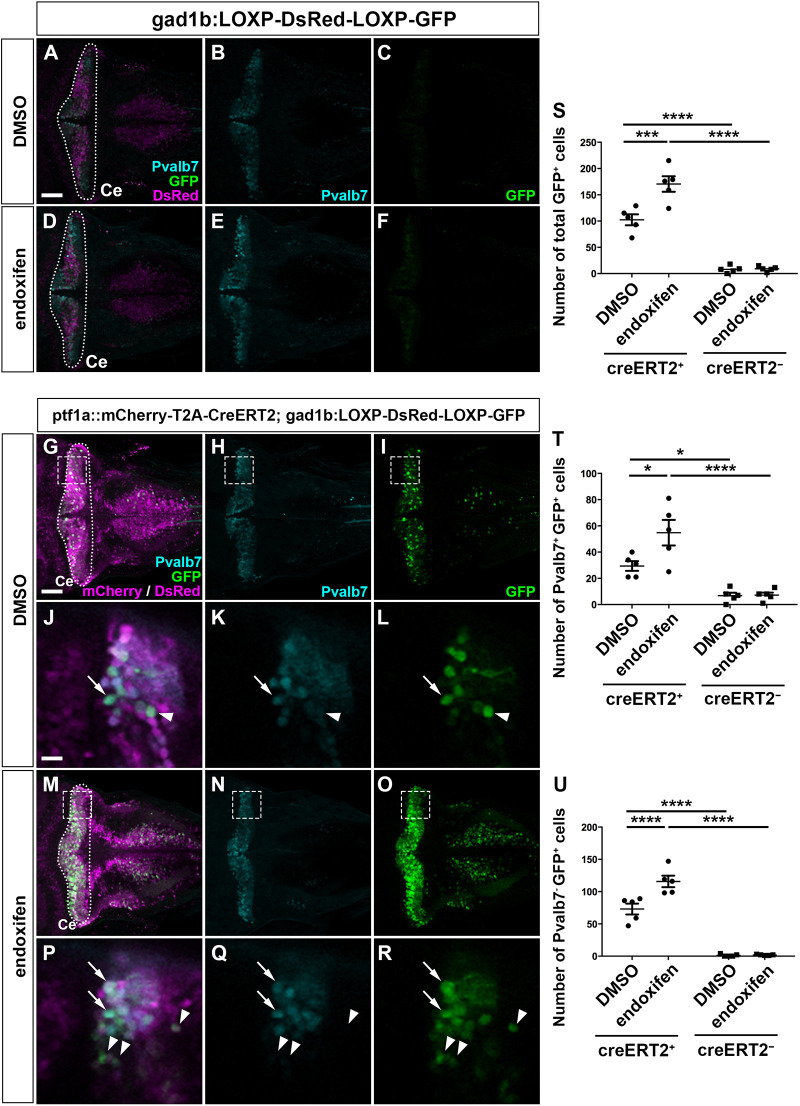
**GABAergic PCs and INs are derived from Ptf1a-expressing neural progenitors.** (A-F) Expression of Pvalb7 and GFP in 5 dpf *TgBAC(gad1b:LOXP-DsRed-LOXP-GFP)* larvae that were treated with DMSO (control, *n*=5; A-C) or endoxifen (*n*=5; D-F) at 2 dpf. (G-R) Expression of Pvalb7 and GFP in 5 dpf *TgBAC(ptf1a:Gal4-VP16); Tg(UAS-hsp70l:mCherry-T2A-CreERT2); TgBAC(gad1b:LOXP-DsRed-LOXP-GFP)* larvae that were treated with DMSO (*n*=5; G-L) or endoxifen (*n*=5; M-R) at 2 dpf. The larvae were stained with anti-Pvalb7 (cyan), anti-RFP (magenta) and anti-GFP (green) antibodies. Dorsal views with anterior to the left. The cerebellum region (Ce) is surrounded by a dotted line. (J-L,P-R) Higher magnification views of the boxed areas in G-I,M-O. Arrows and arrowheads indicate Pvalb7^+^ GFP^+^ cells (PCs) and Pvalb7^−^ GFP^+^ cells (INs), respectively. Scale bars: 50 μm (in A, for A-F; in G, for G-I,M-O); 10 μm (in J, for J-L,P-R). (S-U) Total number of GFP^+^ cells (S), Pvalb7^+^ GFP^+^ cells (T) and Pvalb7^−^ GFP^+^ cells (U) in the cerebellum of larvae treated with DMSO or endoxifen. *P<0.05, ***P<0.001, ****P<0.0001 (two-way ANOVA followed by Bonferroni multiple comparisons). Data are shown as mean±s.e.m. with individual values indicated.

**Fig. 5.**
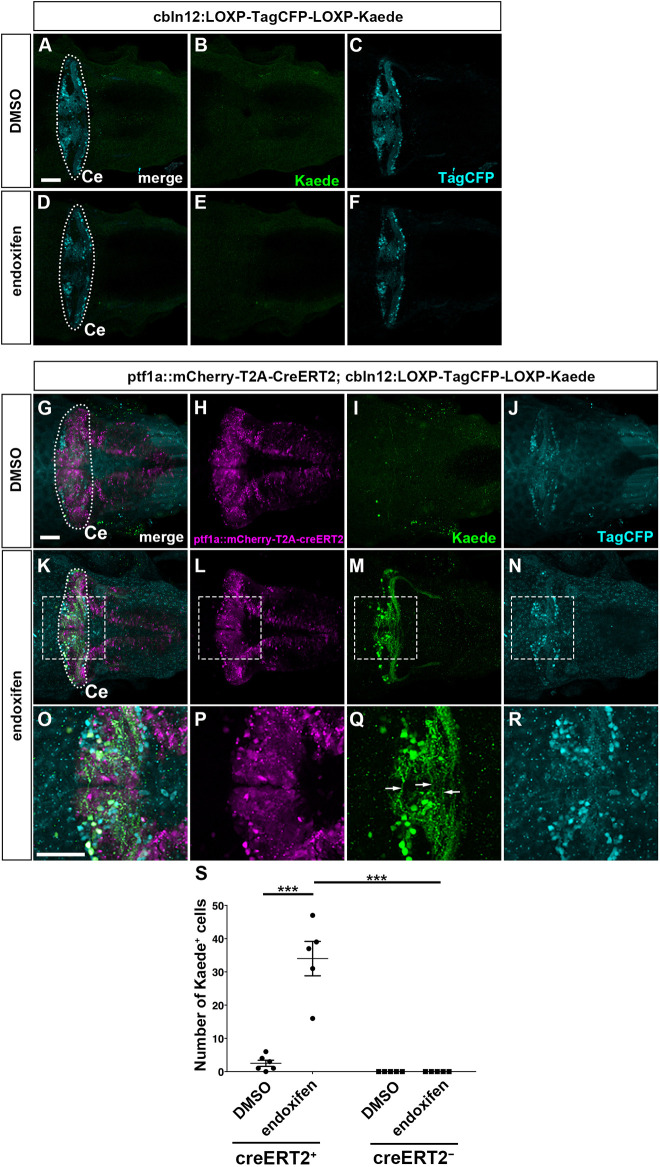
**Some GCs are also derived from Ptf1a-expressing neural progenitors.** (A-F) Expression of TagCFP (cyan) and Kaede (green) in 5 dpf *Tg(cbln12:LOXP-TagCFP-LOXP-Kaede)* larvae that were treated with DMSO (control, *n*=5; A-C) or endoxifen (*n*=5; D-F) at 2 dpf. (G-R) Expression of TagCFP and Kaede in 5 dpf *TgBAC(atoh1c:Gal4FF); Tg(UAS-hsp70l:RFP-T2A-CreERT2); Tg(cbln12:LOXP-TagCFP-LOXP-Kaede)* larvae that were treated with DMSO (*n*=6; G-J) or endoxifen (*n*=5; K-R) at 2 dpf. The larvae were stained with anti-TagCFP (cyan), anti-RFP (magenta) and anti-Kaede (green) antibodies. Dorsal views with anterior to the left. The cerebellum region (Ce) is surrounded by a dotted line. (O-R) Higher magnification views of the boxed areas in K-N. Arrows indicate parallel fibers of GCs. Scale bars: 50 μm (in A, for A-F; in G, for G-N; in O, for O-R). (S) Number of Kaede^+^ cells in the cerebellum of larvae treated with DMSO or endoxifen. ***P<0.001 (two-way ANOVA followed by Bonferroni multiple comparisons). Data are shown as mean±s.e.m. with individual values indicated.

We next traced the *ptf1a^+^* cell lineage (Figs 4 and 5). We expressed mCherry and CreERT2 in *ptf1a^+^* cells using the Gal4-UAS system with *TgBAC(ptf1a:GAL4-VP16)* and *Tg(UAS-hsp70l:mCherry-T2A-CreERT2)* lines (referred to as ptf1a::mCherry-T2A-CreERT2). To validate the expression of mCherry and CreERT2 in *ptf1a*^+^ cells, we generated a *ptf1a^Tg(hsp70l-EGFP)^* line by knocking in EGFP expression cassette at the *ptf1a* gene locus. EGFP expression in the *ptf1a^Tg(hsp70l-EGFP)^* line recapitulated *ptf1a* expression (Fig. S4). In the cerebellum, cells expressing mCherry in the ptf1a::mCherry-T2A-CreERT2 line overlapped with those expressing EGFP in the *ptf1a^Tg(hsp70l-EGFP)^* line and coincided with *CreERT2* mRNA-expressing cells (Fig. S4), confirming the expression of CreERT2 in *ptf1a*^+^ cells. The reporter line *TgBAC(gad1b:LOXP-DsRed-LOXP-GFP)* (Satou et al., 2013) was used to trace GABAergic neurons. In this experiment, when CreERT2 was expressed in *ptf1a^+^* cells and activated with endoxifen, CreERT2 induced recombination of the reporter gene, resulting in the conversion from DsRed to GFP expression in GABAergic neurons. GFP-expressing (GFP^+^) cells are therefore identified as GABAergic neurons derived from *ptf1a^+^* neural progenitors. In the absence of CreERT2 expression, only a small number of GFP^+^ cells was observed (Fig. 4A-F,S), whereas a significant number of GFP^+^ cells was observed in the cerebellum in the presence of ptf1a::mCherry-T2A-CreERT2 (Fig. 4I,L). Endoxifen treatment increased the number of GFP^+^ cells (Fig. 4O,R,S). It is likely that the expression of GFP in the absence of endoxifen treatment is due to the strong expression of CreERT2 and leakiness of the reporter. The increase in GFP^+^ cells by endoxifen at 2 dpf, when the expression domains of *ptf1a* and *atoh1a* are completely separated from each other in the cerebellum (Kani et al., 2010), indicates that most, if not all, GFP^+^ cells are derived from neural progenitors expressing *ptf1a* but not *atoh1* at 2 dpf. Two types of GFP^+^ cells were identified: Pvalb7-expressing (Pvalb7^+^) and Pvalb7-negative (Pvalb7^−^) cells (Fig. 4P-R), which correspond to PCs and INs, respectively. The numbers of both GFP^+^ Pvalb7^+^ and GFP^+^ Pvalb7^−^ cells in 5 dpf larvae harboring CreERT2 and the reporter were increased by endoxifen treatment (Fig. 4T,U), indicating that the increased PCs and INs were derived from *ptf1a^+^* neural progenitors at 2 dpf.

We further examined the GC lineage derived from *ptf1a^+^* neural progenitors using the reporter line *Tg(cbln12:LOXP-TagCFP-LOXP-Kaede)* (Fig. 5C), which expresses TagCFP in GCs in a *cbln12* promoter-dependent manner (Dohaku et al., 2019). In this experiment, the expression and activation of CreERT2 induced recombination of the reporter gene, resulting in a conversion from TagCFP to Kaede expression in GCs. Kaede-expressing (Kaede^+^) cells can be concluded to be GCs derived from *ptf1a^+^* neural progenitors. Kaede was barely detected in larvae with only the reporter gene (Fig. 5B,E), and in larvae with both CreERT2 and reporter genes but no endoxifen treatment (Fig. 5I), indicating that this reporter had very low leakiness. Endoxifen treatment at 2 dpf resulted in the appearance of Kaede^+^ cells that extended typical parallel fibers (Fig. 5M,Q). These data indicate that a portion of GCs in the cerebellum was derived from *ptf1a^+^* neural progenitors in zebrafish. Considering the data for both *ptf1a* and *neurog1* mutants, we conclude that Ptf1a/Neurog1-expressing neural progenitors are capable of generating a variety of cerebellar neurons.

### Foxp1b/4 and Skor1b/2 function downstream of Ptf1a and Neurog1 in differentiating PCs

**Fig. 6.**
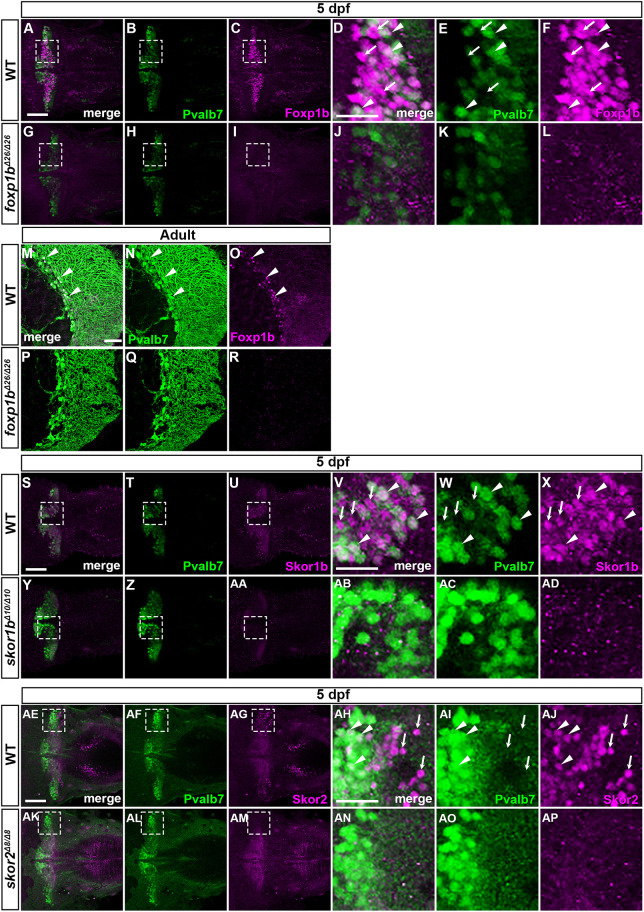
**Foxp1b, Skor1b and Skor2 are expressed in differentiating and differentiated PCs.** (A-R) Localization of Foxp1b. 5 dpf WT (*n*=3; A-F) and *foxp1b* mutant (*n*=3; G-L) larvae, and adult WT (*n*=2: M-O) and *foxp1b* mutant (*n*=2; P-R) cerebellum sections immunostained with anti-Foxp1b (magenta) and anti-Pvalb7 antibodies (green). Dorsal views with anterior to the left (A-L) and sagittal sections (M-R). (D-F,J-L) Higher magnification views of the boxed areas in A-C,G-I. Arrowheads and arrows indicate examples of Foxp1b^+^ Pvalb7^+^ cells and Foxp1b^+^ Pvalb7^−^ cells, respectively (D-F,M-O). (S-AP) Localization of Skor1b and Skor2. (S-AD) 5 dpf WT (*n*=3; S-X) and *skor1b* mutant larvae (*n*=3; Y-AD) immunostained with anti-Skor1b (magenta) and anti-Pvalb7 antibodies (green). (AE-AP) 5 dpf WT (*n*=2; AE-AJ) and *skor2* mutant larvae (*n*=2; AK-AP) immunostained with anti-Skor2 (magenta) and anti-Pvalb7 antibodies (green). Dorsal views with anterior to the left. (V-X,AB-AD,AH-AJ,AN-AP) Higher magnification views of the boxed areas in S-U,Y-AA,AE-AG,AK-AM. Scale bars: 50 μm (in A, for A-C,G-I; in D, for D-F,J-L; in M, for M-R; in S, for S-U,Y-AA; in V, for V-X,AB-AD; in AE, for AE-AG,AK-AM; in AH, for AH-AJ,AN-AP). Arrowheads indicate examples of Skor1b^+^ Pvalb7^+^ cells (V-X) and Skor2^+^ Pvalb7^+^ cells (AH-AJ). Arrows indicate examples of Skor1b^+^ Pvalb7^−^ cells (V-X) and Skor2^+^ Pvalb7^−^ cells (AH-AJ).

There should be regulators that control the specification and/or differentiation of PCs from Ptf1a/Neurog1-expressing neural progenitors. We previously identified genes that were preferentially expressed in larval PCs (Takeuchi et al., 2017). Of these genes, we here focus on those encoding transcriptional regulators. Using *in situ* hybridization, we found that the Foxp family genes *foxp1b* and *foxp4* and the Skor family genes *skor1b* and *skor2* were expressed in the cerebellum from 2 dpf (Fig. S5). These genes were expressed in PCs in 5 dpf WT larvae, but were absent in *ptf1a;neurog1* double-mutant larvae (Fig. 2L,P,T,X), suggesting that these genes function downstream of Ptf1a and Neurog1. We generated antibodies against Foxp1b, Skor1b and Skor2 and used them to analyze their expression by co-immunostaining with anti-Pvalb7 antibody. Foxp1b was detected in the nucleus of Pvalb7^+^ PCs as well as in Pvalb7^−^ cells in the cerebellum of WT larvae (Fig. 6A-F), but was not observed in the cerebellum of *foxp1b* mutant larvae (Fig. 6G-L) (the *foxp1b* mutant is described below). Foxp1b was also detected in the nucleus of PCs in the WT adult cerebellum, but not in the *foxp1b* mutant cerebellum (Fig. 6O,R). Both Skor1b and Skor2 were detected in the nucleus of Pvalb7^+^ PCs and Pvalb7^−^ cells in the larval but not adult cerebellum (Fig. 6S-X,AE-AJ), but were not observed in *skor1b* and *skor2* mutant larvae (Fig. 6Y-AD,AK-AP) (*skor1b* and *skor2* mutants are described below). Although the possibility that Foxp1b, Skor1b and Skor2 are expressed in non-PC lineage cells of the cerebellum cannot be excluded, the data suggest that these proteins are expressed in PC lineage cells before PCs become fully differentiated.

### Foxp1b/4 and Skor1b/2 are required for the differentiation of PCs

We generated mutants of *foxp1b/4* and *skor1b/2* using the CRISPR/Cas9 method (Fig. S6). The *foxp1b* and *foxp4* mutants harbor 26- and 7-bp deletions in exon 14 of *foxp1b* and exon 7 of *foxp4*, respectively, which introduce a premature stop codon. The putative mutant Foxp1b and Foxp4 proteins lacked the DNA-binding forkhead domain. The *skor1b* and *skor2* mutants harbor 10- and 8-bp deletions in exon 1 of *skor1b* and exon 2 of *skor2*, respectively, which introduce a premature stop codon. Although the functional domains of Skor proteins were not well understood, the putative mutant Skor1b and Skor2 proteins lacked the protein from the c-Ski SMAD-binding domain to the carboxy terminus. The mutations in *foxp1b* and *foxp4* did not alter the expression of either gene, and similarly the mutations in *skor1b* and *skor2* did not influence the expression of either gene at 5 dpf (Fig. S7). This suggests that these mutations did not induce nonsense-mediated RNA decay or compensatory gene expression.

**Fig. 7.**
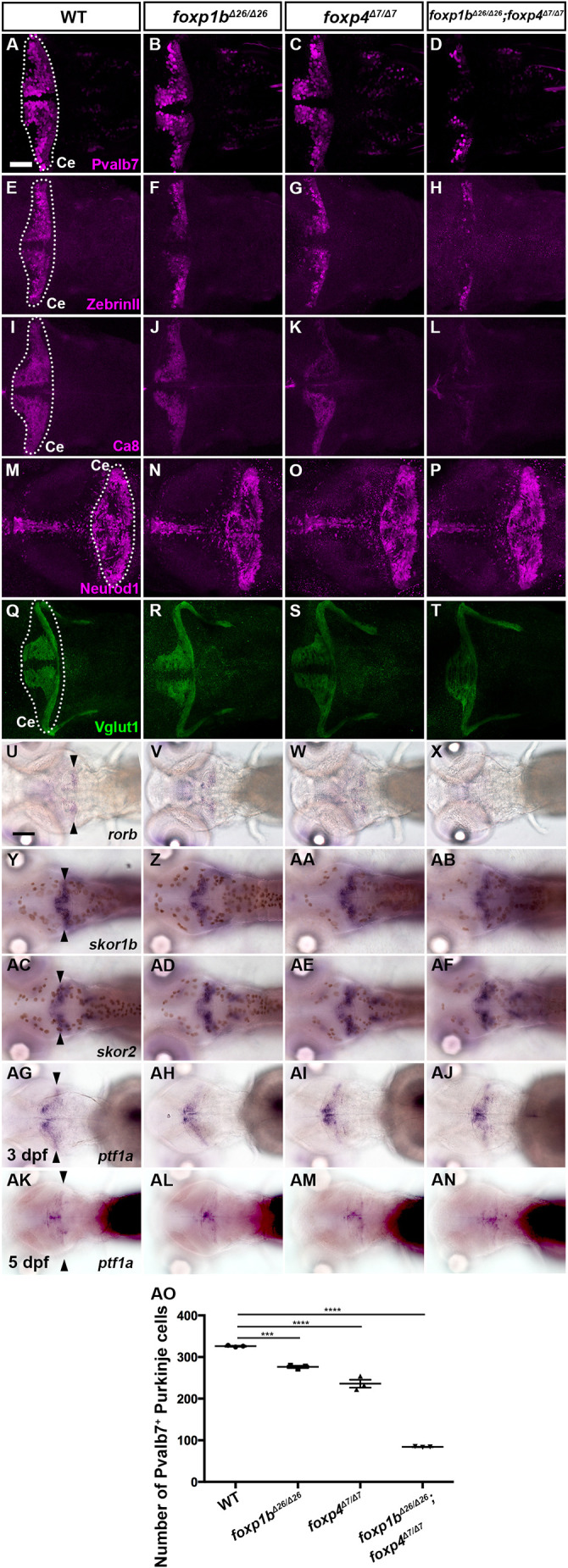
**Phenotypes of *foxp1b* and *foxp4* mutants.** (A-T) Expression of the PC markers Pvalb7, Zebrin II and Ca8, and the GC markers Neurod1 and Vglut1 in 5 dpf WT, *foxp1b*, *foxp4* and *foxp1b;foxp4* mutant larvae, revealed by immunostaining. The cerebellum region (Ce) is surrounded by a dotted line. (U-AN) Expression of *rorb*, *skor1b*, *skor2* and *ptf1a* in 5 dpf WT, *foxp1b*, *foxp4* and *foxp1b;foxp4* mutant larvae (U-AF,AK-AN) and expression of *ptf1a* in 3 dpf WT, *foxp1b*, *foxp4* and *foxp1b;foxp4* mutant larvae (AG-AJ), revealed by *in situ* hybridization. Dorsal views with anterior to the left. Arrowheads indicate expression of genes in the cerebellum. The number of examined larvae and larvae showing each expression pattern is shown in Table 2. Scale bars: 50 μm (in A, for A-T); 100 μm (in U, for U-AN). (AO) Number of Pvalb7^+^ PCs in the cerebellum of 5 dpf WT, *foxp1b*, *foxp4* and *foxp1b;foxp4* mutant larvae. ***P<0.001, ****P<0.0001 (ANOVA with Tukey's multiple comparison test). Data are shown as mean±s.e.m. with individual values indicated.

Single-mutant larvae of *foxp1b* or *foxp4* showed a slight reduction in the expression of Pvalb7, Zebrin II (encoded by *aldolase Ca* gene), carboxy anhydrase 8 (Ca8), or *rorb* in the cerebellum (Fig. 7A-C,E-G,I-K,U-W). The *foxp1b;foxp4* double mutant displayed a more severe reduction in these PC markers (Fig. 7D,H,L,X). After counting the number of Pvalb7^+^ PCs in the mutants, it was confirmed that PCs were slightly reduced in *foxp1b* and *foxp4* single mutants compared with WT, but were more severely reduced in *foxp1b;foxp4* double mutants (Fig. 7AO). Reduction of Pvalb7^+^ PCs was also observed in *foxp1b;foxp4* double crispants (F0 larvae), which have insertion/deletion (indel) mutations in target DNA different from the stable mutants described above (Fig. S8). In contrast to the PC markers, expression of the GC marker Neurod1, the EC markers *olig2* and *vglut2a*, and the IN marker *pax2a* was not affected in either single or double mutants (Fig. 7M-P, Fig. S9, Table 2). The expression of Vglut1 was altered in *foxp1b;foxp4* mutants; however, this is due to a significant reduction in PCs in these mutants, leading to abnormalities in GC axonal trajectory, and the size of the Vglut1 expression domain remained unchanged (Fig. 7Q,T, Table 2). These data suggest that Foxp1b and Foxp4 function partially redundantly in PC differentiation; Foxp1b and Foxp4 are required for the proper differentiation of PCs but not GCs, ECs or INs, in the cerebellum.

**Table 2.**
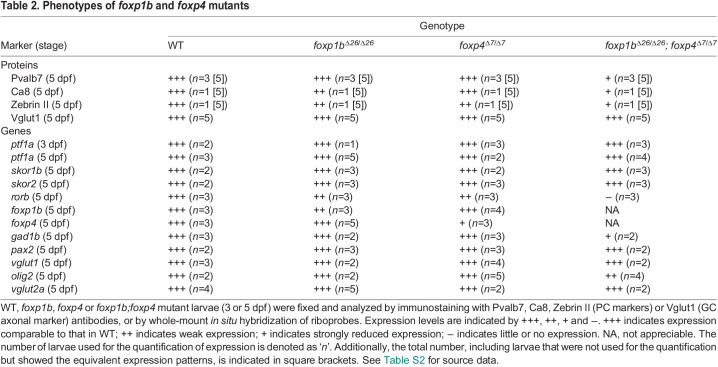
Phenotypes of *foxp1b* and *foxp4* mutants

**Fig. 8.**
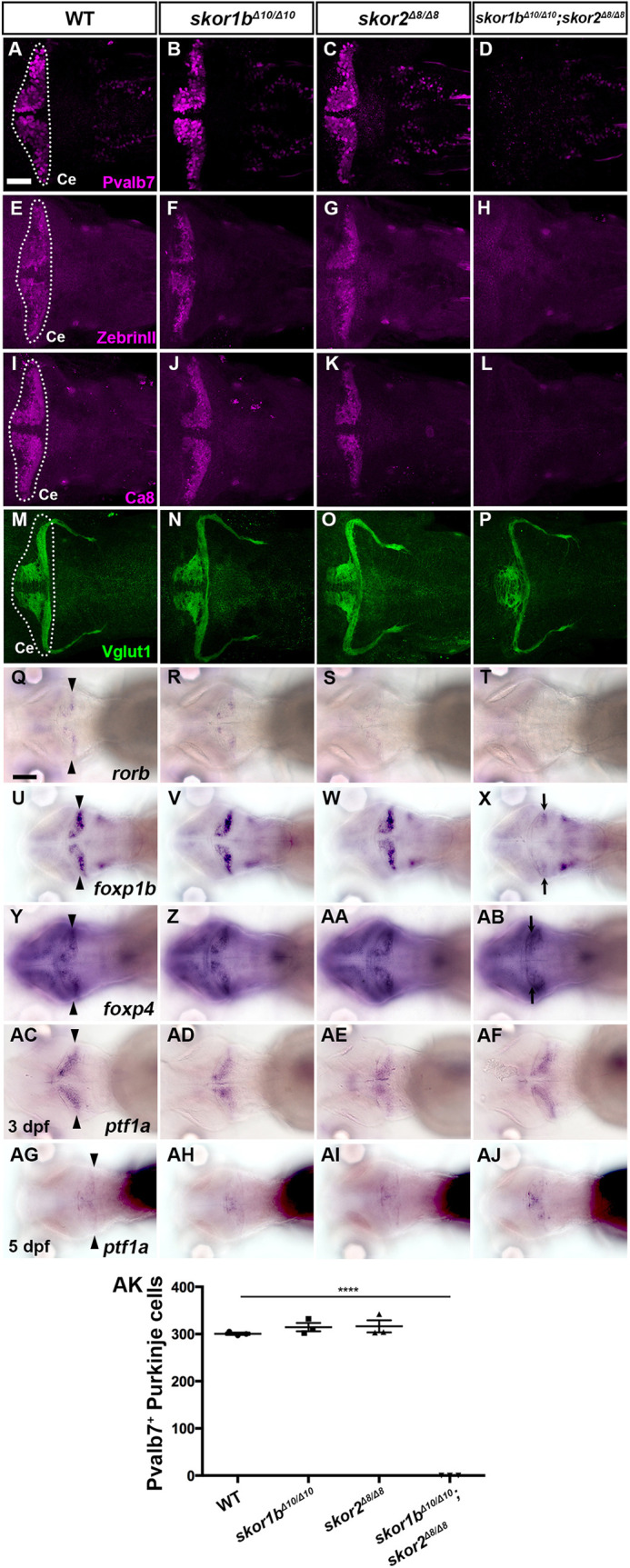
**Phenotypes of *skor1b* and *skor2* mutants.** (A-P) Expression of the PC markers Pvalb7, Zebrin II and Ca8, and the GC marker Vglut1 in 5 dpf WT, *skor1b*, *skor2* and *skor1b;skor2* mutant larvae, revealed by immunostaining. The cerebellum region (Ce) is surrounded by a dotted line. (Q-AJ) Expression of *rorb*, *foxp1b*, *foxp4* and *ptf1a* in 5 dpf WT, *skor1b*, *skor2* and *skor1b;skor2* mutant larvae (Q-AB,AG-AJ) and expression of *ptf1a* in 3(Q-AB,AG-AJ) dpf WT, *skor1b*, *skor2*, and *skor1b;skor2* mutant larvae (AC-AF), revealed by *in situ* hybridization. Dorsal views with anterior to the left. Arrowheads indicate expression of genes in the cerebellum. Arrows indicate expression of *foxp1b* and *foxp4* in caudal and rostral parts of the cerebellum (X,AB). The number of examined larvae and larvae showing each expression pattern is shown in Table 3. Scale bars: 50 μm (in A, for A-P); 100 μm (in Q, for Q-AJ). (AK) Number of Pvalb7^+^ PCs in the cerebellum of 5 dpf WT, *skor1b*, *skor2* and *skor1b;skor2* mutant larvae. ****P<0.0001 (ANOVA with Tukey's multiple comparison test). Data are shown as mean±s.e.m. with individual values indicated.

Single-mutant *skor1b* and *skor2* larvae did not show reduced expression of the PC markers compared with WT larvae (Fig. 8A-C,E-G,I-K,Q-S,AK), whereas *skor1b;skor2* double-mutant larvae showed a complete loss of expression of the PC markers (Pvalb7, Zebrin II, Ca8, *rorb*; Fig. 8D,H,L,T,AK). Similarly, a strong reduction or loss of Pvalb7^+^ PCs was observed in *skor1b;skor2* crispants, which have indel mutations in target DNA different from the stable mutants (Fig. S10). The expression of the GC axon marker Vglut1 was altered in *skor1b;skor2* mutants. This change is likely attributable to the absence of PCs in these mutants, leading to abnormalities in the GC axonal trajectory. The size of the Vglut1 expression domain remained unaffected in these mutants (Fig. 8P, Table 3). Expression of the EC markers *olig2* and *vglut2a* and the IN marker *pax2a* was not affected in either *skor1b*, *skor2* single or *skor1b;skor2* double mutants (Fig. S9, Table 3). These data indicate that Skor1b and Skor2 function redundantly and are essential for the differentiation of PCs, but not ECs or INs, in the cerebellum.

**Table 3.**
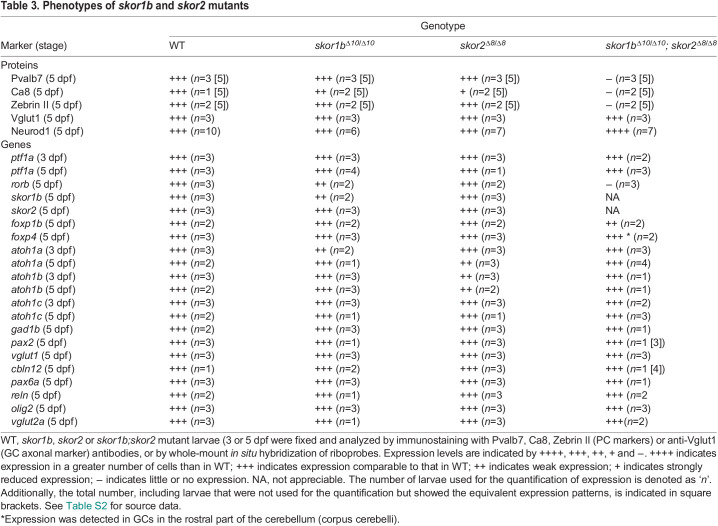
Phenotypes of *skor1b* and *skor2* mutants

Although *foxp1b;foxp4* and *skor1b;skor2* mutant larvae showed defects in PC development, expression of *skor1b* and *skor2* was not affected in *foxp1b;foxp4* mutant larvae (Fig. 7Y-AF, Table 2). The expression domains of *foxp1b* and *foxp4* in *skor1b;skor2* double-mutant larvae were altered by aberrant differentiation of cerebellar neurons, as described below. In *skor1b;skor2* mutants, the expression of *foxp1b* was strongly reduced, whereas the expression level of *foxp4* expression remained relatively unaffected. However, *foxp4* was ectopically observed in the rostral part of the cerebellum (Fig. 8X, AB, Table 3). These data suggest that *skor1b/2* expression is regulated independently of *foxp1b/4*, whereas *foxp1b/4* expression is partly or indirectly regulated by *skor1b/2* in the cerebellum. We further examined *ptf1a* expression in these mutants. *ptf1a* expression was not affected in *foxp1b;foxp4* mutants and *skor1b;skor2* mutants (Figs 7AG-AN, 8AC-AJ, Tables 2 and 3). These data suggest that *foxp1b*/*4* and *skor1b/2* regulate cerebellar neurogenesis independently of *ptf1a* expression.

### Skor1b and Skor2 suppress GC fate

**Fig. 9.**
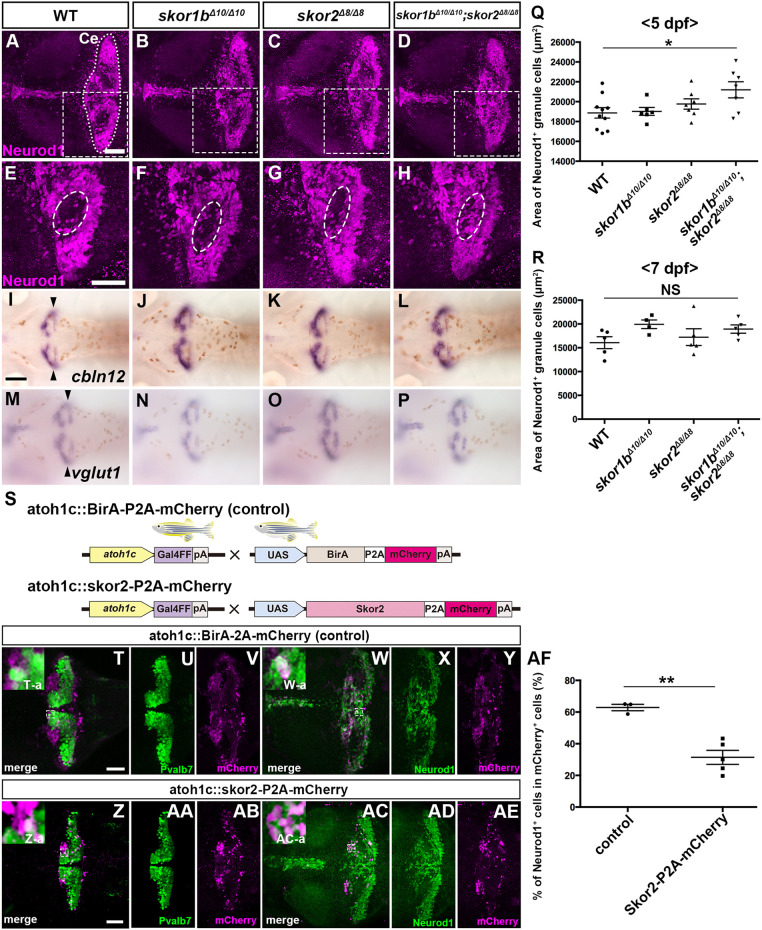
**Suppression of granule cell fates by Skor1b/2 and Foxp1b/4.** (A-H) Expression of Neurod1 in 5 dpf WT, *skor1b*, *skor2* and *skor1b;skor2* mutant larvae, revealed by immunostaining. The cerebellum region is surrounded by a dotted line. (E-H) Higher magnification views of the boxed areas in A-D. Neurod1-expressing GCs were absent in the central areas of the cerebellum (marked by dashed circles) of WT, *skor1b* and *skor2* mutant larvae, but present in the entire cerebellum of *skor1b;skor2* mutant larvae. (I-P) Expression of the mature GC marker genes *cbln12* and *vglut1* in the cerebellum, revealed by *in situ* hybridization. (Q,R) Area of Neurod1^+^ GCs in the cerebellum of 5 dpf (Q) or 7 dpf (R) WT, *skor1b*, *skor2* and *skor1b;skor2* mutants. *P<0.05 (ANOVA with Tukey's multiple comparison test). (S) Diagram of ectopic expression of biotin ligase A (BirA, control) or Skor2 in GC progenitors. (T-AE) Misexpression of Skor2 in *atoh1c*-expressing neural progenitors. 5 dpf *Tg(atoh1c:Gal4FF);Tg(UAS-hsp70l:BirA-P2A-mCherry)* or *Tg(atoh1c:Gal4FF);Tg(UAS:HA-skor2-P2A-mCherry)* larvae, which express BirA/mCherry or Skor2/mCherry in the GC lineage, were immunostained with anti-RFP/mCherry (magenta), and Pvalb7 (green; T-V,Z-AB) or Neurod1 (green; W-Y,AC-AE) antibodies. Dorsal views with anterior to the left (A-P,T-AE). Insets (T-a,W-a,Z-a,AC-a) show higher magnification views of the boxed areas in T,W,Z,AC. Scale bars: 50 μm (in A, for A-D; in E, for E-H; in T, for T-Y; in Z for Z-AE); 100 μm (in I, for I-P). (AF) Percentage of Neurod1^+^ cells out of total mCherry^+^ cells in the cerebellum of larvae expressing BirA (control) or Skor2. **P<0.01 (unpaired two-tailed Student's *t*-test). Data are shown as mean±s.e.m. with individual values indicated (Q,R,AF).

We further examined the expression of GC markers in *skor1b;skor2* mutant larvae at 5 dpf in more detail. WT, *skor1b* or *skor2* single-mutant larvae had regions of the cerebellum where Neurod1 expression was absent (Fig. 9A-C,E-G), whereas *skor1b;skor2* mutant larvae did not (Fig. 9D,H). Consistent with this finding, the area of the cerebellum containing Neurod1^+^ GCs was significantly larger in *skor1b*;*skor2* mutant larvae (Fig. 9Q), indicating that immature (Neurod1^+^) GCs increased in the *skor1b*;*skor2* mutant cerebellum. Cell proliferation, indicated by phospho-histone 3, did not increase in the *skor1b*;*skor2* mutant cerebellum (Fig. S11), indicating that the increased GCs were not due to an increase in the proliferation of GCs. These data suggest that cells in early-stage larvae of *skor1b;skor2* mutants that should have differentiated into PCs instead differentiated into Neurod1^+^ immature GCs. We further examined the expression of *cbln12* and *vglut1*, which have been reported to be expressed in mature GCs (Bae et al., 2009; Kani et al., 2010; Takeuchi et al., 2017), noting that they did not increase at 5 dpf in *skor1b;skor2* mutant larvae (Fig. 9L,P), suggesting that, despite an increase in immature GCs, they did not differentiate into mature GCs. The increase of GCs was no longer evident ectopically at 7 dpf (Fig. 9R, Fig. S12). To examine the ability of Skor to suppress GC differentiation, biotin ligase (BirA, as control) or Skor2, together with mCherry, were expressed in GC progenitors in a mosaic manner using the *Tg(atoh1c:GAL4FF)* line, which expresses a GAL4-VP16 variant in GC progenitors (Kidwell et al., 2018) (Fig. 9S). The expression of Pvalb7 or Neurod1 cells in *atoh1c*^+^-lineage cells expressing transgenes was also examined. When BirA and mCherry was expressed, around 60% of cells were Neurod1^+^ cells (Neurod1^−^ cells are likely undifferentiated GCs; Fig. 9W-Y,AF). In contrast, when Skor2 and mCherry were co-expressed in *atoh1c*^+^ progenitors, the ratio of the Neurod1^+^ population was significantly reduced (Fig. 9AC-AF). No Pvalb7^+^ cells expressed BirA/mCherry or Skor2/mCherry (Fig. 9T-V,Z-AB). These data indicate that Skor2 can inhibit the differentiation of *atoh1c^+^* GC progenitors to Neurod1^+^ GCs, but Skor2 alone cannot induce the differentiation of *atoh1c^+^* cells to PCs.

## DISCUSSION

### Roles of Ptf1a and Neurog1 in the development of cerebellar neural circuits

Whereas *Ptf1a* mutant mice show a complete loss of GABAergic PCs and INs (Hoshino et al., 2005), *ptf1a* mutant zebrafish show only a partial loss of PCs and INs (Itoh et al., 2020) (Fig. 2), suggesting that the contribution of Ptf1a to the development of PCs and INs differs slightly between mice and zebrafish. Here, we have examined the cooperative roles of *ptf1a* and *neurog1* in the development of cerebellar neural circuits. We found that both *ptf1a* and *neurog1* are expressed in the cerebellar VZ in mice and zebrafish (Fig. 1), in agreement with previous studies (Kani et al., 2010; Lundell et al., 2009). Zebrafish *ptf1a*;*neurog1* mutants displayed an almost complete lack of PCs and INs (Fig. 2, Table 1). These data suggest that Ptf1a plays a major role in the development of PCs and INs in zebrafish, whereas Neurog1 functions partially redundantly with Ptf1a in this process. A similar cooperation was observed in the development of crest cells, which were reduced in *ptf1a* mutants and almost absent in *ptf1a;neurog1* mutants (Fig. S2). We previously reported that Ptf1a is essential for the development of IOs in the hindbrain of zebrafish (Itoh et al., 2020). A different dependency of Ptf1a may be explained by overlapping and non-overlapping expression of *ptf1a* and *neurog1* in the rostral (for PC and crest cells) and caudal (for IOs) hindbrain (Fig. 1), as has been reported for mice (Yamada et al., 2007). Lineage tracing revealed that PCs and INs are derived from *ptf1a^+^* neural progenitors (Fig. 4). Considered together, our findings suggest that both PCs and INs in the cerebellum and crest cells in the rostral hindbrain are derived from Ptf1a/Neurog1-expressing neural progenitors in zebrafish.

In addition to PCs and INs, *ptf1a;neurog1* mutants showed reduced expression of *olig2* and *vglut2a* (Fig. 3), which are expressed in ECs in zebrafish cerebellum (Bae et al., 2009; McFarland et al., 2008). Our previous study suggested that *olig2*-expressing ECs are mainly derived from *ptf1a^+^* neural progenitors, but some are derived from *atoh1a^+^* neural progenitors (Kani et al., 2010). Although further lineage tracing of *ptf1a^+^* neural progenitors for ECs is required, the data further support that at least some ECs are derived from Ptf1a/Neurog1-expressing neural progenitors. Furthermore, in *ptf1a;neurog1* mutants, the expression of *atoh1a/b/c* was unaffected at 3 dpf (Fig. S3), but was strongly reduced at 5 dpf (Fig. 3), suggesting that Ptf1a and Neurog1 play a role in maintenance of GC progenitors. It is unclear whether Ptf1a and Neurog1 cell-autonomously or non-cell-autonomously maintain GC progenitors. Whereas in mammals GC progenitors are maintained by Shh produced by PCs (Corrales et al., 2006; Lewis et al., 2004; Wallace, 1999; Wechsler-Reya and Scott, 1999), *shh* is not expressed in PCs and Shh signaling is not activated in the zebrafish cerebellum (Biechl et al., 2016; Chaplin et al., 2010; Hibi et al., 2017). Lineage tracing indicates that at least some GCs were derived from *ptf1a^+^* neural progenitors (Fig. 5). Thus, although not ruling out a non-cell-autonomous function, Ptf1a and Neurog1 likely have a cell-autonomous role in the differentiation of some GCs.

### Does Ptf1a determine GABAergic neural fate?

Loss of function of *Ptf1a* and gain of function of *Ptf1a* and *Atoh1* in mice suggest that Ptf1a and Atoh1 have deterministic roles in the development of GABAergic and glutamatergic neurons, respectively (Hoshino et al., 2005; Pascual et al., 2007; Yamada et al., 2014). However, we found that in zebrafish *ptf1a*^+^ progenitor cells gave rise to GABAergic PCs, INs and GCs (Figs 4 and 5, Fig. S1). It is possible that *ptf1a* and *atoh1* genes are initially co-expressed in the same neural progenitors in the cerebellum, and these cerebellar neurons are derived from the *ptf1a^+^ atoh1^+^* progenitors. However, the PCs, INs and GCs marked in the lineage-tracing experiments were derived from neural progenitors expressing *ptf1a* at 2 dpf (Figs 4 and 5) when the expression regions of *atoh1* genes and *ptf1a* are well separated (Kani et al., 2010). Therefore, at least some of these neurons could be derived from neural progenitors expressing *ptf1a* but not *atoh1* genes. *ptf1a;neurog1* mutants showed an almost complete lack of PCs and INs, but retained GCs at 5 dpf (Figs 2 and 3). Considering that GCs have been reported to be mainly derived from *atoh1*^+^ neural progenitors in early-stage larvae (Kani et al., 2010; Kidwell et al., 2018), GCs derived from *ptf1a^+^* neural progenitors are likely to be a minority among GCs. However, our findings indicate that glutamatergic GCs and possibly ECs can be generated from *ptf1a^+^* neural progenitors, even if in small numbers, in the zebrafish cerebellum.

The data also imply that *ptf1a* expression alone is not sufficient to determine GABAergic neuron fate in the zebrafish cerebellum. How do these zebrafish results align with mouse studies? One possibility is that the regulation of downstream genes that determine cell fates by proneural genes is tight in mice, whereas it is more flexible in zebrafish. The expression of GC deterministic genes, such as *neurod1* (Miyata et al., 1999), may be strictly regulated by ATOH1 in mice, but can be regulated by both Atoh1a/b/c and Ptf1a (and Neurog1) in zebrafish. Further analysis is required to understand how proneural genes control cell fate determination.

### Role of Foxp and Skor family transcriptional regulators in PC differentiation

**Fig. 10.**
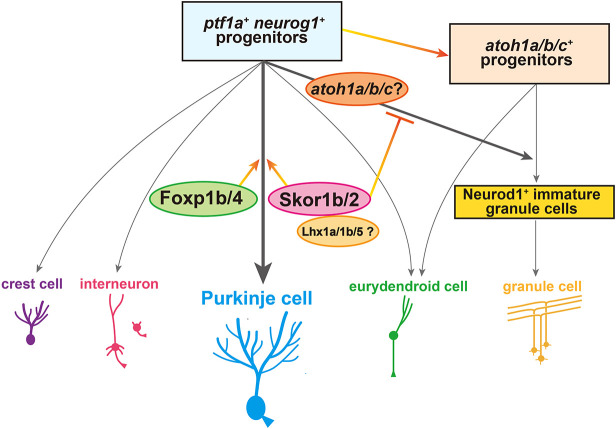
**Schematic of a model for neuronal differentiation from Ptf1a/Neurog1-expressing neural progenitors.** The roles of the transcriptional regulators involved in the specification and differentiation of Purkinje cells from neural progenitors expressing Ptf1a and Neurog1 are described. See Discussion section for more details.

Given that *ptf1a^+^* neural progenitors are capable of generating multiple types of cerebellar neurons, we expect there to be factors that determine the cell fate of each type of neuron. We showed that Foxp and Skor family transcriptional regulators are expressed in PCs, dependent on Ptf1a and Neurog1 (Fig. 2). The *foxp1b;foxp4* mutant showed a strong reduction of PCs (Fig. 7), and *skor1b;skor2* mutants showed the complete loss of PCs (Fig. 8). Furthermore, Foxp1b, Skor1b and Skor2 were expressed in differentiating and differentiated PCs (Fig. 6). These data indicate that Foxp1b/4 and Skor1b/2 function downstream of Ptf1a and Neurog1 as key transcriptional regulators during the initial step of PC differentiation. *skor1b* and *skor2* expression was not affected in *foxp1b;foxp4* mutants (Fig. 7). Although the expression region of *foxp1b* and *foxp4* was affected in *skor1b/skor2* mutants, this may be due to the aberrant differentiation of cerebellar neurons (Fig. 8). Our data suggest that Foxp and Skor family proteins function independently to control PC differentiation (Fig. 10).

Studies of *Foxp2* mutant mice and siRNA-mediated knockdown of *Foxp4* in mice revealed that FOXP2 and FOXP4 function in late developmental processes, such as cell positioning and dendrite formation (Ferland et al., 2003; Tam et al., 2011; Tanabe et al., 2012). In zebrafish, *foxp1b* and *foxp4* are strongly expressed in PCs whereas *foxp1a* and *foxp2* are only slightly expressed in PCs (Takeuchi et al., 2017). Thus, zebrafish *foxp1b* may serve the same function as mouse *Foxp2*. Although *foxp1b;foxp4* showed a strong reduction of PCs, some PCs remained (Fig. 7). It is possible that the function of *foxp1a* or *foxp2* is partially redundantly with that of *foxp1b* and *foxp4* in PC differentiation. Triple or quadruple zebrafish mutants of Foxp family genes should answer this question. *foxp2* is also expressed in IOs in both mice and zebrafish (Fujita and Sugihara, 2012; Itoh et al., 2020). Foxp family proteins may coordinate differentiation from *ptf1a*^+^ neural progenitors to both PCs and IOs, which form the cerebellar neural circuits. It remains elusive whether Foxp proteins function as transcriptional activators or repressors. Previous studies indicated that Foxp1/2/4 can interact with a component of the NuRD remodeling complex, functioning as transcriptional repressors (Chokas et al., 2010). No increased or ectopic expression of GC genes was observed in the cerebellum of *foxp1b;foxp4* mutants, unlike *skor1b;skor2* mutants (Fig. 7). Further analysis is required to understand the molecular mechanisms of Foxp protein-mediated PC differentiation. Foxp1 is involved in many developmental processes, including specification of motor neuron subtypes in the spinal cord (Dasen et al., 2008; Sürmeli et al., 2011). There might be general mechanisms by which Foxp family proteins control specification from neural progenitors to specific types of neurons.

### Mechanisms of Skor1b- and Skor2-mediated control of PC differentiation

Previous studies on the *skor2* mutant suggested that Skor2 is involved in relatively late development of PCs and the suppression of glutamatergic neuronal genes, but it is dispensable for the initial fate specification of PCs (Nakatani et al., 2014; Wang et al., 2011). We demonstrated that *skor1b;skor2* mutants display a complete loss of PCs and instead increase the amount of Neurod1^+^ immature GCs (Figs 8 and 9). Cell proliferation linked to GC proliferation did not increase in *skor1b;skor2* mutants (Fig. S11). Ectopic expression of *skor2* in GC progenitors reduced the expression of Neurod1 (Fig. 9). These data suggest that, in *skor1b;skor2* mutants, cells destined to become PCs differentiated into Neurod1^+^ GCs. Therefore, Skor1b and Skor2 function in the initial step of differentiation from *ptf1a^+^* neural progenitors to suppress differentiation to GCs (Fig. 10). Although Neurod1^+^ GCs increased in *skor1b;skor2* mutants, expression of mature GC markers did not increase in these mutants (Figs 8 and 9), indicating that other factors, which possibly function downstream of Atoh1, are required for differentiation of the Neurod1^+^ immature GCs to mature GCs. Although GCs increased in *skor1b;skor2* mutants at 5 dpf, the increase was not evident at 7 dpf (Fig. 9R, Fig. S12). GCs derived from *ptf1a*^+^ neural progenitors might die. In *ptf1a;neurog1* mutants, although the expression of *skor1b* and *skor2* was absent, we did not observe an increase in *atoh1*^+^ GC progenitors or Neurod1^+^ GCs; instead, there was a decrease (Fig. 3, Table 1). This finding is in contrast with the excess GCs observed in the *skor1b;skor2* mutants (Fig. 9, Table 3). However, this may be due to the absence of *ptf1a*^+^ neural progenitors, which give rise to excess GCs.

It remains elusive whether Skor1b and Skor2 suppress GC fate and thereby secondarily promote PC differentiation, or whether they are also directly involved in PC differentiation independently of GC fate suppression. Mouse SKOR2 has been shown to exhibit transcriptional repression of a reporter in cultured cells (Wang et al., 2011), suggesting that Skor2 can directly repress target genes. Because the direct binding of Skor family proteins to DNA has not been reported, it is likely that their regulation of gene expression requires transcription factor partners that bind to specific elements of DNA. We screened Skor1b/2 interactors by examining co-immunoprecipitation of Skor1/2 with PC-expressing transcription factors from transfected HEK293T cells and found that zebrafish Skor1b and Skor2 can interact with the Lhx family proteins Lhx1a, Lhx1b and Lhx5 (there are two genes for Lhx1 in zebrafish; Fig. S13). *lhx1a* and *lhx1b* were expressed in the cerebellum of early-stage larvae (Fig. S5). We generated zebrafish crispants and stable mutants of *lhx1a*, *lhx1b* and *lhx5* (Figs S14, S15, S16). Similar to *Lhx1;Lhx5* mutant mice (Zhao et al., 2007), we found that *lhx1a;lhx5* zebrafish crispants/mutants showed a severe reduction of PCs and *lhx1a;lhx1b;lhx5* zebrafish crispants/mutants showed a more pronounced reduction or complete loss of PCs (Figs S15, S16, Table S1), as did s*kor1b;skor2* mutants. Although Lhx proteins are thought to function as transcriptional activators (Hobert and Westphal, 2000), they may also function with Skor proteins as repressors to repress the expression of GC genes. Alternatively, Skor1b and Skor2 cooperate with Lhx-family proteins to positively promote the expression of some PC genes. The identification of target genes of Skor1b/2 and Lhx1a/1b/5 by chromatin immunoprecipitation should clarify this issue. In any case, Skor and Lhx family transcriptional regulators might cooperate to induce PC differentiation and/or suppress GC fate (Fig. 10).

### Gene networks for PC differentiation

In this study, we demonstrate that there are two steps to determine whether cells become PCs or GCs in the cerebellum. In the first step, expression of proneural genes roughly determine cell fate: expression of *atoh1* induces differentiation into GCs, whereas *ptf1a* expression induces the differentiation of PCs. However, expression of proneural genes is not sufficient to determine cell fate. In the second step, Skor family proteins act as gatekeepers to prevent cells from becoming GCs. Foxp, Skor and Lhx family proteins cooperate to promote PC differentiation. This two-step control of PC differentiation ensures that an appropriate number of PCs and GCs are generated to form functional cerebellar neural circuits. Among *ptf1a^+^* neural progenitors, *foxp1b/4* and *skor1b/2* are only expressed in cells that differentiate into PCs, but not INs, ECs or GCs. We expect there to be upstream regulators that restrict their expression only to PCs. Studies of factors that function upstream and downstream of Foxp and Skor family genes will provide an understanding of gene networks that control the differentiation of PCs and other cerebellar neurons.

## MATERIALS AND METHODS

### Zebrafish strains and genes

The animal work in this study was approved by the Nagoya University Animal Experiment Committee and was conducted in accordance with the Regulations on Animal Experiments at Nagoya University. WT zebrafish with the Oregon AB genetic background were used. For immunohistochemistry and whole-mount *in situ* hybridization, larvae were treated with 0.003% 1-phenyl-2-thiourea (PTU) (Nacalai-Tesque, 27429-22) to inhibit the formation of pigmentation. Zebrafish mutant *ptf1a*^Δ*4*^ (*ptf1a^nub34^*) and *neurog1^hi1059Tg^* were described previously (Golling et al., 2002; Itoh et al., 2020). Transgenic zebrafish *Tg(ptf1a:EGFP)jh1Tg* (Pisharath et al., 2007), *TgBAC(ptf1a:GAL4-VP16)jh16Tg* (Parsons et al., 2009), *Tg(UAS:RFP)nkuasrfp1aTg* (Asakawa et al., 2008), *TgBAC(neurog1:GFP)nns27Tg* (Satou et al., 2013), *TgBAC(atoh1c:GAL4FF)fh430Tg* (Kidwell et al., 2018), *TgBAC(gad1b:LOXP-DsRed-LOXP-GFP)nns26Tg* and *TgBAC(slc17ab:LOXP-DsRed-LOXP-GFP)* (Satou et al., 2013) were also described previously. The allele names of the *ptf1a^Tg(hsp70l-EGFP)^*, *foxp1b*^Δ*26*^, *foxp4*^Δ*7*^, *skor1b*^Δ*10*^, *skor2*^Δ*8*^, *lhx1a*^Δ*10*^, *lhx1b*^Δ*17*^ and *lhx5*^Δ*10*^ lines established in this study are designated as *ptf1a^nub121Tg^*, *foxp1b^nub89^*, *foxp4^nub90^*, *skor1b^nub91^*, *skor2^nub92^*, *lhx1a^nub93^*, *lhx1b^nub94^* and *lhx5^nub95^* respectively, in ZFIN (https://zfin.org). The open reading frame of *foxp1b*, *foxp4*, *skor1b* and *skor2* mRNAs were isolated by RT-PCR and their sequence information was deposited in DDBJ with the accession numbers LC760469, LC760470, LC760471 and LC760472, respectively. The *skor2* mRNA sequence in a public database (NM_001045421) lacked a region encoding the carboxy-terminal region, so the full open reading frame of *skor2* was isolated in this study. Zebrafish were maintained at 28°C under a 14-h light and 10-h dark cycle. Embryos and larvae were maintained in embryonic medium (EM) (Westerfield, 2000).

### Establishment of transgenic zebrafish

To establish *Tg(5xUAS-hsp70l:mCherry-T2A-CreERT2)* fish, pENTR L1-R5 entry vector containing five repeats of the upstream activation sequence (UAS) and the *hsp70l* promoter (*5xUAS-hsp70l*) (Muto et al., 2017), and pENTR L5-L2 vector containing mCherry cDNA, the 2A peptide sequence of *Thosea asigna* virus (TaV), CreERT2 recombinase cDNA (Ukita et al., 2009), and the SV40 polyadenylation signal (SV40pAS) from pCS2+ were subcloned to pDon122-Dest-RfaF, which was derived from a Tol1 donor plasmid (Koga et al., 2008, 2007), by the LR reaction of the Gateway system. To generate *Tg(cbln12:LOXP-TagCFP-LOXP-Kaede)* fish, the TagCFP DNA fragment was amplified from pTagCFP-N (Evrogen) by PCR with the primers 5′-GAAGATCTATAACTTCGTATAGCATACATTATACGAAGTTATACCGGTCGCCACCATGAGCG-3′ and 5′-CCGGAATTCCGGATCCATAACTTCGTATAATGTATGCTATACGAAGTTATACCACAACTAGAATGCAGTG-3′, and subcloned to BamHI and EcoRI sites of pCS2+ after digestion with BglII and EcoRI (pCS2+lTl). Kaede cDNA from pCS2+Kaede was inserted to BamHI and XbaI sites of pCS2+lTl-Kaede, which contains SV40pAS. The 2-kpb *cbln12* promoter (Dohaku et al., 2019) and lTl-Kaede-pAS were subcloned to pT2ALR-Dest by NEBuilder (NEB, E2621L). To generate *Tg(5xUAS-hsp70l:HA-skor2-P2A-mCherry, myl7:mCherry)*, 3xHA (influenza hemagglutinin)-tagged *skor2* cDNAs, the 2A peptide sequence from porcine teschovirus-1 (PTV1), and mCherry cDNA were subcloned into pCS2+, and transferred to the pENTR L5-L2 vector by the BP reaction of the Gateway system. The pENTR L1-R5 plasmid containing *5xUAS-hsp70l* and pENTR L5-L2 containing the *skor2* expression cassette were subcloned into pBleeding Heart (pBH)-R1-R2 (Dohaku et al., 2019), which contains mCherry cDNA and SV40pAS under control of the *myosin*, *light chain 7*, *regulatory* (*myl7*) promoter. To generate *Tg(5xUAS-hsp70l:BirA-P2A-mCherry, myl7:mCherry)*, pENTR L1-L5 plasmid which contains *5xUAS-hsp70l*, and pENTR L5-L2, which contains BirA cDNA (Matsuda et al., 2017), the 2A peptide sequence from PTV1, and mCherry cDNA were subcloned into pBH-R1-R2. To make transgenic fish, 25 pg of Tol2 plasmid DNA and 25 pg of Tol2 transposase RNA, or 20 pg of Tol1 plasmid DNA and 80 pg of Tol1 transposase RNA were injected into one-cell-stage WT embryos. The allele names of the Tg lines established in this study were designated as *Tg(5xUAS-hsp70l:mCherry-T2A-CreERT2)nub99Tg*, *Tg(cbln12:LOXP-TagCFP-LOXP-Kaede)nub96Tg*, *Tg(5xUAS-hsp70l:HA-skor2-P2A-mCherry*, *myl7:mCherry)nub97Tg*, and *Tg(5xUAS-hsp70l:BirA-P2A-mCherry, myl7:mCherry)nub122Tg* in ZFIN.

### Establishment of zebrafish knockout and knock-in mutants using the CRISPR/Cas9 system

gRNA targets were designed using the web software ZiFiT Targeter and CRISPRscan (Hwang et al., 2013; Mali et al., 2013; Moreno-Mateos et al., 2015). To generate gRNAs, the following oligonucleotides were used: 5′-TAGGCCGGTGTTCAGAGCACAG-3′ and 5′-AAACCTGTGCTCTGAACACCGG-3′ for *foxp4*^Δ*7*^; 5′-TAGGAGATCCTCAGGCCGCGG-3′ and 5′-AAACCCGCGGCCTGAGGATCT-3′ for *skor1b*^Δ*10*^; 5′-TAGGTTATCATGCCACAGCGC-3′ and 5′-AAACGCGCTGTGGCATGATAA-3′ for *skor2*^Δ*8*^; 5′-TAGGAAGAGGCGGAGGCGCATG-3′ and 5′-AAACCATGCGCCTCCGCCTCTT-3′ for *ptf1a^Tg(hsp70l-EGFP)^*, which was previously used to generate the *ptf1a*^Δ*4*^ mutant (Itoh et al., 2020). gRNA and Cas9 mRNA syntheses were performed as previously reported (Nimura et al., 2019). A solution containing 25 ng/μl gRNA and 100 ng/μl Cas9 mRNA or 1000 ng/μl Cas9 protein (ToolGen Inc.) was injected into one-cell-stage embryos using a pneumatic microinjector (PV830, WPI). The knock-in line *ptf1a^Tg(hsp70l-EGFP)^* was generated as previously described (Kimura et al., 2014). To establish the *foxp1b* mutant, chemically synthesized crRNAs and tracrRNAs (Fasmac) were used. The following target sequence was selected: 5′-TGGCGTGAGAGGGGCCGTTG-3′. To establish *lhx1a*, *lhx1b* and *lhx5* mutants and *foxp1b*, *foxp4*, *skor1b*, *skor2* crispants (F0 mutants), chemically synthesized Alt-R^®^ crRNAs and tracrRNAs, and Cas9 protein (Integrated DNA Technologies) were used. The following target sequences were selected: 5′-GCGAGAGGCCTATATTGGACAGG-3′ for *lhx1a*; 5′-TGAGCGTCTTGGACAGAGCCTGG-3′ for *lhx1b*; 5′-GTGAGAGGCCCATTCTGGATCGG-3′ for *lhx5*; 5′-ACGGTCACGGCGTCTGCAAA-3′ for *foxp1b*; 5′-GATCTGAGGTGAGACCTTGG-3′ for *foxp4*; 5′-CGGGATGATTACAAAGCGAG-3′ for *skor1b*; and 5′-CCACAACCGTCGAGTAGCTC-3′ for *skor2*. To prepare the crRNA:tracrRNA Duplex and gRNA, Cas9 RNP complexes were established as previously reported (Hoshijima et al., 2019). To generate crispants, a solution was prepared containing 5 μM crRNA, 5 μM tracrRNA and 5 μM Cas9 proteins for *skor1b*, *skor2*, *lhx1a*, *lhx1b* and *lhx5*. For *foxp1b* and *foxp4*, the solution contained 10 μM crRNA, 10 μM tracrRNA and 10 μM Cas9 protein. One nanoliter of the respective solution was injected into one-cell-stage embryos. Mutations in the target region were detected by a heteroduplex mobility assay (Ota et al., 2013) and confirmed by sequencing after subcloning the target regions amplified from the mutant genome into pTAC-2 (BioDynamics Laboratory, DS126).

### Genotyping

To detect mutations, the following primers were used: 5′-CCCCTCAGTTTACCCCAGA-3′ and 5′-TGAGTAGCGTCTGCGTATGG-3′ (*foxp1b*^Δ*26*^); 5′-CTAGGTCGACGCTGGATGAT-3′ and 5′-CGACTGAAAATCTTCAAACACAG-3′ (*foxp1b* crispants); 5′-TGTTTTAGCCATGTGTCCCACTGA-3′ and 5′-GCTGTTGGTGGTCAGATCGA-3′ (*foxp4*^Δ*7*^); 5′-CTCGATCTGACCACCAACAG-3′ and 5′-GCTCATGCATTTTCCACTGA-3′ (*foxp4* crispants); 5′-CCTCTCGGCCTCTCGCTTTGTA-3′ and 5′-CTGGGCATCACCTGTGTGCA-3′ (*skor1b*^Δ*10*^); 5′-TATGCCCATTTCCTCGAGAC-3′ and 5′-TCAAAAGCGAAATTTTCTGG-3′ (*skor1b* crispants) 5′-AGACATTGTGATGGCAACCCCA-3′ and 5′-CGTAGAGGATGACCTGCCCA-3′ (*skor2*^Δ*8*^); 5′-CCTGGCTCAGATATCCAACA-3′ and 5′-GGATCTCAAGCTGGACTGGA-3′ (*skor2* crispants) 5′-GGAGCACATCCAAAGACGAT-3′ and 5′-CTTGATGTGCCATGCTCTGT-3′ (*lhx1a*^Δ*10*^); 5′-CAAAACATGGTCCACTGTGC-3′ and 5′-TGCATTTACAGTCACAGCATTG-3′ (*lhx1b*^Δ*17*^); 5′-CGGAATGATGGTGCACTG-3′ and 5′-GTTACACTCGCAGCATTGGA-3′ (*lhx5*^Δ*10*^). To detect the *neurog1^hi1059Tg^* mutation, which is induced by retrovirus insertion, the following three primers were used: 5′-AAAGAAAAGTGGTGGGAAAGCC-3′ as the forward primer annealing to the genomic region adjacent to the 5′ portion of the retrovirus; 5′-TCGCTTCTCGCTTCTGTTCG-3′ as the reverse primer annealing to the 3′ portion of the retrovirus; and 5′- GCACAACGTTAGGTATTCACTGTTTG-3′ as another reverse primer annealing to the genomic region adjacent to the 3′ portion of the retrovirus. The WT and *neurog1^hi1059Tg^* mutant alleles gave rise to 412 and 300 bp DNA fragments, respectively.

### Treatment with endoxifen

A 4 μM solution of endoxifen was prepared by adding 0.96 μl of 25 mM endoxifen (Sigma-Aldrich, SML2368) dissolved in DMSO into 6 ml of E3 medium (5 mM NaCl, 0.17 mM KCl, 0.4 mM CaCl_2_ and 0.16 mM MgSO_4_) containing 0.004% PTU. To induce CreERT2-mediated recombination, 2 dpf larvae were treated with the endoxifen solution for 16 h. After washing with E3/PTU medium, larvae were cultivated in this medium until 5 dpf. For the control, DMSO was used instead of 25 mM endoxifen DMSO stock.

### *In situ* hybridization

Whole-mount *in situ* hybridization was performed as previously reported (Bae et al., 2009). Detection of *ptf1a* and *neurog1* was previously described (Bae et al., 2005; Kani et al., 2010). Larvae were hybridized with digoxigenin (DIG)-labeled riboprobes overnight at 65°C and incubated overnight with 1/2000 alkaline phosphatase-conjugated anti-DIG Fab fragment (Roche, 11093274910) at 4°C. BM Purple AP substrate (Roche, 11442074001) was used as the alkaline phosphatase substrate. Images were acquired using an Axioplan 2 microscope equipped with an AxioCam CCD camera (Zeiss).

### Generation of antibodies and immunohistochemistry

Polyclonal antibodies against Foxp1b, Skor1b and Skor2 were generated by immunizing rabbits with the synthetic peptides CHRDYEDDHGTEDML, MESIPNQLPAGRDSSC and CIPYANIIRKEKVGTHLNKS (the underlined C was added to link the peptides covalently with keyhole limpet hemocyanin), respectively. These antibodies were purified using peptide affinity columns that were generated from vinyl polymer resin (TOSOH Bioscience, TOYOPearL AF-Amino-650) and crosslinker m-maleimidobenzoyl-N-hydroxysuccinimide ester (MBS, Thermo Fisher Scientific, 22311). For validation information, see the supplementary Materials and Methods. For immunostaining, anti-parvalbumin 7 (1/1000, mouse monoclonal ascites), anti-carboxy anhydrase 8 (1/100, mouse monoclonal, hybridoma supernatant) (Bae et al., 2009), anti-Zebrin II (1/200, mouse monoclonal hybridoma supernatant) (Lannoo et al., 1991), anti-Vglut1 (1/250, rabbit polyclonal) (Bae et al., 2009), anti-Neurod1 (1/500, mouse monoclonal, hybridoma) (Kani et al., 2010), anti-paired box 2 (1/700, rabbit polyclonal) (BioLegend, 901001), and the in-house-generated anti-Foxp1b, anti-Skor1b and anti-Skor2 (1/1000, rabbit polyclonal, affinity purified) antibodies were used. CF488A goat anti-mouse IgG (H+L, Biotium, 20018-1), CF488A goat anti-rabbit IgG (H+L, Biotium, 20019), CF568 goat anti-mouse IgG (H+L, Biotium, 20301-1) and CF568 goat anti-rabbit IgG (H+L, Biotium, 20103) were used as the secondary antibodies. Larvae and cryosections were immunostained as described previously (Bae et al., 2005; Itoh et al., 2020; Kani et al., 2010). For Skor1b and Skor2 immunostaining, larvae were fixed and treated with acetone at 4°C instead of −30°C. A Zeiss LSM700 confocal laser-scanning microscope was used to obtain fluorescence images. Images were acquired under nearly identical conditions. To show individual cells, confocal optical sections were used (Fig. 6). In Fig. 9T-AE, the dynamic range of fluorescence intensity was modified to compensate for differences in the expression of fluorescent proteins and staining conditions.

### Quantification of image data

To quantify *in situ* hybridization and immunohistochemistry data, with some exceptions, image data were imported into image processing software ImageJ (https://imagej.net/ij/) and Fiji (https://fiji.sc), and the cerebellar region was cropped from the image. Binarization was performed after manually setting an arbitrary threshold. The areas with signals were measured. The average area of the data obtained for each group was calculated, and it was compared with the data from WT. Ratings were given based on comparison with WT data: +++, ++, + or − for values of ≥0.8, 0.5-0.8, 0.01-0.5, and <0.01, respectively. In some cases, only a subset of the larvae showing an expression phenotype were qualitatively assessed. Because of high background staining, the expression of *vglut2a* in *ptf1a;neurog1*, *foxp1b;foxp4* and *skor1b;skor2* mutants, as well as the expression of *foxp4* and *reln* in *skor1b;skor2* mutants, was qualitatively assessed and rated.

### Cell transfection, immunoprecipitation and immunoblotting

cDNAs encoding carboxy-terminally 3× hemagglutinin epitope-tagged Skor1b or Skor2 (Skor1b-3xHA, Skor2-3xHA), amino-terminally 6× Myc epitope-tagged Skor1b or Skor2 (6xMT-Skor1b, 6xMT-Skor2), and amino-terminally 3× Flag epitope-tagged Lhx1a, Lhx1b, or Lhx5 were inserted to pCS2+. HEK293T cells in 6 cm dishes were transfected with 2 μg Skor expression plasmid DNA, 2 μg Lhx expression plasmid DNA, and 1 μg pCS2+Venus in the indicated combination using HilyMax (DOJINDO Laboratories, H357). For the control, 2 μg pCS2+ was added to bring the total amount to 5 μg. Cells were lysed, 24 h after transfection, in 1 ml lysis buffer (10 mM Tris pH 7.4, 150 mM NaCl_2_, 0.5% NP40) containing protease inhibitor cocktail (Nacalai-Tesque, 25955) and cleared by centrifugation (15,300 ***g*** for 20 min). For immunoprecipitation, 1 μg antibody was bound to 10 μl Dynabeads protein G (Thermo Fisher Scientific, Invitrogen, 10003D) for 20 min at room temperature and washed with lysis buffer. Then, 500 μl of cell lysates were mixed with antibody-bound protein G beads and incubated at 4°C for 2 h with rotation. Antibody-bound fractions were collected by magnetic beads, washed with lysis buffer five times, and eluted with 20 μl 2× SDS-polyacrylamide electrophoresis sample buffer. Immunoprecipitated samples were separated on a polyacrylamide gel (SuperSep, Wako, 194-15021, 197-15011) and transferred to a PVDF membrane (Immobilon-P, Millipore, IPVH00010). After blocking with 3% skimmed milk in TBS-T (20 mM Tris pH 7.4, 150 mM NaCl, 0.1% Tween 20), the membranes were immunoblotted with CanGet Signal (TOYOBO, NK101) and Chemi lumi One L (Nacalai, 07880-54). The antibodies used were: anti-HA mouse IgG1-κ (HA124, Nacalai, 06340-54), anti-c-Myc mouse IgG1-κ (9E10, Santa Cruz Biotechnology, sc-40) and anti-Flag mouse IgG1 (M1, Sigma-Aldrich, F3165). Images were captured by a CCD camera Lumiviewer.

### Statistics

Data were analyzed using GraphPad Prism (version 5.1 and 6.0) or R software package (version 4.2.2).

## Supplementary Material



10.1242/develop.202546_sup1 Supplementary information

Table S2. Source data of Table 1, 2, 3, and S1
