# Supplementary material for: Foxp and Skor family proteins control differentiation of Purkinje cells from Ptf1a- and Neurog1-expressing progenitors in zebrafish
Source: Development. 2024 Apr 2;151(7):dev202546. doi: 10.1242/dev.202546 (PMC11057878; doi:10.1242/dev.202546)
Supplement: Supplementary information [file develop-151-202546-s1.pdf]

Supplementary Materials and Methods

Validation of antibodies

Anti-Foxp1b, Skor1b and Skor2 antibodies were validated by immunoprecipitation, immunoblotting and immunostaining. HEK293T cells were transfected with expression plasmids for Myc-tagged Foxp1b, HA-tagged Skor1 or HA-tagged Skor2. We examined whether the antibodies could immunoprecipitate tagged target proteins or detect tagged proteins by immunoblotting. Furthermore, immunostaining of wild-type and mutant larvae deficient in these genes was performed to exclude the possibility that the antibodies produced non-specific immunostaining signals in the mutants.

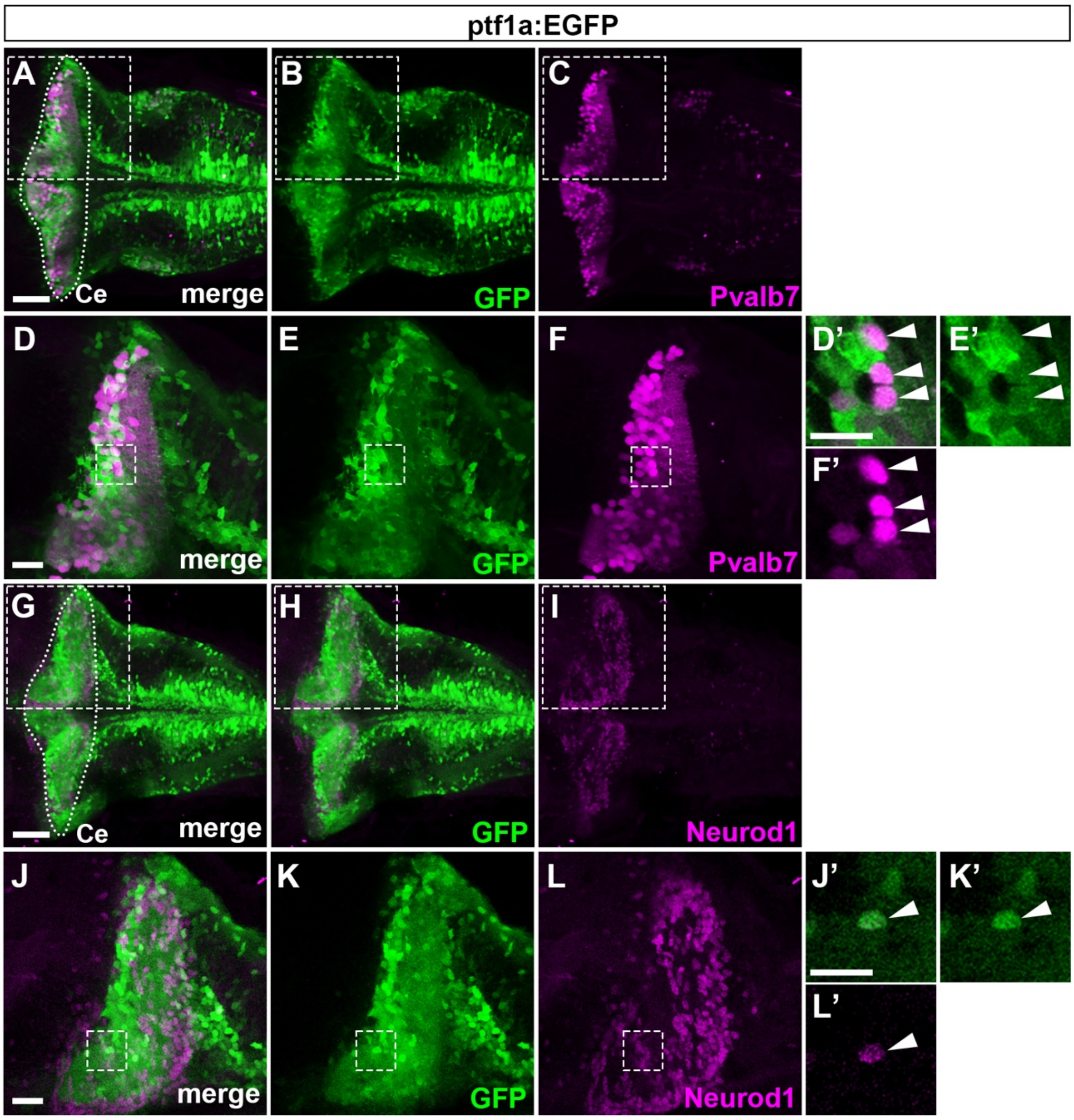

Fig. S1. Expression of GFP in *TgBAC(ptf1a:GFP)*.

5-dpf *TgBAC(ptf1a:GFP)* larvae were stained with anti-GFP, and anti-Pvalb7 (n=3, A-F) or Neurod1 (n=2, G-L) antibodies. (D'-F', J'-L') Higher magnification views of boxes in (D-F, J-L). Dorsal views with anterior to the left. The cerebellum region (Ce) is surrounded by a dotted line. Many ptf1a:GFP$^+$ cells were co-stained with Pvalb7 (D, arrowheads in D'-F') and a few ptf1a:GFP$^+$ cells were co-stained with Neurod1 (J, arrowhead in J'-L'). In one half of the cerebellum, one larva had 14 GFP$^+$ cells out of 225 Neurod1$^+$ cells, the other had 14 GFP$^+$ cells out of 206 Neurod1$^+$ cells. Scale bars: 50 µm in A (applies to A-C); 20 µm in D (applies to D-F); 50 µm in G (applies to G-I); 20 µm in J (applies to J-L); 10 µm in D' (applies to D'-F'); 10 µm in J' (applies to J'-L').

Development • Supplementary information

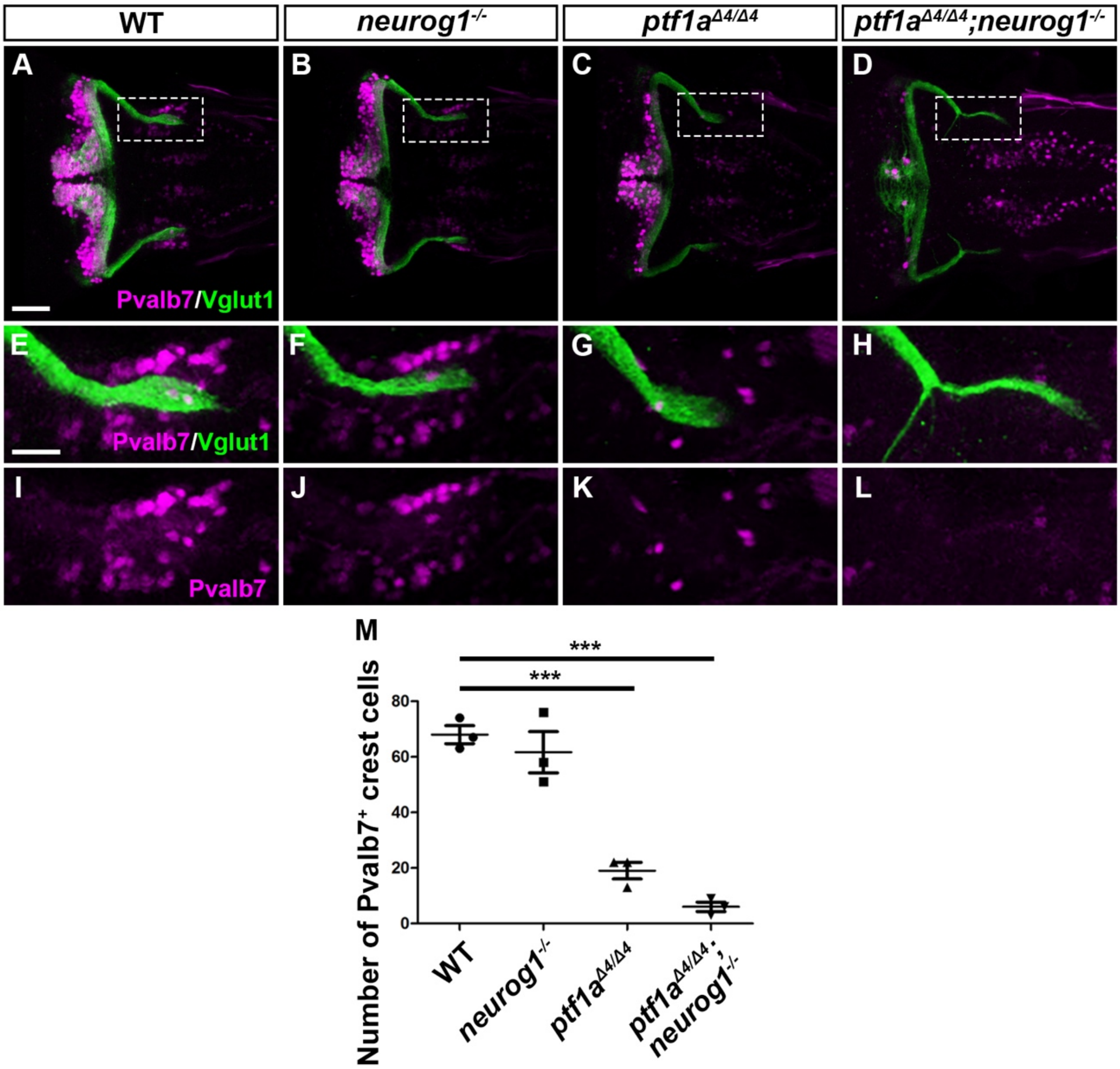

Fig. S2. Crest cells in *neurog1*, *ptf1a*, *ptf1a;neurog1* mutants.
5-dpf wild-type (WT), *neurog1*, *ptf1a*, and *ptf1a;neurog1* mutant larvae were immunostained with anti-Pvalb7 (magenta) and Vglut1 (green) antibodies. Dorsal views with anterior to the left. (E-H) Higher magnification views of boxes in A-D. (I-L) Only Pvalb7 expression in E-H is shown. Scale bars: 50 µm in A (applies to A-D); 20 µm in E (applies to E-L). (M) Number of Pvalb7$^+$ crest cells in 5-dpf WT, *neurog1*, *ptf1a*, and *ptf1a;neurog1* mutant larvae. $^{***}P$<0.001 (ANOVA with Tukey's multiple comparison test). Data are means±SE with individual values indicated.

Development • Supplementary information

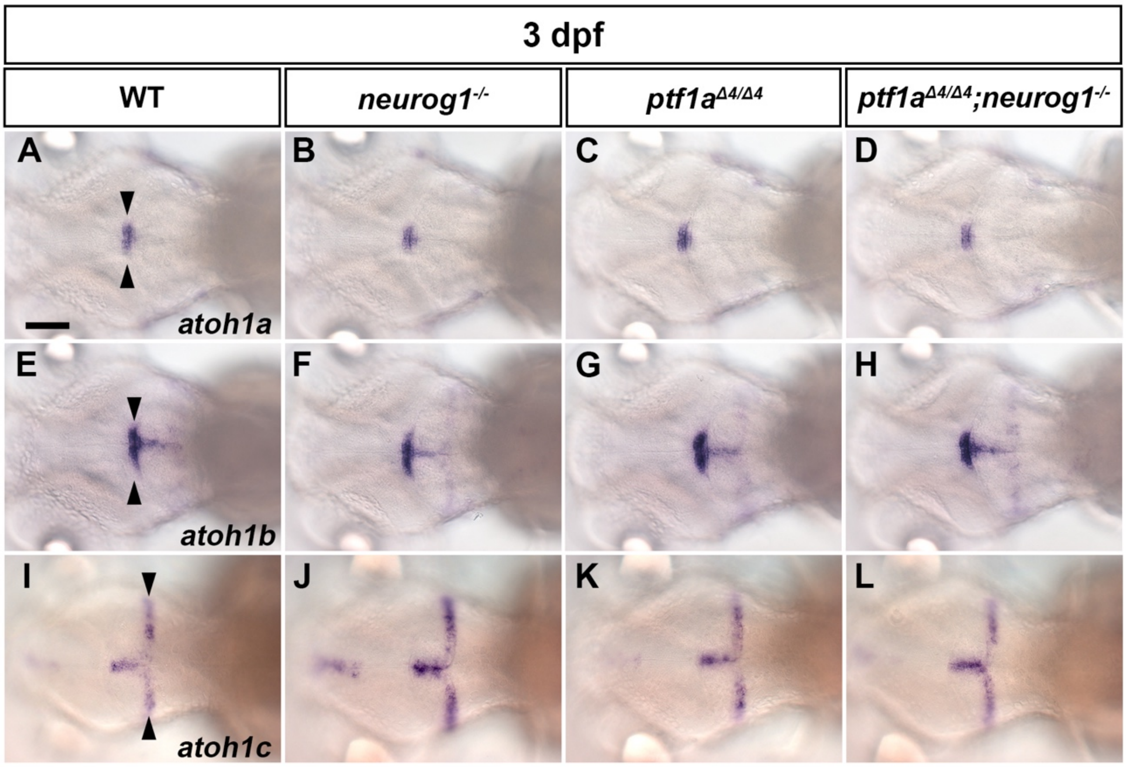

Fig. S3. Expression of *atoh1* genes in *neurog1*, *ptf1a*, *ptf1a; neurog1* mutants. Expression of *atoh1a*, *atoh1b*, and *atoh1c* in 3-dpf WT, *neurog1*, *ptf1a*, and *ptf1a; neurog1* mutant larvae. Data of *in situ* hybridization. Dorsal views with anterior to the left. The number of examined larvae is shown in Table 1. Scale bar: 100 µm in A (applies to all panels).

Development • Supplementary information

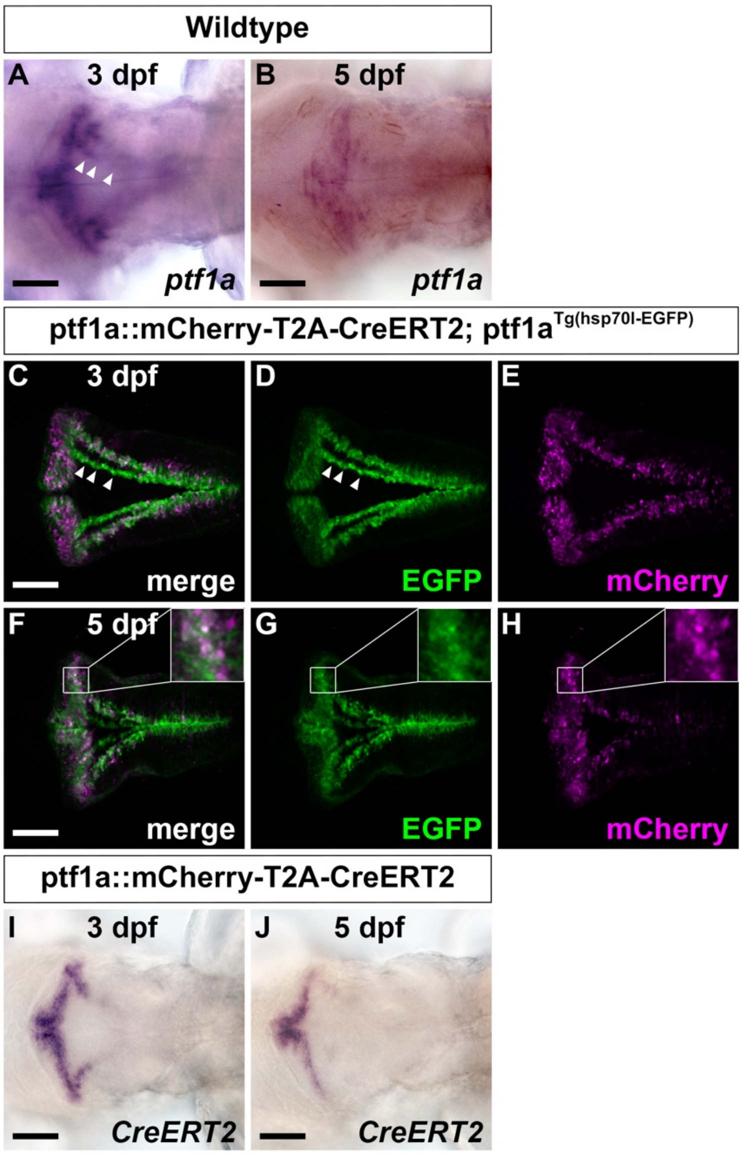

Fig. S4. CreERT2 expression in *ptf1a*-expressing neural progenitors in the lineage-tracing line.

(A, B) *ptf1a* expression at 3 and 5 dpf. (C-H) mCherry expression (magenta) in *TgBAC(ptf1a:Gal4-VP16);Tg(UAS-hsp70l:mCherry-T2A-CreERT2)* and EGFP expression (green) in *ptf1a*$^{Tg(hsp70l\text{-}EGFP)}$ larvae at 3 and 5 dpf. The insets of F-H provide a higher magnification view of the boxed area in the corresponding figures. (I, J) *CreERT2* expression at 3 and 5 dpf. Note that EGFP expression in *ptf1a*$^{Tg(hsp70l\text{-}EGFP)}$ larvae recapitulated *ptf1a* expression. mCherry was expressed in EGFP-expressing cells except those located medially (ventrally) in the hindbrain (marked by arrowheads) and recapitulated CreERT2 expression. Scale bars: 100 µm in A; B; C (applies to C-E); F (applies to F-H); I; J.

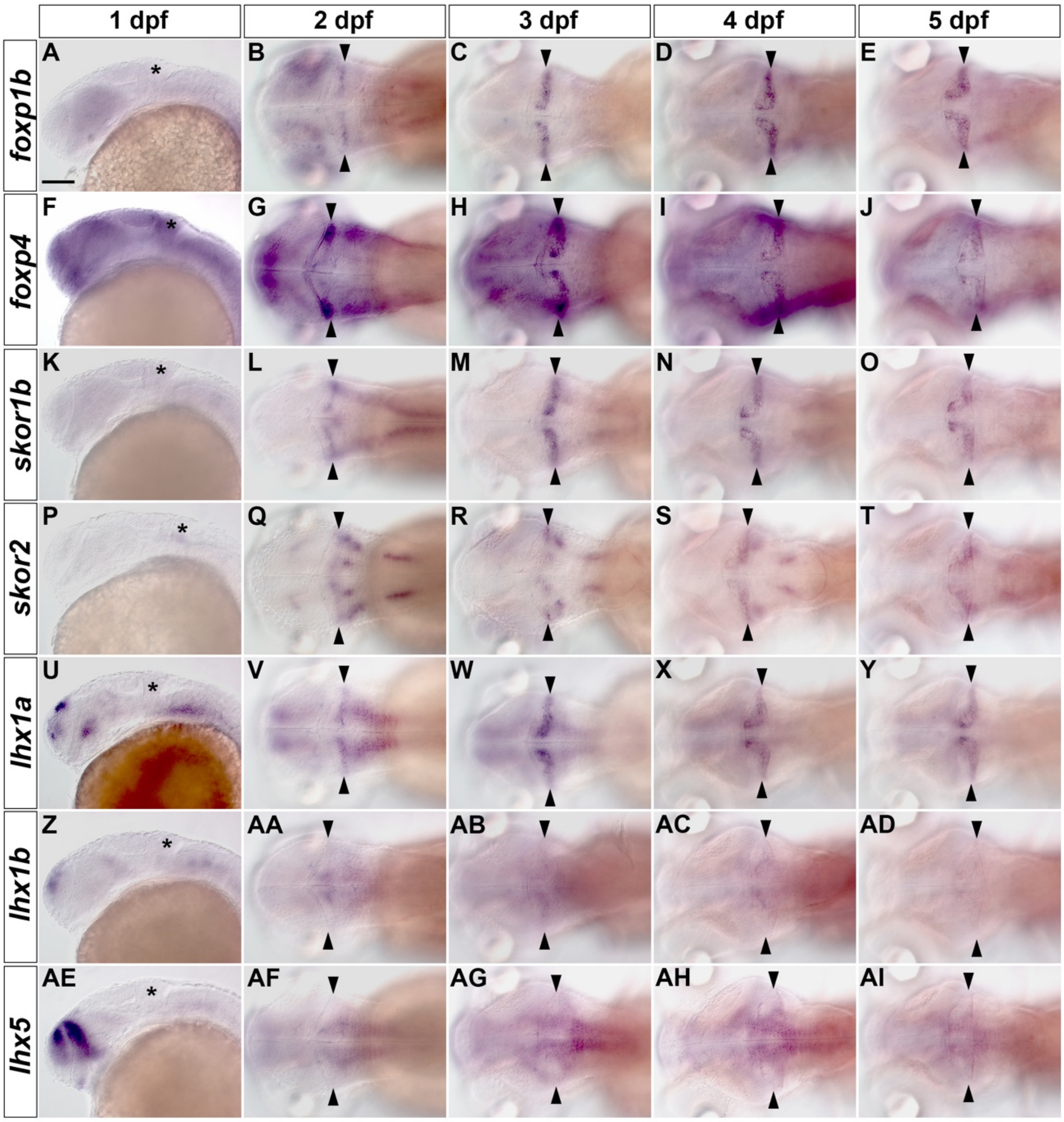

Fig. S5. Expression of *foxp*, *skor*, and *lhx*-family genes during development. Expression of *foxp1b* (A-E), *foxp4* (F-J), *skor1b* (K-O), *skor2* (P-T), *lhx1a* (U-Y), *lhx1b* (Z-AD), and *lhx5* (AE-AI) in the cerebellum region at 1, 2, 3, 4, and 5 dpf. Lateral views with anterior to the left (A, F, K, P, U, Z, AE). Dorsal views with anterior to the left (B-E, G-J, L-O, Q-T, V-Y, AA-AD, AF-AI). The cerebellum region is marked by asterisks or arrowheads. Scale bar: 100 µm in A (applies to all panels).

Development • Supplementary information

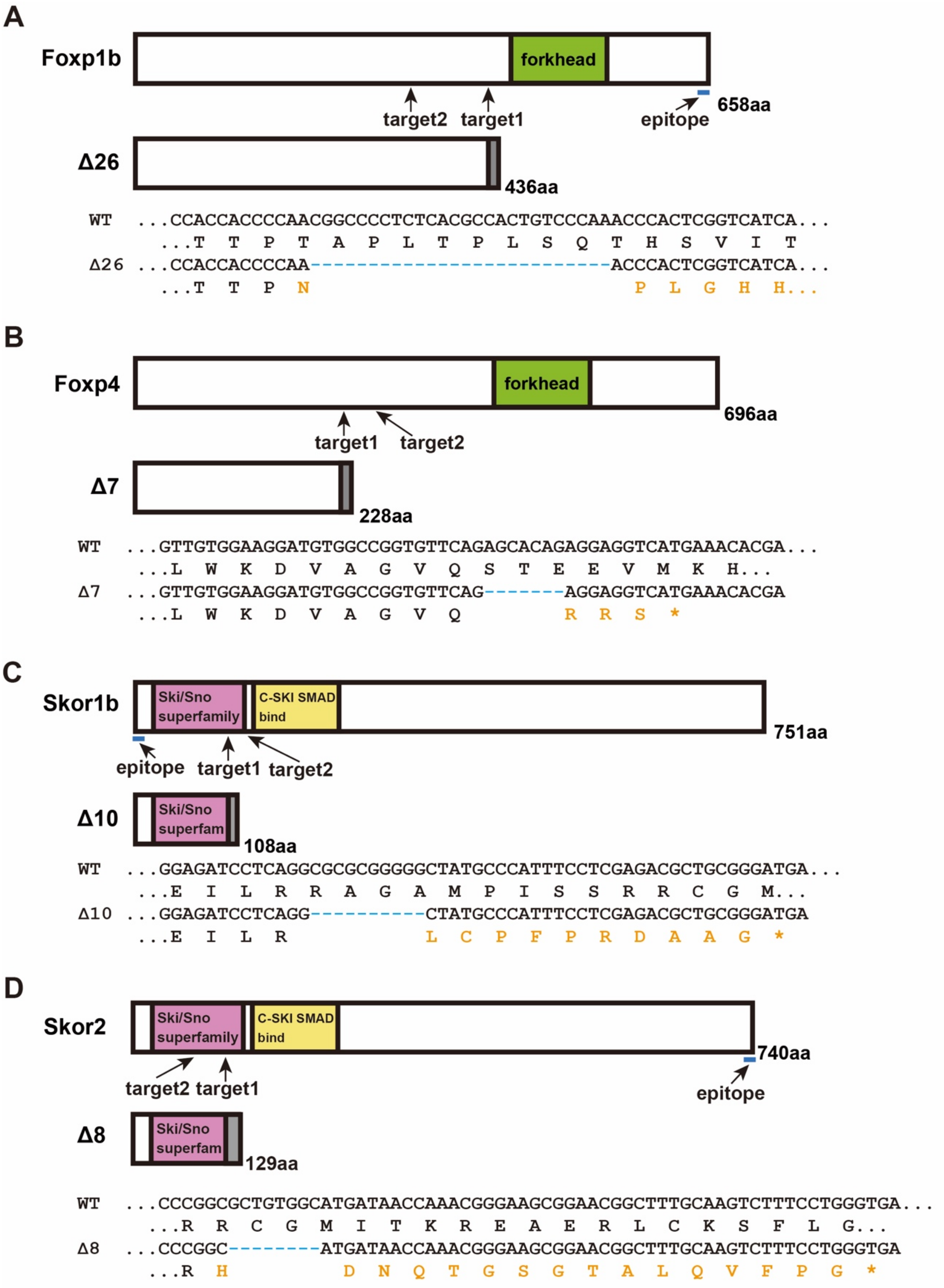

Fig. S6. Structure of wild-type (WT) and mutant Foxp1b, Foxp4, Skor1b, and Skor2.

Development • Supplementary information

Structure of WT and mutant Foxp1b (A), Foxp4 (B), Skor1b (C), and Skor2 (D), and nature of mutations generated by the CRISPR/Cas9 method. The positions of the CRISPR/Cas9 targets are shown. Target 1 is the target when creating stable mutants, and target 2 is the target when creating crispants. The deletion is marked in blue. The deletion mutations in these genes cause a frameshift, the addition of unrelated amino acids (marked in gray), and a premature stop codon. The mutation of *foxp1b*, *foxp4*, *skor1b* and *skor2* results in the addition of 42, 3, 10, and 16 unrelated amino acids, respectively (marked in orange). All of the putative mutant proteins lack the functional domain(s) conserved among the Foxp- or Skor-family proteins. Foxp1b and Foxp4 have a forkhead domain. Skor1b and Skor2 have a Ski/Sno superfamily domain and a c-SKI SMAD binding domain, respectively. The positions of the epitope used as the antigen for the antibodies produced in this study are also indicated.

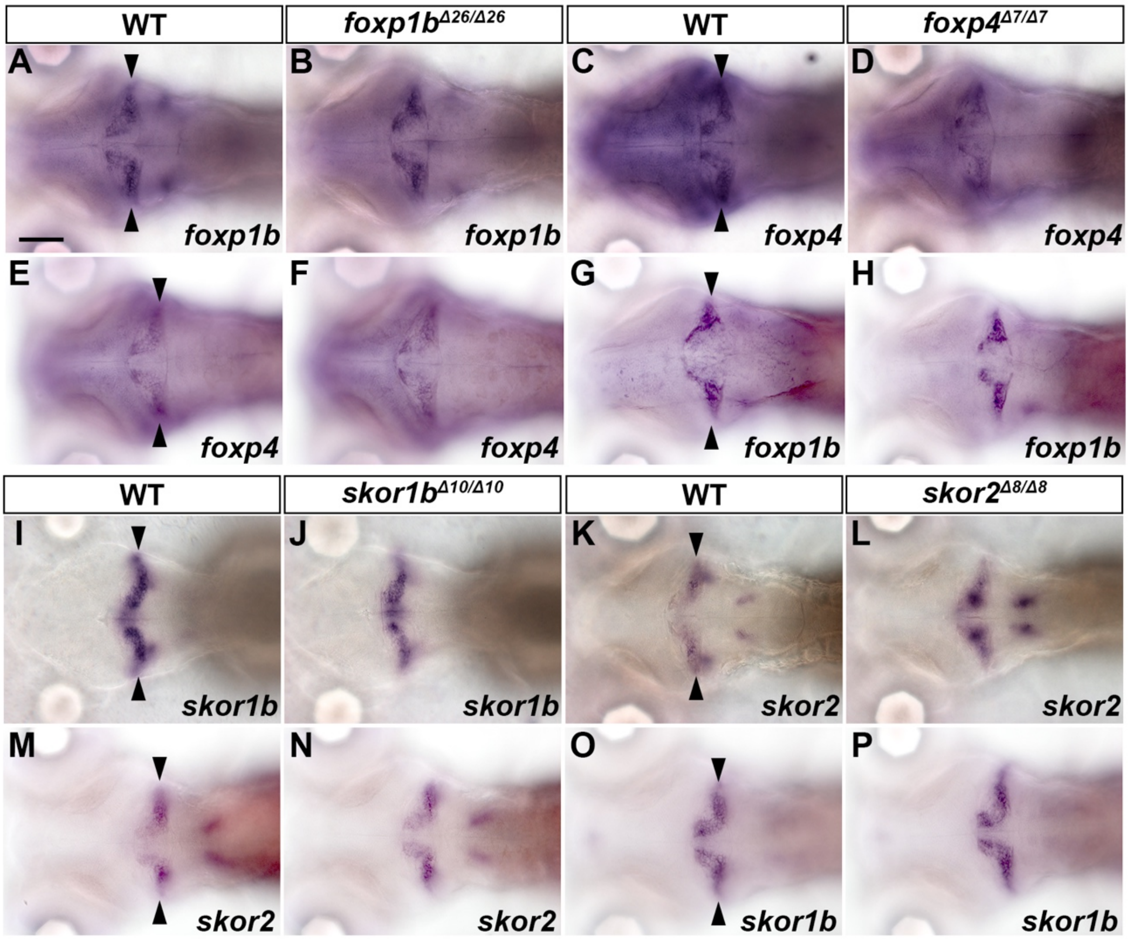

Fig. S7. Expression of *foxp1b*, *foxp4*, *skor1b*, and *skor2* in *foxp1b*, *foxp4*, *skor1b*, and *skor2* mutants.

(A, B) Expression of *foxp1b* in 5-dpf WT (n = 3) and *foxp1b*$^{Δ26/Δ26}$ (n = 3) mutant larvae. (C, D) Expression of *foxp4* in 5-dpf WT (n = 3) and *foxp4*$^{Δ7/Δ7}$ (n = 3) mutant larvae. (E, F) Expression of *foxp4* in 5-dpf WT (n = 5) and *foxp1b*$^{Δ26/Δ26}$ (n = 5) mutant larvae. (G, H) Expression of *foxp1* in 5-dpf WT (n = 4) and *foxp4*$^{Δ7/Δ7}$ (n = 4) mutant larvae. (I, J) Expression of *skor1b* in 5-dpf WT (n = 3) and *skor1b*$^{Δ10/Δ10}$ (n = 3) mutant larvae. (K, L) Expression of *skor2* in 5-dpf WT (n = 3) and *skor2*$^{Δ8/Δ8}$ (n = 3) mutant larvae. (M, N) Expression of *skor2* in 5-dpf WT (n = 6) and *skor1b*$^{Δ10/Δ10}$ (n = 3) mutant larvae. (O, P) Expression of *skor1b* in 5-dpf WT (n = 3) and *skor2*$^{Δ8/Δ8}$ (n = 3) mutant larvae. The cerebellum region is marked by arrowheads. Scale bar: 100 µm in A (applies to all panels).

Development • Supplementary information

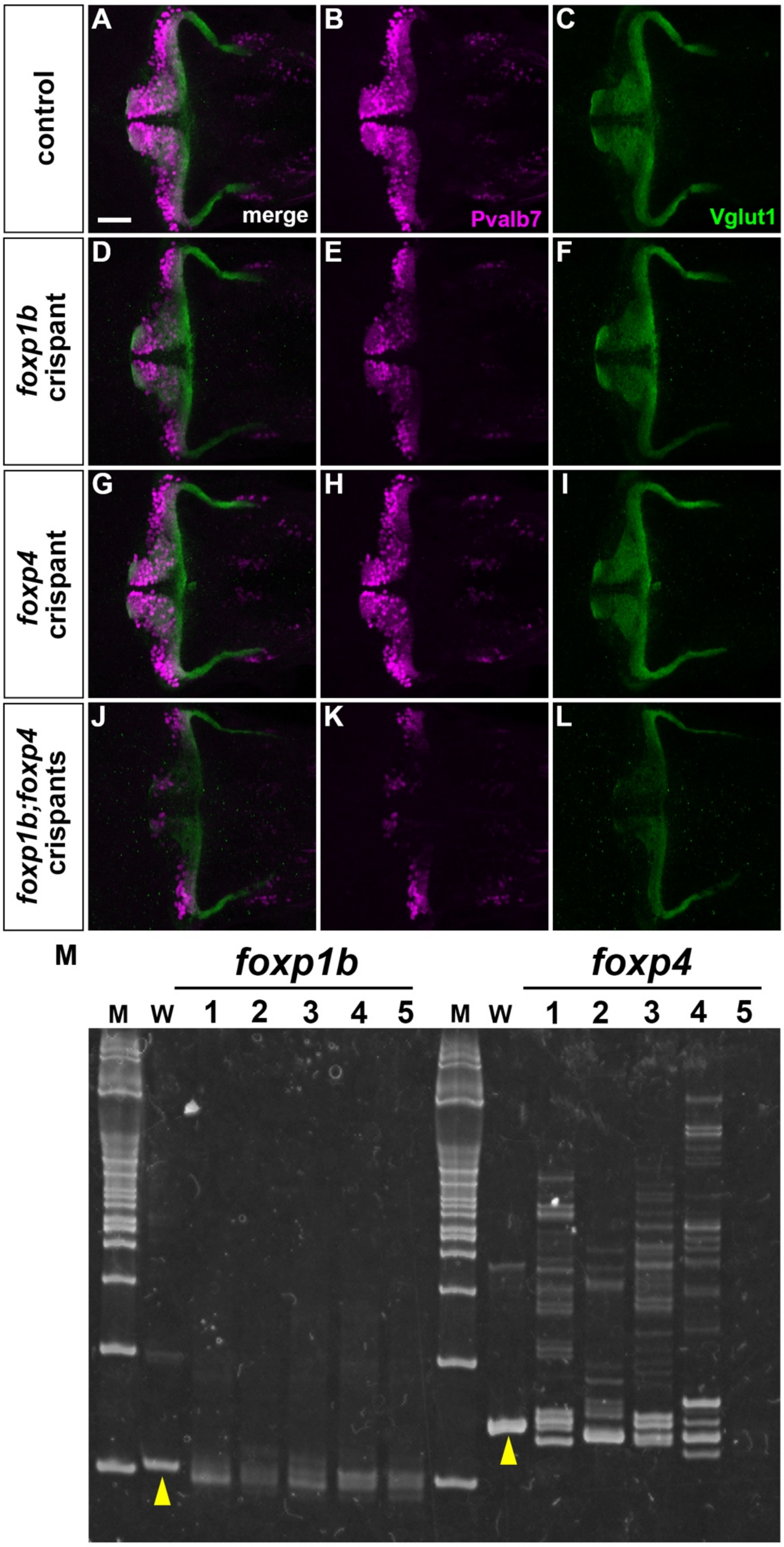
control
foxp1b crispant
foxp4 crispant
foxp1b;foxp4 crispants
A
B
C
D
E
F
G
H
I
J
K
L
merge
Pvalb7
Vglut1
M
foxp1b
foxp4
M W 1 2 3 4 5 M W 1 2 3 4 5

Fig. S8. Phenotypes of *foxp1b* and *foxp4* crispants.
(A-L) Expression of PC marker Pvalb7 (magenta) and GC marker Vglut1 (green) in 5-dpf control (n = 5), *foxp1b* (n = 5), *foxp4* (n = 5), and *foxp1b;foxp4* (n = 5) crispants, which received injection of Cas9 protein, tracrRNA, and *foxp1b*, *foxp4*, or a combination of *foxp1b* and *foxp4* crRNAs. Note that while expression of Pvalb7 was not affected in *foxp1b* or *foxp4* crispants, it was strongly reduced in *foxp1b;foxp4* crispants. Dorsal views with anterior to the left. Scale bar: 50 µm in A (applies to A-L). (M) Genotyping of *foxp1b*;*foxp4* crispants. CRISPR/Cas9-target genomic regions were amplified from five 5-dpf *foxp1b*;*foxp4* crispants by PCR and separated on an acrylamide gel. Note that the crispant larvae had various insertion/deletion (in/del) mutations in their target DNA. Yellow arrows indicated wild-type control PCR products.

Development • Supplementary information

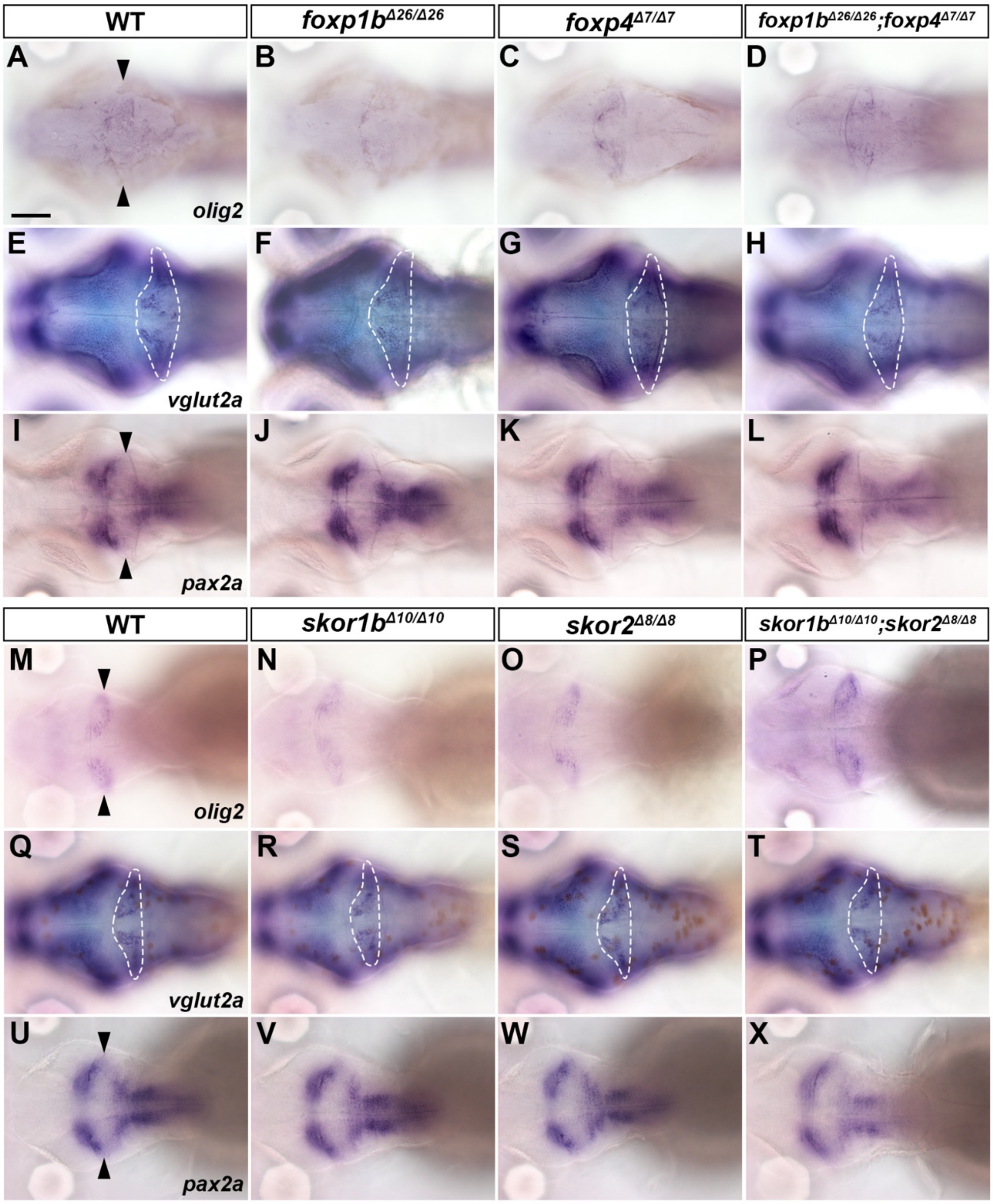

Fig. S9. Expression of *olig2*, *vglut2a*, and *pax2a* in *foxp* and *skor* mutants. Expression of *olig2* (A-D, M-P), *vglut2a* (E-H, Q-T), and *pax2a* (I-L, U-X) in 5-dpf WT, *foxp1b*$^{\Delta26/\Delta26}$, *foxp4*$^{\Delta7/\Delta7}$, and *foxp1b*$^{\Delta26/\Delta26}$;*foxp4*$^{\Delta7/\Delta7}$ mutant larvae (A-L), and WT, *skor1b*$^{\Delta10/\Delta10}$, *skor2*$^{\Delta8/\Delta8}$, and *skor1b*$^{\Delta10/\Delta10}$;*skor2*$^{\Delta8/\Delta8}$ mutant larvae (M-X). The cerebellum region is surrounded or marked by a dotted line and arrowheads, repectively. Scale bars: 100 µm in A (applies to all panels).

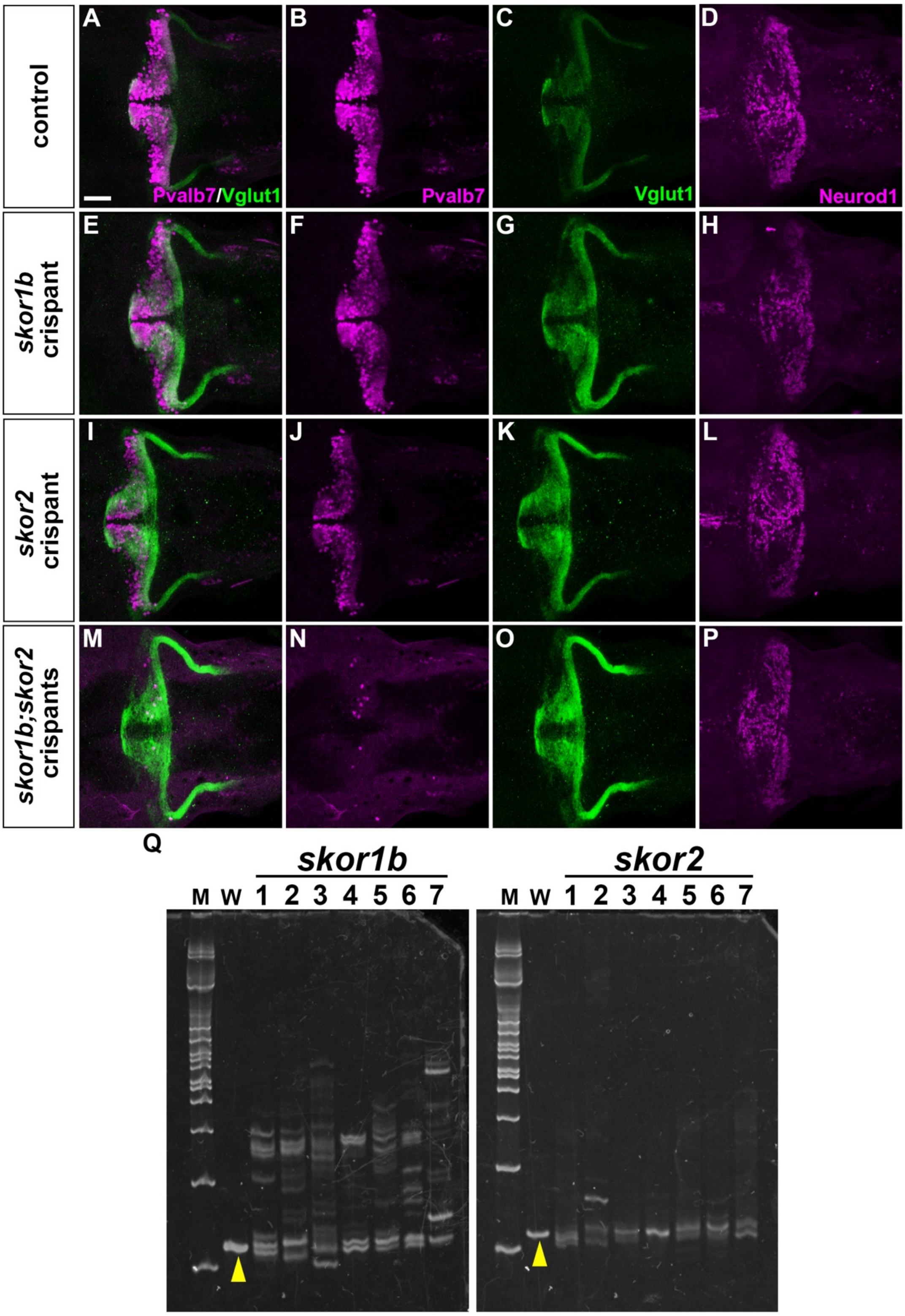

Fig S10. Phenotypes of *skor1b* and *skor2* crispants.

(A-C, E-G, I-K, M-O) Expression of PC marker Pvalb7 (magenta) and GC marker Vglut1 (green) in 5-dpf control (n = 5), *skor1b* (n = 5), *skor2* (n = 5), and *skor1b;skor2*

Development • Supplementary information

(n = 8) crispants, which received injection of Cas9 protein, tracrRNA, and *skor1b*, *skor2*, or a combination of *skor1b* and *skor2* crRNAs. (D, H, L, P) Expression of GC marker Neurod1 in 5-dpf control (*n* = 5) and *skor1b* (*n* = 5), *skor2* (*n* = 5), and *skor1b;skor2* (*n* = 10) crispants. Note that while expression of Pvalb7 was not affected in *skor1b* or *skor2* crispants, it was strongly reduced or absent in *skor1b;skor2* crispants. Neurod1 expression was not affected in all the crispants. Dorsal views with anterior to the left. Scale bars: 50 µm in A (applies to A-P). (M) Genotyping of *skor1b* and *skor2* crispants. CRISPR/Cas9-target genomic regions were amplified from seven 5-dpf *skor1b*;*skor2* crispants by PCR and separated on an acrylamide gel. Note that the crispant larvae had various in/del mutations in their target DNA. Yellow arrows indicated wild-type control PCR products.

Development • Supplementary information

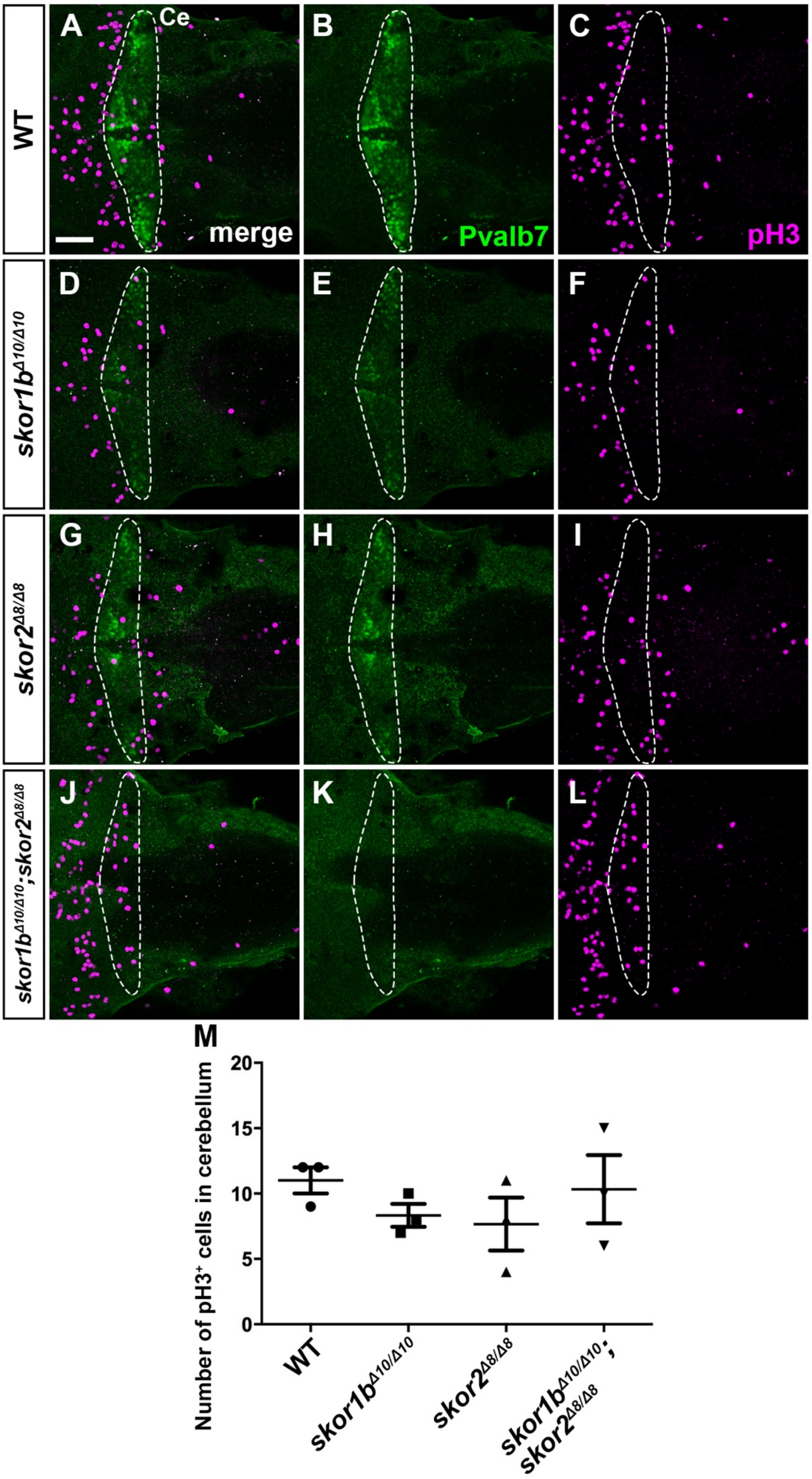

Development • Supplementary information

Fig. S11. Proliferation in wild-type (WT), *skor1b*, *skor2*, and *skor1b/2* mutant cerebellum.

5 dpf WT (A-C), *skor1b* (D-F), *skor2* (G-I), and *skor1b;skor2* (J-L) mutant larvae were immunostained with anti-Pvalb7 and anti-phospho histone H3 (pH3) antibodies. Three larvae for each genotype were analyzed. Dorsal views with anterior to the left. The cerebellum region (Ce) is surrounded by a dotted line. Scale bar: 50 µm in A (applies to A-L). (M) pH3-positive cells in the cerebellum. There was no significant difference between WT, *skor1b*, *skor2*, and *skor1b/2* mutants (one-way ANOVA with Tukey's multiple comparison test).

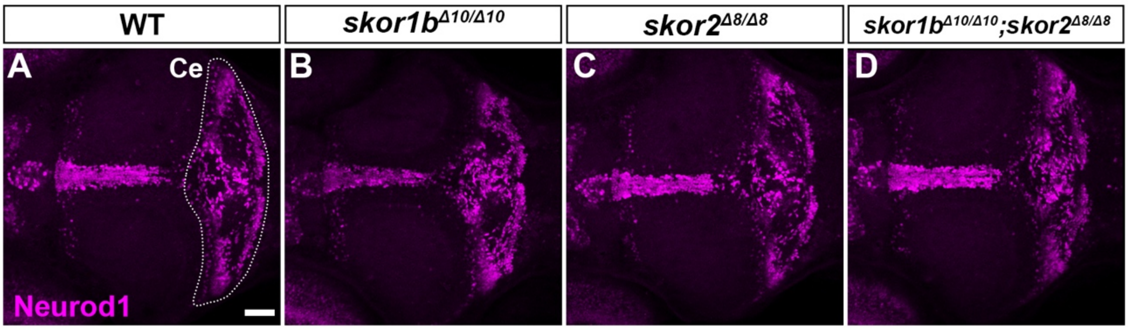

Fig. S12. Neurod1-expressing GCs in *skor1b*, *skor2*, and *skor1b;skor2* mutants. Expression of Neurod1 in the TL and cerebellum of 7-dpf WT (n = 5), *skor1b* (n = 4), *skor2* (n = 5), and *skor1b;skor2* (n = 5) mutant larvae. Dorsal views with anterior to the left. The cerebellum region is surrounded by a dotted line. Scale bar: 50 µm in A (applies to all panels).

Development • Supplementary information

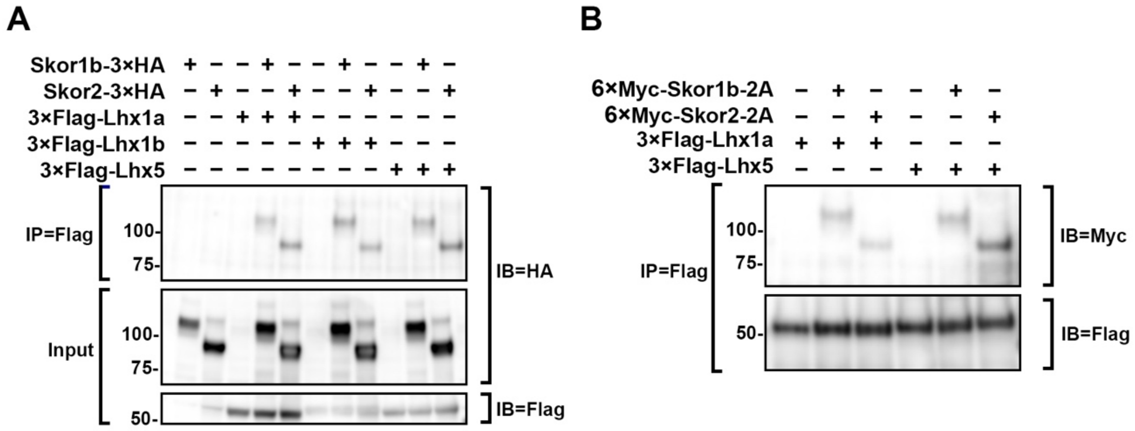

Fig. S13. Interaction of Skor-family proteins with Lhx1-family proteins.
HEK293T cells were transiently transfected with expression plasmids of HA- (A) or Myc (B) epitope-tagged Skor1b, Skor2, and Flag-tagged Lhx1a, Lhx1b, or Lhx5 in the indicated combination. Cell lysates were immunoprecipitated with anti-Flag antibody. Immunoprecipitates or 1/25 of input cell lysates (Input) were immunoblotted with anti-HA, anti-Myc, or anti-Flag antibodies.

Development • Supplementary information

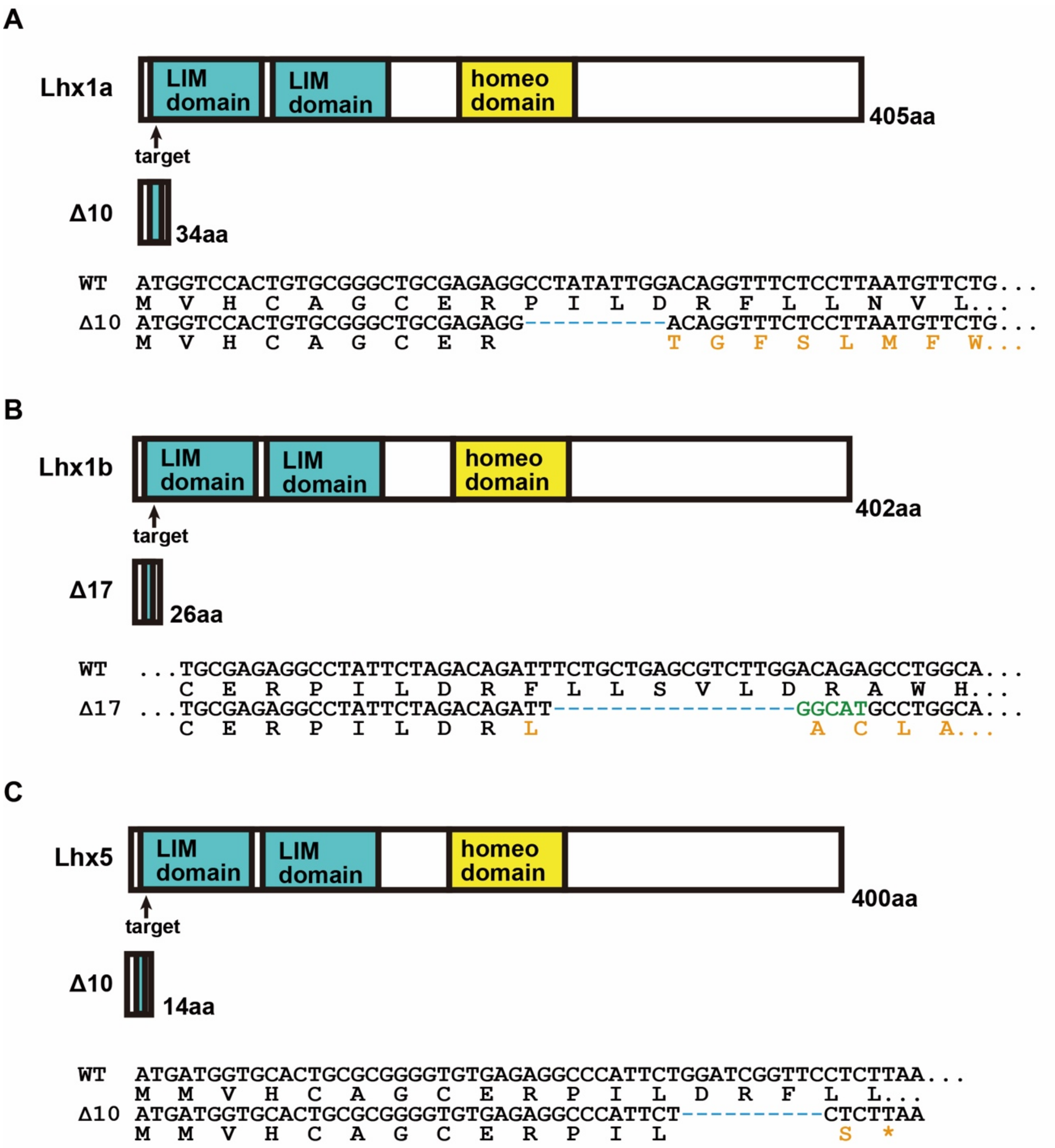

Fig. S14. Structure of wild-type (WT) and mutant Lhx1a, Lhx1b, and Lhx5. Structure of WT and mutant Lhx1a (A), Lhx1b (B), and Lhx5 (C) and nature of mutations generated by the CRISPR/Cas9 method. The positions of the CRISPR/Cas9 targets are shown. The insertion and deletion are marked in green and blue, respectively. The deletion mutations in these genes cause a frameshift, the addition of unrelated amino acids (marked in gray), and a premature stop codon. The mutation of *lhx1a*, *lhx1b*, and *lhx5* results in the addition of 25, 12, and 1 unrelated amino acids, respectively (marked in orange). All the putative mutant proteins lack the LIM domains and the homeodomain that are conserved among Lhx-family proteins.

Development • Supplementary information

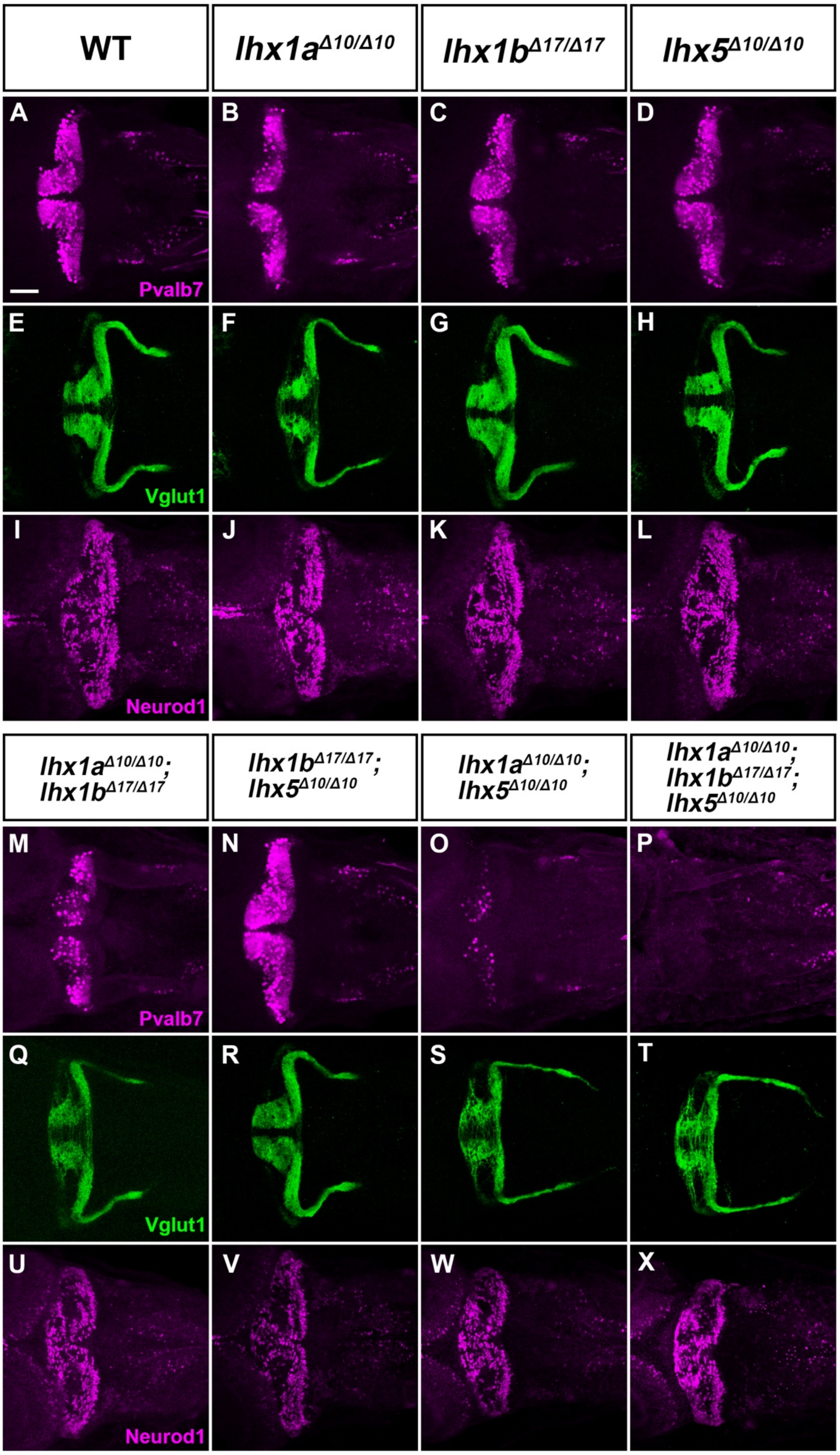

Development • Supplementary information

Fig. S15. Phenotypes of *lhx1a/1b/5* mutants.
5-dpf wild-type (WT) and *lhx1a/1b/5* combinatory mutants were immuno-stained with anti-Pvalb7 (A-D, M-P), Vglut1 (E-H, Q-T), and Neurod1 (I-L, U-X) antibodies. Dorsal views with anterior to the left. The number of examined larvae and larvae showing each expression pattern is shown in Table S1. Note that expression of Pvalb7 was strongly reduced in the *lhx1a;lhx5* mutant and absent in *lhx1a;lhx1b;lhx5* mutants. In the *lhx1a;lhx5* and *lhx1a;lhx1b;lhx5* mutants, Vglut1 and Neurod1 expression was maintained, but the expression regions were also affected, possibly due to malformation of the larval structure. Scale bar: 50 µm in A (applies to all panels).

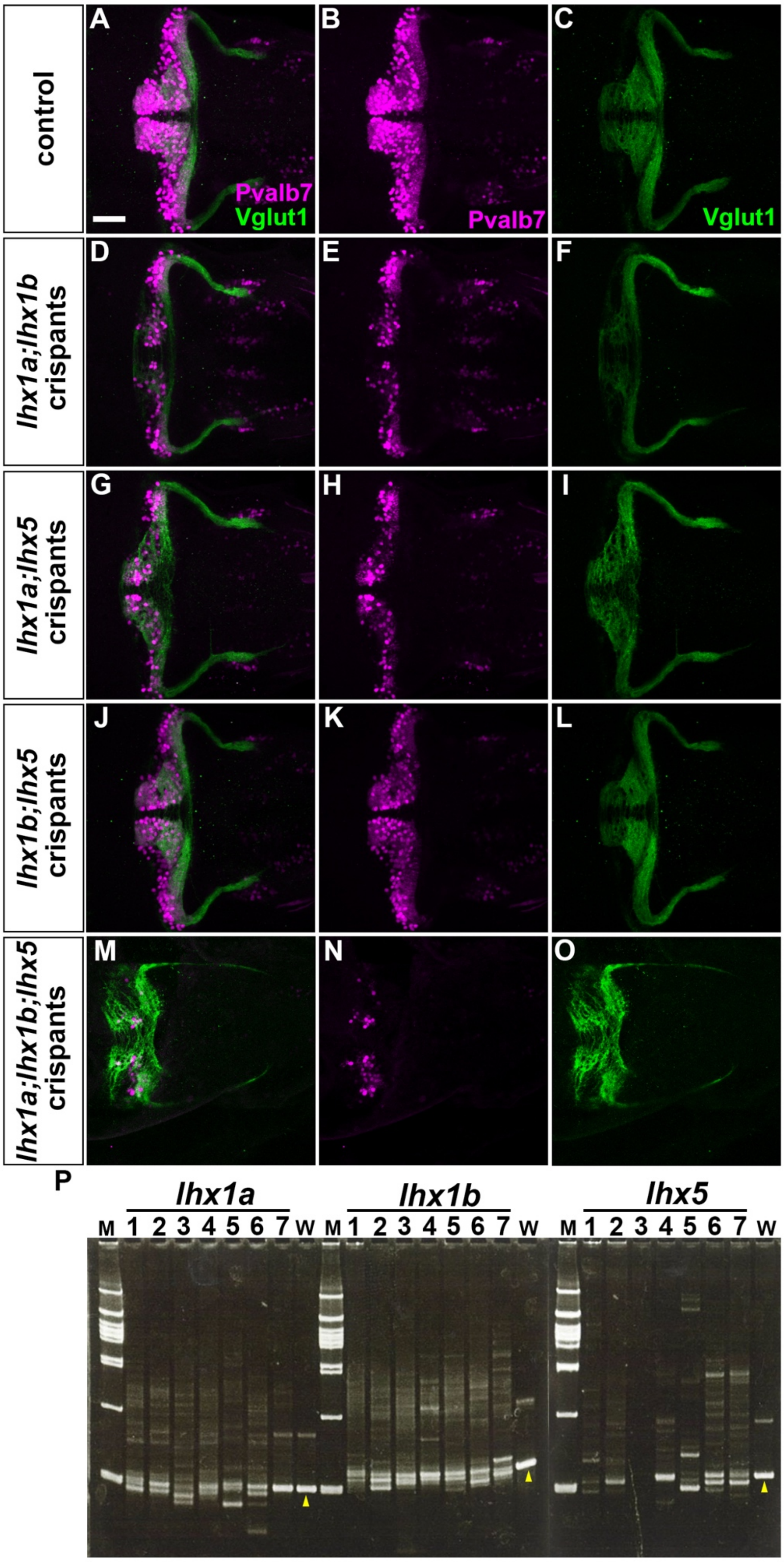

Development • Supplementary information

Fig. S16. Phenotypes of *lhx1a*, *lhx1b* and *lhx5* crispant larvae.
(A -O) Expression of PC marker Pvalb7 (magenta) and GC marker Vglut1 (green) in 5-dpf control (n = 18), *lhx1a;lhx1b* (n = 5), *lhx1a;lhx5* (n = 5), *lhx1b;lhx5* (n = 5), and *lhx1a;lhx1b;lhx5* (n = 13) crispants. Note that expression of Pvalb7 was slightly reduced in *lhx1a;lhx1b* and *lhx1a;lhx5*, but was markedly reduced or absent in *lhx1a;lhx1b;lhx5* crispants. Dorsal views with anterior to the left. Scale bars: 50 µm in A (applies to A-P).
(P) Genotyping of *lhx1a*, *lhx1b* and *lhx5* crispants. CRISPR/Cas9-target genomic regions were amplified from seven 1-dpf *lhx1a*, *lhx1b* and *lhx5* single crispants by PCR and separated on an acrylamide gel. Note that crispant larvae had various in/del mutations in their target DNA. Yellow arrows indicated wild-type control PCR products.

Development • Supplementary information

Table S1. Phenotypes of *lhx1a*, *lhx1b* and *lhx5* mutants

Genotype Marker (stage)	WT	*lhx1a*$^{Δ10/Δ10}$	*lhx1b*$^{Δ17/Δ17}$	*lhx5*$^{Δ10/Δ10}$	*lhx1a*$^{Δ10/Δ10}$; *lhx1b*$^{Δ17/Δ17}$	*lhx1b*$^{Δ17/Δ17}$; *lhx5*$^{Δ10/Δ10}$	*lhx1a*$^{Δ10/Δ10}$; *lhx5*$^{Δ10/Δ10}$	*lhx1a*$^{Δ10/Δ10}$; *lhx1b*$^{Δ17/Δ17}$;*lhx5*$^{Δ10/Δ10}$
Pvalb7 (5 dpf)	+++ (n = 1)	++ (n = 2)	+++ (n = 4)	+++ (n = 4)	+ (n = 2)	+++ (n = 4)	+ (n = 4)	- (n = 3)
Vglut1 (5 dpf)	+++ (n = 1)	++ (n = 3)	+++ (n = 2)	++ (n = 4)	++ (n = 2)	+++ (n = 3)	+ (n = 5)	+ (n = 3)

5-dpf wild-type (WT), *lhx1a/lhx1b/lhx5* single and compound mutant larvae were fixed and analyzed by immunostaining with anti-Pvalb7 anti-Vglut1. Expression levels are indicated by +++, ++, +, and -. +++ indicates expression comparable to that in WT; ++ indicates weak expression, + indicates strongly reduced expression; - indicates little or no expression. The source data are in Table S2.

Table S2. Source data of Table 1, 2, 3, and S1

Available for download at
https://journals.biologists.com/dev/article-lookup/doi/10.1242/dev.202546#supplementary-data

Development • Supplementary information
