# Supplementary material for: Foxp and Skor family proteins control differentiation of Purkinje cells from Ptf1a- and Neurog1-expressing progenitors in zebrafish
Source: Development. 2024 Apr 2;151(7):dev202546. doi: 10.1242/dev.202546 (PMC11057878; doi:10.1242/dev.202546)
Supplement: Supplementary file 1 [file develop-151-202546_review_history.pdf]

Foxp and Skor family proteins control differentiation of Purkinje cells from Ptf1a- and Neurog1-expressing progenitors in zebrafish

Tsubasa Itoh, Mari Uehara, Shinnosuke Yura, Jui Chun Wang, Yukimi Fujii, Akiko Nakanishi, Takashi Shimizu and Masahiko Hibi

DOI: 10.1242/dev.202546

Editor: Steve Wilson

Review timeline

Original submission:	11 April 2023
Editorial decision:	12 June 2023
Resubmission:	15 November 2023
Editorial decision:	12 January 2024
First revision received:	20 February 2024
Editorial decision:	27 February 2024
Second revision received:	27 February 2024
Accepted:	1 March 2024

Original submission

First decision letter

ID#: DEVELOP/2023/201854

MS TITLE: Foxp- and Skor-family proteins control differentiation of Purkinje cells from Ptf1a and Neurogenin1-expressing progenitors in zebrafish

AUTHORS: Tsubasa Itoh, Mari Uehara, Shinnosuke Yura, Jui Chun Wang, Akiko Nakanishi, Takashi Shimizu, and Masahiko Hibi

I have now received all the referees reports on the above manuscript, and have reached a decision. The referees' comments are appended below, or you can access them online: please go to BenchPress and click on the 'Manuscripts with Decisions' queue in the Author Area.

As you will see from their reports, the referees recognise the potential of your work, but they also raise significant concerns about it. Reviewer 1 likes the study but has concerns about the fate mapping data while reviewers 2 and 3 have more substantial concerns about the mutants and phenotypic analyses. Given the nature of these concerns, I am afraid I have little choice other than to reject the paper at this stage.

However, having evaluated the paper, I do recognise the potential importance of this work. I would therefore be prepared to consider as a new submission an extension of this study that contains new experiments, data and discussions and that address fully the major concerns of the referees. The work required goes beyond a standard revision of the paper. Please bear in mind that the referees (who may be different from the present reviewers) will assess the novelty of your work in the context of all previous publications, including those published between now and the time of resubmission.

Reviewer 1

© 2024. Published by The Company of Biologists under the terms of the Creative Commons Attribution License (https://creativecommons.org/licenses/by/4.0/).

Advance summary and potential significance to field

In their manuscript„ Foxp- and Skor-family proteins control differentiation of Purkinje cells fom Ptf1a and Neurogenin1-expressing progenitors in zebrafish" the authors report expression of the transcription factors foxp1b, foxp4, skor1b and skor2 in ptf1a-expressing progenitor cells in the developing zebrafish cerebellum. Based on Cre-recombinase-mediated fate mapping these ptf1a-expressing progenitors give rise to Purkinje neurons, inhibitory interneurons and surprisingly to a subset of granule cells. Mutants were generated for foxp1b, foxp4, skor1b, skor2, lhx1a, lhx1b and khx5 mtants were generated as these transcription factors are expressed in Purkinje neurons. Foxp1b/foxp4 and lhx1a/lhx5 double mutants showed a reduction in Purkinje cell numbers, while these neurons lacked completely in skor1b/skor2 double mutants and lhx1a/lhx1b/lhx5 triple mutants indicating that these transcription factors play a crucial role in the differentiation of Purkinje neurons. Skor2 was found to be able to suppress granule cell differentiation upon misexpression in granule cell progenitors but could not instruct differentiation to mature Purkinje cells. These comprehensive studies are well-executed and displayed at high quality and provide important insight into the molecular regulation of Purkinje cell differentiation, for which a large gap of knowledge exists.

Comments for the author

Some additional experiments in particular to confirm the specificity of Cre-mediated fate mapping need to be performed to support some of the conclusions.

The ptf1a::CreERT2 driver used for data shown in Figure 4 shows significant background activity in the absence of Endoxifen. In addition, in cells expressing gad1b-GFP after loxP-recombination the axons are hardly visible, which could raise doubts whether the expression of GFP is confined to neurons. The specificity of CreERT2-expression in cells expressing ptf1a endogenously and of GFP in neurons needs to be verified experimentally.

This is also important as the authors describe GFP-expressing cells negative for Pvalb7 expression as cerebellar interneurons, which is based on the assumption that GFP-expression occurs specifically in ptf1a-derived inhibitory neurons. Similarly, Kaede-positive granule cells are considered to be derived from ptf1a-expressing progenitor cells for which it is a prerequisite to validate that CreERT2-expression is strictly confined to cells endogenously expressing ptf1a.

Skor1b and skor2, foxp1b and foxp4 as well as lhx1a, lhx1b and lhx5 are expressed in the ptf1a-lineage. Is there a temporal difference in their expression? Are the skor genes expressed earlier or later in cerebellar development compared to foxp1b and foxp4 or the lhx genes? It would be helpful if the authors could mention something about the temporal expression profile of these transcription factors in the zebrafish cerebellum.

Expression of marker genes in foxp1b and foxp4 mutants are shown in table 2. The n-numbers in some cases appear a bit low (e.g. ptf1a at 3dpf n=1). In addition, it would be nice to add data about foxp1b expression in foxp4 mutants and foxp4 expression in foxp1b mutants to address a potential cross-regulation of these related transcription factors. Similarly, to learn about expression of skor1b and skor2 in the respective skor mutants would be of interest to address a potential cross-regulation between these Skor transcription factors.

The double mutants of foxp1b/foxp4 and skor1b/skor2 have been analyzed by expression analysis for Purkinje and granule cell markers. Markers for inhibitory interneurons and eurydendroid cells should be added as the authors suggest that these neurons are derived from ptf1a-progenitors.
In skor1b/skor2 mutants neuroD+-granule cell progenitors are formed in excess, but they fail to differentiate into mature granule cells. What happens to these additional granule cell progenitors? Do they remain in the cerebellum? Are they removed?

The unaffected expression of atoh1 homologs at 3dpf and their downregulation at 5dpf ptf1a;neurog1 mutants is puzzling. In ptf1a;neurog1 mutants skor1b and skor2 expression is lost

© 2024. Published by The Company of Biologists under the terms of the Creative Commons Attribution License (https://creativecommons.org/licenses/by/4.0/).

which should give rise to an excess of granule cell progenitors based on the findings in skor1b/skor2 mutants. How can this be explained?

Minor points:
Fig. 7E, Fig 8E: The spelling should be corrected to ZebrinII
Is there are reason why skor2 and not skor1b was chosen for overexpression studies?

Reviewer 2

Advance summary and potential significance to field

This manuscript describes work investigating gene regulatory relationships that operate in cerebellar neuron development. The approach mostly amounts to examining marker gene expression in zebrafish embryos and larvae that are homozygous for loss-of-function mutations in single genes or pairs of genes that might provide similar functions. The data are fairly extensive and nicely presented, and they provide some new insights, particularly with respect to foxp1b/4 and skor1b/2 genes. However, the analysis is limited to a handful of marker genes with no deeper analysis of cell differentiation with respect to morphology and function, and there are no experiments or data that address transcriptional control mechanisms in any direct fashion. Therefore, although this investigation tackles an important problem and presents some new data, I see it as limited advance in our knowledge.

Comments for the author

Although I think that, for most part, the experiments are reasonably well done and support the main conclusions made by the authors, I do have a few concerns:
1. Presentation of the genetic analyses is not rigorous. Only a single allele for each gene is described and there is little detail provided for the mutant alleles. Any investigation using newly generated mutant alleles should include at least two alleles for each gene recovered from independent founders, a complementation test, a rescue experiment, and an assessment of whether an allele results in nonsense-mediated RNA decay and transcriptional adaptation of related genes that might provide compensatory function.
2. The data presented in Figure 9 are not very convincing. Measuring the area of Neurod1+ cells from these images is probably not a sensitive and accurate measurement. Given the small apparent change in Neurod1+ area, the in situ RNA hybridizations shown in I-M are not likely to reveal any differences. The gain-of-function experiment shown in R-AD needs better explanation. It is not possible to determine how this experiment was performed from the text, figure legend, figure, or methods.
3. It is not stated if the data collection and analyses were blinded to the investigator performing the work.

Reviewer 3

Advance summary and potential significance to field

In their manuscript the authors investigate the role of Foxp- and Skor-family proteins during the differentiation of different cerebellar neuronal subtypes. In mouse Purkinje cell and Granule cells are generated from to distinct proliferative zones (VZ and RL respectively), later Shh expression of Purkinje cells promotes proliferation of granule cell precursors.

In this study the authors suggest that ptf1a+ neural precursor cells can give rise to PCs, INs, and interestingly GCs. Further they suggest that Foxp1b/4 function as key transcriptional regulators for initial PC differentiation from ptf1a/neurgenin1+ progenitors, while Skor1b and Skor2 control PC differentiation by supressing GC fate.
These findings illustrate key differences between zebrafish and mammal cerebellar development and thus have interesting implications for the field.

© 2024. Published by The Company of Biologists under the terms of the Creative Commons Attribution License (https://creativecommons.org/licenses/by/4.0/).

Comments for the author

In general, the manuscript lacks clarity making it hard to follow. Specifically, it would be helpful to include a graphic of the different cells types and transcriptional regulators as Figure 1a to better explain the different experiments performed as part of this manuscript. Additionally, the text should be written more clearly for non-expert readers. For example, the running title is not informative, and the introduction and the abstract lack a description of the lack in knowledge addressed in this manuscript.

The results are described in a very superficial manner and in many instances it is not clear why specific experiments were performed. It is not mentioned in the results section, which species is used. In many instances claims are made without stating specifically the findings. Especially, methods of quantification are omitted where they were performed and in many instances there was no quantification to support the claims made. Orthogonal validation was rarely performed, as indicated by statements like “these mutations are potentially null alleles”. No functional readouts and only one staining of adult animals were included (some statements in the discussion should be changed to be supported by the data shown)

The methods are described superficially. E.g. line 511: “will be described elsewhere”. It is unclear why 3 primers were used to detect neurog1hi1059Tg (lines 581). Catalog numbers for reagents are mostly missing. It is not described how images were quantified. Manual? ImageJ?

189/Fig4 A-F/S: The authors state that GFP expressing cells are GABAergic neurons derived from ptf1a+ progenitors. Giving the leakiness of the construct (about 100 GFP+ cells in DMSO condition vs about 180 cells in endoxifen condition) an orthogonal approach (IHC) to confirm the GABAergic identity would be helpful.

254-256/ Fig 7M-T Table 2: The authors state that there is no effect on expression of GC markers in the double mutant condition (foxp1b;foxp4). In Image 7 Q-T this seems to be not the case for Vglut1. The Table 2provided for quantification lacks the information what technique was applied to quantify the number of cells/staining intensity. Here, an unbiased approach such as AI- or Macro based analysis would be suitable.

263-265 Fig8P: The authors state that the expression pattern of Neurod1 and Vglut1 were affected by the double mutant. Here quantification would be beneficial or the statement should be changed to also acknowledge the presumably changed number of cells.

275/ Fig 8X: The authors state that there is still foxp1b expression in the skor1b, skor2 double mutant. In that image the expression seems to be very low if not non-existing. Orthogonal quantitative approach (eg.WB) should be performed to validate these statements.

277-278 Figure 7 AK and 8 AG. Both images should show WT animals at the same timepoint. However the images are not comparable. Ptf1a expression seems to be inconsistent between these two images. Quantification and/orthogonal quantitative approach would be beneficial here.
360: Functionality and final differentiation of the GS is not shown only images from 5dpf are provided by the authors here.

394: The authors comment on aberrant differentiation of cerebellar neurons. This statement is not supported by any quantification of the data (e.g. cell count etc). This statement should be adjusted or the date should be provided.

Response to Reviewers

MS ID#: DEVELOP/2023/201854

MS TITLE: Foxp- and Skor-family proteins control differentiation of Purkinje cells from Ptf1a and

© 2024. Published by The Company of Biologists under the terms of the Creative Commons Attribution License (https://creativecommons.org/licenses/by/4.0/).

Neurogenin1-expressing progenitors in zebrafish

AUTHORS: Tsubasa Itoh, Mari Uehara, Shinnosuke Yura, Jui Chun Wang, Akiko Nakanishi, Takashi Shimizu, and Masahiko Hibi
We are grateful for the valuable comment from the reviewers. Based on the comments, we have conducted further experiments and revised the manuscript. Point-by-point detailed responded are provided below.

Reviewer 1 Advance Summary and Potential Significance to Field:
In their manuscript, Foxp- and Skor-family proteins control differentiation of Purkinje cells from Ptf1a and Neurogenin1-expressing progenitors in zebrafish" the authors report expression of the transcription factors foxp1b, foxp4, skor1b and skor2 in ptf1a- expressing progenitor cells in the developing zebrafish cerebellum. Based on Cre- recombinase-mediated fate mapping these ptf1a-expressing progenitors give rise to Purkinje neurons, inhibitory interneurons and surprisingly to a subset of granule cells. Mutants were generated for foxp1b, foxp4, skor1b, skor2, lhx1a, lhx1b and khx5 mtants were generated as these transcription factors are expressed in Purkinje neurons. Foxp1b/foxp4 and lhx1a/lhx5 double mutants showed a reduction in Purkinje cell numbers, while these neurons lacked completely in skor1b/skor2 double mutants and lhx1a/lhx1b/lhx5 triple mutants indicating that these transcription factors play a crucial role in the differentiation of Purkinje neurons. Skor2 was found to be able to suppress granule cell differentiation upon misexpression in granule cell progenitors but could not instruct differentiation to mature Purkinje cells. These comprehensive studies are well- executed and displayed at high quality and provide important insight into the molecular regulation of Purkinje cell differentiation, for which a large gap of knowledge exists.

We appreciate the positive opinions from the Reviewers regarding the significance and novelty of our findings, and value their criticisms and suggestions.

Reviewer 1 Comments for the Author:

Some additional experiments in particular to confirm the specificity of Cre-mediated fate mapping need to be performed to support some of the conclusions.

The ptf1a::CreERT2 driver used for data shown in Figure 4 shows significant background activity in the absence of Endoxifen. In addition, in cells expressing gad1b-GFP after loxP-recombination the axons are hardly visible, which could raise doubts whether the expression of GFP is confined to neurons. The specificity of CreERT2-expression in cells expressing ptf1a endogenously and of GFP in neurons needs to be verified experimentally. This is also important as the authors describe GFP-expressing cells negative for Pvalb7 expression as cerebellar interneurons, which is based on the assumption that GFP- expression occurs specifically in ptf1a-derived inhibitory neurons. Similarly, Kaede-positive granule cells are considered to be derived from ptf1a-expressing progenitor cells for which it is a prerequisite to validate that CreERT2-expression is strictly confined to cells endogenously expressing ptf1a.

To clarify CreERT2 expression in the ptf1a::mCherry-T2A-CreERT2 line within the *ptf1a* expression domain, we conducted the following additional experiments. First, we generated *ptf1a*$^{Tg(hsp70l\text{-}EGFP)}$ line by knocking in an EGFP expression cassette into the *ptf1a* gene locus. We confirmed that EGFP in the *ptf1a*$^{Tg(hsp70l\text{-}EGFP)}$ line recapitulates *ptf1a* expression. We found that the expression of mCherry and *CreERT2* mRNA in the cerebellum of the Ptf1a::mCherry-T2A-CreERT2 line coincided with the expression of EGFP in the *ptf1a*$^{Tg(hsp70l\text{-}EGFP)}$ line. These results indicate that the ptf1a::mCherry-T2A- CreERT2 line expressed CreERT2 in *ptf1a*$^{+}$ neural progenitors in the cerebellum. We have presented the data as Fig. S4 and have described this issue on page 8, line 170 to line 176.

Skor1b and skor2, foxp1b and foxp4 as well as lhx1a, lhx1b and lhx5 are expressed in the ptf1a-lineage. Is there a temporal difference in their expression? Are the skor genes expressed earlier or later in cerebellar development compared to foxp1b and foxp4 or the lhx genes? It would be helpful if the authors could mention something about the temporal expression profile of these transcription factors in the zebrafish cerebellum.

© 2024. Published by The Company of Biologists under the terms of the Creative Commons Attribution License (https://creativecommons.org/licenses/by/4.0/).

We analyzed the expression of *foxp1b/4*, *skor1b/2*, and *lhx1a/1b/5* from 1 dpf to 5 dpf during early development. Expression of *foxp1b/4*, *skor1b/2*, and *lhx1a/1b* was similarly observed from 2 dpf, coinciding with the detection of *ptf1a* expression in the cerebellum. *lhx5* expression in the cerebellum was weak and hard to detect. We have shown the data as Fig. S5 and have described this issue on page 10, line 214-216 and page 20 line 454- 455.

Expression of marker genes in foxp1b and foxp4 mutants are shown in table 2. The n- numbers in some cases appear a bit low (e.g. ptf1a at 3dpf n=1). In addition, it would be nice to add data about foxp1b expression in foxp4 mutants and foxp4 expression in foxp1b mutants to address a potential cross-regulation of these related transcription factors. Similarly, to learn about expression of skor1b and skor2 in the respective skor mutants would be of interest to address a potential cross-regulation between these Skor transcription factors.

We analyzed *foxp4* expression in *foxp1b* mutants, *foxp4* expression in *foxp1b* mutants, *skor2* expression in *skor1b* mutants, and *skor1b* expression in *skor2* mutants. Compared to wild-type larvae, none of these expressions were altered in these mutants. This suggests that the cross-regulation (compensatory gene expression) did not occur in the mutants we established in this study. We have shown the data as Fig. S7 and have described this issue on page 11, line 240-243.

The double mutants of foxp1b/foxp4 and skor1b/skor2 have been analyzed by expression analysis for Purkinje and granule cell markers. Markers for inhibitory interneurons and eurydendroid cells should be added as the authors suggest that these neurons are derived from ptf1a-progenitors.

We analyzed the expression of EC (eurydendroid cell) markers *vglut2a* and *olig2*, and IN (inter neuron) marker *pax2* in *foxp1b;foxp4* and *skor1b;skor2* mutants. Expression of these makers was unaffected in both mutants. The data suggest that Foxp1b/4 and Skor1b/2 are necessary for proper differentiation of PCs, but not ECs or INs, in the cerebellum. We have presented the data as Fig. S9, Table 2, and Table 3) and have described this issue on page 11, line 253-255; page 12, line 258-261, and page 12, 271-274.

In skor1b/skor2 mutants neuroD+-granule cell progenitors are formed in excess, but they fail to differentiate into mature granule cells. What happens to these additional granule cell progenitors? Do they remain in the cerebellum? Are they removed?

We analyzed Neurod1 expression in the cerebellum at 7 dpf. Although there was an increase in Neurod1^{+} granule cells (GCs) increased at 5 dpf in *skor1b/skor2* mutants, this increase was not sustained at 7 dpf. The data suggest that those GCs may have died. We performed immunostaining on *skor1b/skor2* mutants with an anti-cleaved capase3 antibody. However, we observed no significant increase in cleaved caspase 3^{+} apoptotic cells in the cerebellum. Apoptosis, if it occurred, was below the detection level. We hypothesize that the ectopically formed GCs might die off gradually. Given that the immunostaining data is negative, we have opted not to include it. We have presented the data of Neurod1^{+} GCs at 7 dpf as Fig. 9R and S12, detailed this issue on page 14, line 304 to 305, and discussed it on page 19, line 433-438.

The unaffected expression of atoh1 homologs at 3dpf and their downregulation at 5dpf ptf1a;neurog1 mutants is puzzling. In ptf1a;neurog1 mutants skor1b and skor2 expression is lost which should give rise to an excess of granule cell progenitors based on the findings in skor1b/skor2 mutants. How can this be explained?

In *ptf1a;neurog1* mutants, while the expression of *skor1b* and *skor2* was absent, we did not observe an increase in *atoh1*$^{+}$ GC progenitors or Neurod1^{+} GCs; instead, there was a decrease (Fig. 3, Table 1). This finding is in contrast with the excess GCs in the *skor1b;skor2* mutants (Fig. 9, Table 3). However, this may be due to the absence of *ptf1a*$^{+}$ neural progenitors, which give rise to excess GCs. We have discussed this issue on page 19, line 439-443.

Minor points:

Fig. 7E, Fig 8E: The spelling should be corrected to ZebrinII
We have corrected them.

© 2024. Published by The Company of Biologists under the terms of the Creative Commons Attribution License (https://creativecommons.org/licenses/by/4.0/).

Is there are reason why skor2 and not skor1b was chosen for overexpression studies?
Although we generated transgenic fish designated to express *skor1b* under GAL4-UAS control, they did not show robust expression. We believe that the data on *skor2* ectopic expression was sufficient to support our conclusion.

Reviewer 2 Advance Summary and Potential Significance to Field:
This manuscript describes work investigating gene regulatory relationships that operate in cerebellar neuron development. The approach mostly amounts to examining marker gene expression in zebrafish embryos and larvae that are homozygous for loss-of- function mutations in single genes or pairs of genes that might provide similar functions. The data are fairly extensive and nicely presented, and they provide some new insights, particularly with respect to foxp1b/4 and skor1b/2 genes. However, the analysis is limited to a handful of marker genes with no deeper analysis of cell differentiation with respect to morphology and function, and there are no experiments or data that address transcriptional control mechanisms in any direct fashion. Therefore, although this investigation tackles an important problem and presents some new data, I see it as limited advance in our knowledge.

We agree that it is important to provide a detailed mechanistic understanding of cell differentiation mediated by Foxp1b/4 and Skor1b/2. However, this study also includes data on the production of diverse cells by *ptf1a*$^{+}$ neural progenitors, and I believe that elucidating the differentiation control mechanisms by Foxp and Skor exceeds the scope of a single paper.

Reviewer 2 Comments for the Author:
Although I think that, for most part, the experiments are reasonably well done and support the main conclusions made by the authors, I do have a few concerns:

1. Presentation of the genetic analyses is not rigorous. Only a single allele for each gene is described and there is little detail provided for the mutant alleles. Any investigation using newly generated mutant alleles should include at least two alleles for each gene recovered from independent founders, a complementation test, a rescue experiment, and an assessment of whether an allele results in nonsense-mediated RNA decay and transcriptional adaptation of related genes that might provide compensatory function.

Given the potential off-target effects of CRISPR/Cas9, it may be important to generate and analyze multiple alleles. The mutants of the *foxp*, *skor*, and *lhx* family genes we generated were maintained over several generations in the lab and are unlikely to contain other mutations. Nevertheless, to exclude the possibility of off-target mutations in other genes due to off-target effects, we created crispants (F0 mutants) using crRNAs targeting different sequences from those mutated in the stable mutants of *foxp1b*, *foxp4*, *skor1b*, and *skor2*, and performed phenotypic analysis. PCs were reduced *foxp1b;foxp4* crispants and PCs were strongly reduced or absent in *skor1b;skor2* crispants were replicated. For *lhx1a/lhx1b/lhx5*, crispants were also produced using the crRNAs utilized for the generation of the stable mutants, and a significant reduction in Purkinje cells was observed in *lhx1a;lhx1b;lhx5* crispants, similar to the stable mutants. These data suggest that the phenotypes observed in the stable mutants are not due to off-target effects, but are a result of specific loss of these genes.

Furthermore, we examined the expression of *foxp1b* and *foxp4* in *foxp1b* mutants, *foxp4* in *foxp4* mutants, *skor1b* and *skor2* in *skor1b* mutants, and *skor1b* and *skor2* in *skor2* mutants, and found that the mutations did not affect the expression of the targeted gene or related genes (Fig. S7). This indicates that nonsense-mediated RNA decay or compensatory gene expression did not occur in these mutants.

The genes analyzed in this study are specifically expressed in Purkinje cells during their differentiation process, making rescue experiments challenging. We believe that the experiments with crispants and the characterization of the stable mutants are sufficient to validate the data obtained from the stable mutants.

We have presented these data as Fig. S8 (*foxp1b;foxp4* cristants), Fig. S10 (*skor1b;skor2* crispants, Fig. S16 (*lhx1a/lhx1b:lhx5* crispants), and Fig. S7 (expression of *foxp1b*, *foxp4*, *skor1b*, and *skor2* in

© 2024. Published by The Company of Biologists under the terms of the Creative Commons Attribution License (https://creativecommons.org/licenses/by/4.0/).

foxp and *skor* mutants). We have described the data on page 11, line 240-243; page 11, line 250-253; page 12, line 265-267, and page 20, line 455-459.

2. The data presented in Figure 9 are not very convincing. Measuring the area of Neurod1+ cells from these images is probably not a sensitive and accurate measurement. Given the small apparent change in Neurod1+ area, the in situ RNA hybridizations shown in I-M are not likely to reveal any differences. The gain-of-function experiment shown in R-AD needs better explanation. It is not possible to determine how this experiment was performed from the text, figure legend, figure, or methods.

In *skor1b;skor2* mutants, while there was an increase in GCs expressing Neurod1, a marker for immature and mature GCs, there was no increase in the expression of *cbln12* and *vglut1*, which are markers for mature GCs. The data are not contradictory. To investigate the fate of the excess GCs, we examined the number of Neurod1^{+} GCs at 7 dpf but observed no significant change compared to wild type. Therefore, it is considered that in *skor1b;skor2* mutants, GCs were produced in excess but were subsequently eliminated by some mechanism.

To explain the ectopic expression experiment (gain-of-function experiments) of *skor2*, we introduced a new Fig. 9S and added a description on page 14, line 305-308. In this experiment, the ectopic expression of *skor2* in GC progenitors via the Gal4-UAS system repressed GC differentiation. The original manuscript used UAS:RFP as a control, but in this instance, for comparison with Skor2, we utilized an unrelated biotin ligase A (BirA) gene as the control.

3. It is not stated if the data collection and analyses were blinded to the investigator performing the work.

While the data collection and analysis were not performed with blinding, we have employed rigorous standardized protocols throughout the study to maintain consistency and objectivity in our experimental procedures. Additionally, multiple investigators cross- validated the data to ensure reliability and to mitigate any potential bias.

Reviewer 3 Advance Summary and Potential Significance to Field:
In their manuscript the authors investigate the role of Foxp- and Skor-family proteins during the differentiation of different cerebellar neuronal subtypes. In mouse Purkinje cell and Granule cells are generated from to distinct proliferative zones (VZ and RL respectively), later Shh expression of Purkinje cells promotes proliferation of granule cell precursors.

In this study the authors suggest that ptf1a+ neural precursor cells can give rise to PCs, INs, and interestingly GCs. Further they suggest that Foxp1b/4 function as key transcriptional regulators for initial PC differentiation from ptf1a/neurgenin1+ progenitors, while Skor1b and Skor2 control PC differentiation by suppressing GC fate. These findings illustrate key differences between zebrafish and mammal cerebellar development and thus have interesting implications for the field.

We believe that by comparing the findings obtained from zebrafish with those from mammals, we have been able to approach the elucidation of cerebellar neuron differentiation mechanisms that have not been clarified until now.

Reviewer 3 Comments for the Author:
In general, the manuscript lacks clarity making it hard to follow. Specifically, it would be helpful to include a graphic of the different cell types and transcriptional regulators as Figure 1a to better explain the different experiments performed as part of this manuscript. Additionally, the text should be written more clearly for non-expert readers. For example, the running title is not informative, and the introduction and the abstract lack a description of the lack in knowledge addressed in this manuscript.

We have created a new diagram explaining the relationship between cerebellar neuronal differentiation and transcription regulators (Fig. 1A). We have also endeavored to write the abstract in a manner that is understandable to those outside the field. The running title has been changed to "Purkinje cell gene regulators".

© 2024. Published by The Company of Biologists under the terms of the Creative Commons Attribution License (https://creativecommons.org/licenses/by/4.0/).

The results are described in a very superficial manner and in many instances it is not clear why specific experiments were performed. It is not mentioned in the results section, which species is used. In many instances claims are made without stating specifically the findings. Especially, methods of quantification are omitted where they were performed and in many instances there was no quantification to support the claims made. Orthogonal validation was rarely performed, as indicated by statements like “these mutations are potentially null alleles”. No functional readouts and only one staining of adult animals were included (some statements in the discussion should be changed to be supported by the data shown).

It is not clear if we have completely understood the reviewer's comments, but we wish to clarify that the species used in this study was zebrafish. The names of mutant and transgenic zebrafish are noted following zebrafish nomenclature rules. We have referenced and have indicated this clearly. If there are any gaps in our understanding, we welcome further instruction.

To quantify *in situ* hybridization and immunohistochemistry data with some exceptions, image data were imported into the image processing software ImageJ (https://imagej.net/ij/) and Fiji (Fiji (https://fiji.sc), and the cerebellar region was cropped from the image. Binarization was performed after manually setting an arbitrary threshold. The areas with signals were measured. The average area of the data obtained for each was calculated, and it was compared with the data from WT. Ratings were given based on comparison with WT data: +++, ++, +, or - for values of 0.8 or higher, 0.5 to 0.8, 0.01 to 0.5, and less than 0.01, respectively. Due to a high background, the expression of *vglut2a* in *ptf1a;neurog1*, *foxp1b;foxp4*, and *skor1b;skor2* mutants, as well as the expression of *foxp4* and *reln* in *skor1b;skor2* mutants was visually assessed and rated (page 28, line 652-661). Raw data for quantification will be provided as a supplemental file (Quantification data.xlsx).

Regarding the mutants created in this study, nonsense mutations have been introduced on the N-terminal side of the essential functional domain of the transcription factors, suggesting that they are loss-of-function mutants. Immunostaining for Foxp1b, Skor1b, and Skor2 failed to detect the antigen epitope of these proteins in the nucleus. However, as the reviewer pointed out, it has not been proven that these are null mutants. Therefore, we have decided to avoid using the term "potentially null alleles". Furthermore, our findings indicate that in *foxp1b*, *foxp4*, *skor1b*, and *skor2* single mutants, neither nonsense-mediated RNA decay nor compensatory gene expression was observed (Fig. S7). Thus, these mutants can be classified as loss-of-function, lacking functional compensation. We further confirmed a reduction of PCs in *foxp1b;foxp4* F0 mutants (crispants) and *skor1b;skor2* crispants (please see our comments for Reviewer 2).

The methods are described superficially. E.g. line 511: “will be described elsewhere”. It is unclear why 3 primers were used to detect neurog1hi1059Tg (lines 581). Catalog numbers for reagents are mostly missing. It is not described how images were quantified. Manual? ImageJ?

We have detailed the method for creating the CreERT2 line Tg(5xUAS-hsp70l:mCherry- T2A-CreERT2) in the MATERIALS and METHODS section (page 22, lines 512-519). To detect retrovirus-induced *neurog1* mutant alleles, we used three primers: two to amplify the wild-type genome around the virus integration site, and one to bind to the viral DNA. This is explained in the MATERIALS and METHODS section (page 26, lines 599-606). Additionally, we have included catalog numbers for reagents where possible. We have explained how images were quantified above.

189/Fig4 A-F/S: The authors state that GFP expressing cells are GABAergic neurons derived from ptf1a+ progenitors. Giving the leakiness of the construct (about 100 GFP+ cells in DMSO condition vs about 180 cells in endoxifen condition) an orthogonal approach (IHC) to confirm the GABAergic identity would be helpful.

The leakiness of the construct is due to the high expression level of CreERT2 and the high recombination efficiency of the loxP reporter gene. However, this is unrelated to the specificity of the reporter in the *TgBAC(gad1b:LOXP-DsRed-LOXP-GFP)nns26Tg* line. Since it has already been reported that the reporter expression in this line is specific to GABAergic neurons (Satou et al. 2013), we do not believe that confirmation by immunostaining is necessary.

© 2024. Published by The Company of Biologists under the terms of the Creative Commons Attribution License (https://creativecommons.org/licenses/by/4.0/).

254-256/ Fig 7M-T Table 2: The authors state that there is no effect on expression of GC markers in the double mutant condition (foxp1b;foxp4). In Image 7 Q-T this seems to be not the case for Vglut1. The Table 2 provided for quantification lacks the information what technique was applied to quantify the number of cells/staining intensity. Here, an unbiased approach such as AI- or Macro based analysis would be suitable.

As previously mentioned, we quantified immunohistochemistry data and assessed phenotypes based on the average expression domains. We have confirmed that the expression domain of Vglut1 was not altered in *foxp1b;foxp4* mutants. The apparent reduction in Vglut1 expression is likely attributable to a significant reduction in PCs in these mutants, leading to abnormal GC axonal trajectory and a shift in the Vglut1 expression domain. Neurod1^{+} GCs remained unaffected in these mutants. We have addressed this issue on page 11, lines 255-page 12, line 258. The quantification method was applied similarly to the data in Table 2, with a few exceptions. Due to a high background, the expression of *vglut2a* in *ptf1a;neurog1*, *foxp1b;foxp4*, and *skor1b;skor2* mutants, as well as the expression of *foxp4* and *reln* in *skor1b;skor2* mutants was visually assessed and rated (page 28, line 652-661).

263-265 Fig8P: The authors state that the expression pattern of Neurod1 and Vglut1 were affected by the double mutant. Here quantification would be beneficial or the statement should be changed to also acknowledge the presumably changed number of cells.

Just as described above, we quantified immunohistochemistry data and evaluated phenotypes. We have confirmed that the expression domain size of Vglut1 was not altered in *skor1b;skor2* mutants. In these mutants, PCs were absent, and GC axonal trajectory was abnormal. We have described this issue on page 12, line 267-271.

275/ Fig 8X: The authors state that there is still foxp1b expression in the skor1b, skor2 double mutant. In that image the expression seems to be very low if not non-existing. Orthogonal quantitative approach (eg.WB) should be performed to validate these statements.

We conducted a re-analysis of *foxp1b* expression in *skor1b;skor2* mutants and presented the results in Figure 8X. The expression was notably reduced. As mentioned in the original manuscript, it cannot be ruled out that *foxp1b* may be expressed in ectopic GCs, but its weak expression prevents the specific identification of expressing cells. Therefore, we opted to report only the decrease in expression (page 12, line 279-page 12, 280). Performing Western blotting for Foxp1b in zebrafish mutants presents quantitative challenges, and it was deemed impractical to carry out this experiment.

277-278 Figure 7 AK and 8 AG. Both images should show WT animals at the same timepoint. However the images are not comparable. Ptf1a expression seems to be inconsistent between these two images. Quantification and/orthogonal quantitative approach would be beneficial here.

Regarding Fig. 8AG-AJ (detection of *ptf1a* at 5 dpf by in situ hybridization), there were a significant number of remaining pigment cells, making the signal difficult to observe. We repeated this experiment and included new data. Quantification results indicated that the expression of *ptf1a* did not change significantly in both *foxp1b;foxp4* mutants and *skor1b;skor2* mutants (Table 2). We have described this issue on page 13, line 284-287.

360: Functionality and final differentiation of the GS is not shown only images from 5dpf are provided by the authors here.

We are somewhat uncertain about the intended meaning of the comments. Is it a question about whether there is enough evidence to confirm the final functional differentiation of GCs derived from *ptf1a*-expressing cells? In our Fig. 5 data, we demonstrate the differentiation of cells expressing cbln12:Kaede reporter from *ptf1a*-expressing cells. We have previously reported that *cbln12* is specifically expressed in GCs (Takeuchi et al., 2017) and the *cbln12* promoter can specifically drive expression in GCs in the cerebellum (Dohaku et al., 2019). Additionally, these cells clearly extended parallel fibers. While we have not extensively examined their functionality, we believe we have provided sufficient evidence of their identity as GCs.

© 2024. Published by The Company of Biologists under the terms of the Creative Commons Attribution License (https://creativecommons.org/licenses/by/4.0/).

394: The authors comment on aberrant differentiation of cerebellar neurons. This statement is not supported by any quantification of the data (e.g. cell count etc). This statement should be adjusted or the date should be provided.

In *skor1b;skor2* mutants, we quantitatively depicted the absence of PCs and the presence of extra immature GCs. In the cerebellum, both *foxp1b* and *foxp4* are specifically expressed in PCs. It is reasonable to assume that there would be an impact on their expression in *skor1b;skor2* mutants, where PCs are absent. If there has been a misunderstanding of the comment's intent, we would greatly appreciate clarification.

Resubmission

First decision letter

MS ID#: DEVELOP/2023/202546

MS TITLE: Foxp- and Skor-family proteins control differentiation of Purkinje cells from Ptf1a and Neurogenin1-expressing progenitors in zebrafish

AUTHORS: Tsubasa Itoh, Mari Uehara, Shinnosuke Yura, Jui Chun Wang, Yukimi Fujii, Akiko Nakanishi, Takashi Shimizu, and Masahiko Hibi

You will be pleased to hear that the reviewers are mostly happy with your revisions and there are just a couple of minor points to consider/address before we proceed to publication. The referees' comments are appended below, or you can access them online: please go to BenchPress and click on the 'Manuscripts with Decisions' queue in the Author Area.

Reviewer 1

Advance summary and potential significance to field

In their manuscript “Foxp- and Skor-family proteins control differentiation of Purkinje cells fom Ptf1a and Neurogenin1-expressing progenitors in zebrafish” the authors report expression of the transcription factors foxp1b, foxp4, skor1b and skor2 in ptf1a-expressing progenitor cells in the developing zebrafish cerebellum. Based on Cre-recombinase-mediated fate mapping these ptf1a-expressing progenitors give rise to Purkinje neurons, inhibitory interneurons and surprisingly to a subset of granule cells. Mutants were generated for foxp1b, foxp4, skor1b, skor2, lhx1a, lhx1b and khx5 mtants were generated as these transcription factors are expressed in Purkinje neurons. Foxp1b/foxp4 and lhx1a/lhx5 double mutants showed a reduction in Purkinje cell numbers, while these neurons lacked completely in skor1b/skor2 double mutants and lhx1a/lhx1b/lhx5 triple mutants indicating that these transcription factors play a crucial role in the differentiation of Purkinje neurons. Skor2 was found to be able to suppress granule cell differentiation upon misexpression in granule cell progenitors but could not instruct differentiation to mature Purkinje cells. These comprehensive studies are well-executed and displayed at high quality and provide important insight into the molecular regulation of Purkinje cell differentiation, for which a large gap of knowledge exists.

Comments for the author

The revised version of the manuscript by Itoh et al. reads well and has covered well most of my points of concern. The generation of the knock-in line of GFP into the endogenous locus of ptf1a is very convincing and demonstrates well the specificity of Cre expression in ptf1a-expressing cells.

© 2024. Published by The Company of Biologists under the terms of the Creative Commons Attribution License (https://creativecommons.org/licenses/by/4.0/).

The expression data shown in Table 1-3 is very comprehensive yet the authors did not address my concern that their evaluation of expression strength of some of these markers is based on a very small number of larvae analyzed. In some cases only a single larva was analyzed. Maybe this is a misunderstanding on my side. Does n=1 mean one staining procedure with several larvae? Then the number of larvae studied should be provided.

Minor points:

Some of the selected references should be corrected or updated. For example:

Line 63/64 Expression of atoh1 and ptf1a genes in the URL and VZ of the cerebellum was also reported for zebrafish (Chaplin et al., 2010, Kanie et al., 2010).
Expression of atoh1 genes in the URL has been reported earlier. e.g. Köster et al., 2001, Adolf et al., 2004, Volkmann et al., 2008.

Line 364: …and SHH signaling is not activated in the zebrafish cerebellum (Biechl et al., 2016..).

Biechl et al. showed shh expression in eurydendroid cells in the cerebellum (see supplementary data).

Reviewer 2

Advance summary and potential significance to field

I appreciate the considerable effort made by the authors for this revised manuscript. They thoughtfully addressed all comments from the prior review. This is a quite comprehensive and rigorous study and it certainly reveals new information about cerebellar neuron development and potential differences between mice and zebrafish. Although I had previously questioned the mechanistic depth and potential impact of this work, I acknowledge and agree with the more positive assessments made by the other reviewers.

Comments for the author

I have no further comments regarding additional changes and potential revision.

First revision

Author response to reviewers' comments

Response to Reviewers

MS ID#: DEVELOP/2023/202546

MS TITLE: Foxp- and Skor-family proteins control differentiation of Purkinje cells from Ptf1a and Neurogenin1-expressing progenitors in zebrafish

AUTHORS: Tsubasa Itoh, Mari Uehara, Shinnosuke Yura, Jui Chun Wang, Yukimi Fujii, Akiko Nakanishi, Takashi Shimizu, and Masahiko Hibi

We would like to express our gratitude to Reviewers 1 and 2 for their high appraisal for the revised manuscript. While Reviewer 2 did not request any further revisions, Reviewer 1 had some comments. Therefore, we will explain our responses on a point-by-point basis.

Reviewer 1 Comments for the Author:

© 2024. Published by The Company of Biologists under the terms of the Creative Commons Attribution License (https://creativecommons.org/licenses/by/4.0/).

The revised version of the manuscript by Itoh et al. reads well and has covered well most of my points of concern. The generation of the knock-in line of GFP into the endogenous locus of ptf1a is very convincing and demonstrates well the specificity of Cre expression in ptf1a-expressing cells.

The expression data shown in Table 1-3 is very comprehensive yet the authors did not address my concern that their evaluation of expression strength of some of these markers is based on a very small number of larvae analyzed. In some cases only a single larva was analyzed. Maybe this is a misunderstanding on my side. Does n=1 mean one staining procedure with several larvae? Then the number of larvae studied should be provided.

In response to the reviewer's comments, at the time of revision, we conducted quantitative analysis using ImageJ/Fiji on available image data from *in situ* hybridization and immunohistochemistry, and indicated the number of larvae used as 'n' in the table. Although we did not retain the image data, there were instances where similar results were observed in other larvae. The combined number of such larvae has been included in the table, within brackets []. This information has been noted in the table legend.

We believe that we have presented sufficient data to support our conclusions. However, should the reviewer deem the sample size to be too small, we are prepared to increase it. Please note that for some samples, it may take more time as it will require the use of the next generation of zebrafish mutants.

Minor points:

Some of the selected references should be corrected or updated. For example:

Line 63/64 Expression of atoh1 and ptf1a genes in the URL and VZ of the cerebellum was also reported for zebrafish (Chaplin et al., 2010, Kani et al., 2010). Expression of atoh1 genes in the URL has been reported earlier. e.g. Köster et al., 2001, Adolf et al., 2004, Volkmann et al., 2008.
We are grateful for the suggestions from the reviewer. We have cited all the papers that were suggested.

Line 364: …and SHH signaling is not activated in the zebrafish cerebellum (Biechl et al., 2016..). Biechl et al. showed shh expression in eurydendroid cells in the cerebellum (see supplementary data).

In the paper by Biechl et al., 2016, it is shown that *shh* is weakly expressed in eurydendroid cells, but its expression in Purkinje cells is denied. The paper by Chaplin et al., 2010, demonstrates the absence of *shh* expression in Purkinje cells and the absence of *ptc1*, a target of Shh signaling, in the cerebellum. We think that we have not made any incorrect citations, but if the reviewer considers any of our citations to be misleading, we are open to making corrections.

In this study, we used HEK293T cells and, prior to publishing, we confirmed that they are derived from HEK293 through STR profiling. We have conducted a mycoplasma infection check using cell culture supernatant and found that HEK293T cells are infected with mycoplasma. However, in this experiment, HEK293T cells were used solely for protein expression, and we do not believe that the mycoplasma infection significantly affects the results of protein interactions (Fig. S13). Nevertheless, if it is necessary to replicate the experiment with mycoplasma-free cells, we are willing to do so.

The manuscript slightly exceeds the 7000-word limit, but we have trimmed it as much as possible. Additionally, during the revision, we conducted experiments in response to the reviewer's comments, which led to some added descriptions, making it difficult to shorten it further. However, if it is essential to reduce the length further, we are willing to consider it.

Second decision letter

© 2024. Published by The Company of Biologists under the terms of the Creative Commons Attribution License (https://creativecommons.org/licenses/by/4.0/).

MS ID#: DEVELOP/2023/202546

MS TITLE: Foxp- and Skor-family proteins control differentiation of Purkinje cells from Ptf1a and Neurogenin1-expressing progenitors in zebrafish

AUTHORS: Tsubasa Itoh, Mari Uehara, Shinnosuke Yura, Jui Chun Wang, Yukimi Fujii, Akiko Nakanishi, Takashi Shimizu, and Masahiko Hibi

I am happy with your revisions and only have one minor suggestion before we proceed to publication. I suggest you consider adding a sentence or two to the methods to explain that in some cases only a subset of the embryos showing an expression phenotype were quantitatively assessed - I know you make a statement along these lines in the figure legend but I think it could perhaps be stated more clearly in the methods section. On the issue of mycoplasma, I agree that I can't see that this would affect the outcome of your experiments. Your word count is OK.

Second revision

Author response to reviewers' comments

Following your suggestion, we have added the sentence “In some cases, only a subset of the larvae showing an expression phenotype were qualitatively assessed” in the “Quantification of image data” of the MATERIALS and METHODS section.

Third decision letter

MS ID#: DEVELOP/2023/202546

MS TITLE: Foxp- and Skor-family proteins control differentiation of Purkinje cells from Ptf1a and Neurogenin1-expressing progenitors in zebrafish

AUTHORS: Tsubasa Itoh, Mari Uehara, Shinnosuke Yura, Jui Chun Wang, Yukimi Fujii, Akiko Nakanishi, Takashi Shimizu, and Masahiko Hibi

ARTICLE TYPE: Research Article

I am happy to tell you that your manuscript has been accepted for publication in Development, pending our standard ethics checks.

© 2024. Published by The Company of Biologists under the terms of the Creative Commons Attribution License (https://creativecommons.org/licenses/by/4.0/).
